# Conformational Dynamics of the Nucleosomal Histone H2B Tails Revealed by Molecular Dynamics Simulations

**DOI:** 10.1021/acs.jcim.4c00059

**Published:** 2024-06-12

**Authors:** Rutika Patel, Augustine Onyema, Phu K. Tang, Sharon M. Loverde

**Affiliations:** † Ph.D. Program in Biochemistry, The Graduate Center of the City University of New York, New York, New York 10016, United States; ‡ Department of Chemistry, College of Staten Island, The City University of New York, 2800 Victory Boulevard, Staten Island, New York, New York 10314, United States; § Ph.D. Program in Chemistry, The Graduate Center of the City University of New York, New York, New York 10016, United States; ∥ Ph.D. Program in Physics, The Graduate Center of the City University of New York, New York, New York 10016, United States

## Abstract

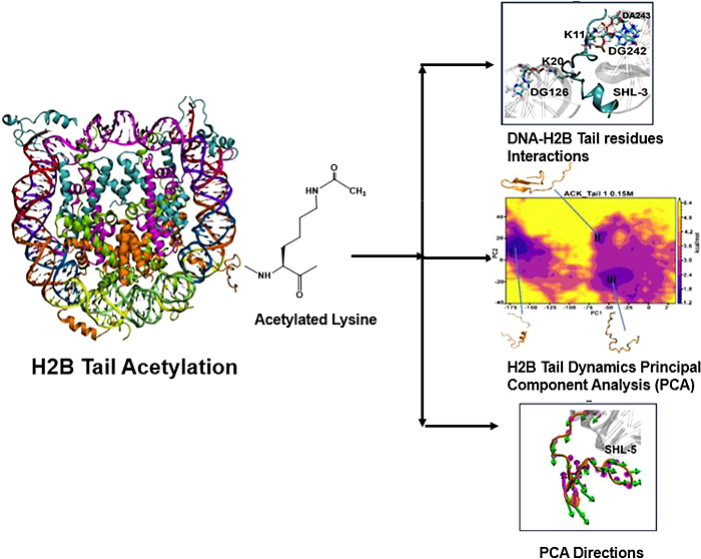

Epigenetic modifications of histone N-terminal tails play a critical role in regulating the chromatin structure and biological processes such as transcription and DNA repair. One of the key post-translational modifications (PTMs) is the acetylation of lysine residues on histone tails. Epigenetic modifications are ubiquitous in the development of diseases, such as cancer and neurological disorders. Histone H2B tails are critical regulators of nucleosome dynamics, biological processes, and certain diseases. Here, we report all-atomistic molecular dynamics (MD) simulations of the nucleosome to demonstrate that acetylation of the histone tails changes their conformational space and interaction with DNA. We perform simulations of H2B tails, critical regulators of gene regulation, in both the lysine-acetylated (ACK) and unacetylated wild type (WT) states. To explore the effects of salt concentration, we use two different NaCl concentrations to perform simulations at microsecond time scales. Salt can modulate the effects of electrostatic interactions between the DNA phosphate backbone and histone tails. Upon acetylation, H2B tails shift their secondary structure helical propensity. The number of contacts between the DNA and the H2B tail decreases. We characterize the conformational dynamics of the H2B tails by principal component analysis (PCA). The ACK tails become more compact at increased salt concentrations, but conformations from the WT tails display the most contacts with DNA at both salt concentrations. Mainly, H2B acetylation may increase the DNA accessibility for regulatory proteins to bind, which can aid in gene regulation and NCP stability.

## 1 Introduction

The nucleosome core particle (NCP) is the fundamental repeat unit of chromatin^1^ that packages DNA in the nucleus of the eukaryotic cells. The NCP consists of about 147 base pairs of DNA wrapped around the histone octamer with 1.65 superhelical turns in a left-handed manner. The histone octamer is composed of two copies of H3, H4, H2A, and H2B. Together with histone H1 and linker DNA, they further assemble into higher-order structures of chromatin, which are compact and dynamic.^1−6^ The NCP is stabilized by electrostatic interactions between the negatively charged DNA phosphate backbone and positively charged histone residues such as lysine (Lys, K) and arginine (Arg, R). The assembly of the NCP involves the heterodimerization of histone H3 and H4 proteins that form a stable tetramer, which then combines with H2A and H2B histone proteins. All four histone proteins of the octamer are composed of both a globular core and a disordered tail region.^7−9^

Due to their highly charged nature, the tails are restricted from the formation of packed globular structures, but despite that, they are capable of secondary structure formation.^10−13^ Histone tails are highly positively charged and interact with the negatively charged phosphate backbone of DNA by forming salt bridges. Any changes in the tails in the process of post-translational modifications (PTMs) can fundamentally perturb these interactions.^14,15^ The ionic environment around the nucleosome modulates the electrostatic interactions based on the salt concentration.^15^ Previous X-ray studies have indicated the disordered nature of the histone tails, although the transient binding of the tails may affect transcription factor access.^16^ These disordered histone N-terminal tails that protrude from the histone core lack a well-determined structure, yet perform several critical biological functions. The tails are also involved in various chromatin functions, including mediating the formation of compact 30 nm chromatin fibers through internucleosome contacts.^9^ In addition, the tails play key roles in nucleosome stability and dynamics, DNA accessibility, nucleosome sliding and repositioning, and coordination of various epigenetic regulation pathways in time. The tails speed up the search for nucleosome targets to ease the interactions. The tails are also involved in DNA unwrapping, which is regulated by the local salt concentration.

The N-terminal tails are major targets for post-translational modifications (PTMs) that usually involve acetylation, phosphorylation, methylation, ubiquitination, etc., and are responsible for biological processes such as transcription, DNA repair, chromosome packaging, and DNA replication. PTMs can alter the highly ordered chromatin structure to allow protein modulators access to the DNA.^17,18^ Also, the histone tails exhibit distinct conformational states characterized by differences in secondary structure propensity.^13^ Out of all PTMs, Lysine acetylation of the N-terminal histone tails is a well-characterized epigenetic mark that neutralizes the positive charge of lysine by replacing it with an acetyl (−CO–CH_3_) group and is essential for several biological processes, including transcription, DNA repair, chromatin remodeling, and nucleosome sliding. Histone acetyltransferases (HATs) transfer the acetyl group from acetyl-Coenzyme A (acetyl-CoA) to the ε-amino group of the lysine side chains, which neutralizes the positive charge of the lysine residues.^19^ As the positively charged residues interact with DNA’s negatively charged phosphate backbone, acetylation weakens these interactions and causes chromatin to transition from tightly packed to loosely packed. This would make DNA more accessible to transcription factors and other proteins.^20^ Epigenetic modifications are linked with various diseases, including cancer, neurological disorders, and inflammatory diseases.^21,22^ One of the histone proteins, H2B, is associated with transcription activation^23−25^ and DNA repair.^26^ Herein, we focus on the effects of acetylation on the conformational dynamics of the H2B tails, focusing on key lysine residues whose acetylation may impact transcription, using molecular dynamics simulations.

Molecular dynamics (MD) simulations have elucidated several aspects of nucleosome dynamics. One of the earliest all-atomistic MD simulations was conducted for the 1KX3 NCP system to understand nucleosomal DNA conformations for 10 ns by Bishop et al.^27^ Another early study included an estimation of the elastic properties of DNA for 300 ns in the 1KX5 NCP system by Garai et al.^28^ A study of the 1KX5 NCP for 20 ns with and without N-terminal histone tails was performed by Roccatano et al.^29^ This study determined that kinks and bends in the DNA remained the same, with and without the tails. In contrast, the N-terminal tails showed conformational rearrangements that can modulate DNA accessibility. Also, Ruscio et al.^30^ provided insight into large-scale structural fluctuations, such as significant bending of the nucleosomal DNA through implicit solvent molecular dynamics simulations. All earlier studies were done at nanosecond time scales due to computational power limitations for large assemblies like the NCP. Most NCP dynamics usually occur at microseconds to seconds time scales. Later studies of the NCP involved MD simulations at several nanoseconds to microseconds time scales.^6^ Biswas et al.^31^ determined, based on a total of 800 ns simulation of the 1KX5 NCP, by studying histone tail truncations, that the H3 and H2B tails alter DNA-tail interactions. This alters nucleosome stability as well as the propensity of helix formation of the tails. Several MD simulation studies have focused on DNA motion, such as DNA breathing.^32−37^ Other MD studies have included the role of histone tails^38,39^ that allow for DNA sliding. Also, enhanced sampling techniques have been used to study nucleosome unwrapping using adaptively biased force MD and Umbrella Sampling. These methods have determined that the unwrapping process may occur via asymmetric and symmetric pathways.^34,40^ Furthermore, some MD simulation studies have been performed to characterize the effects of acetylation, which mainly involves histone H3 and H4 tails.^41−49^ The acetylation of H3 tail Lys14 residue showed destabilization of NCP with weakened DNA–tail interactions and exposed DNA for other regulatory proteins to bind^50^ as well as H4 tail Lys16 acetylation decreases heterogeneity of the tail conformations, providing an elongated structure of the tail.^42^ Thus, all-atomistic MD simulations are a useful tool to characterize NCP structure and dynamics.

Previous molecular dynamics (MD) simulation studies conducted by our laboratory included characterization of the nucleosomal DNA partial unwrapping at 2 M salt concentration at microseconds time scales for the 1KX5 system.^51^ We demonstrated that the destabilization of the H2B N-terminal tail promotes the outward stretching of the SHL-5 region of DNA. We also reported a wave-like motion of the DNA, resulting from the stabilizing effects of the H2A and destabilizing effects of the H2B tails to form an energetically less expensive loop. Based on these findings, it was suggested that the highly charged and flexible histone tails can act as switches to maintain the stability and plasticity of the nucleosome.^34^ Following, we reported a study comparing the motions of the Widom-601 and alpha satellite palindromic nucleosomal DNA sequences at time scales of 12 μs. We found fundamentally different pathways for the two sequences with one forming a loop and the other exhibiting large-scale breathing. The motion and contacts of the neighboring tails H2A and H2B play a critical role in loop formation, while the H3 tail plays a key role in breathing.^52^ Thus, post-translational modifications (PTMs) may fundamentally alter the dynamics of the tails and may impact the kinetics of the initial stages of DNA unwrapping.

Several experimental studies have been performed to study the flexible and disordered tails to understand the tail dynamics and NCP stability. A small-angle-X-ray scattering (SAXS) and fluorescence resonance energy transfer (FRET) study demonstrated that removing the H3 tail destabilizes the NCP, leading to unwrapping. Also, the H4 tail removal causes DNA to be tightly bound to the histone octamer, indicating that unwrapping is distinctly influenced by tails.^53^ A single-molecule FRET study showed that acetylation increases nucleosome unwrapping.^54^ Nuclear magnetic resonance (NMR) studies by Kim et al.^19^ suggested that acetylation of four histone tails causes subtle changes in NCP dynamics. There is an increase in the motions of the acetylated tails and DNA accessibility for regulatory proteins.^19^ Further NMR studies of the NCP have provided insights into tail interaction with proteins.^55−58^ Zhao et al.^56^ demonstrated that acetylation in the H3 and H4 tails decreased the compaction of nucleosomal arrays. Acetylation increased the dynamics of the tails and promoted regulatory protein interactions, as characterized using NMR spectroscopy.^56^ It has also been shown that acetylation of one of the H4 tail residues can enhance the acetylation rate for the H3 tail using NMR.^57^ Circular dichroism (CD) and NMR have also characterized the secondary structure conformations of the histone tails.^59−61^ Thus, there have been significant experimental studies of the effect of acetylation on the histone tail dynamics.

Here, we perform two sets of 1 μs long all-atomistic molecular dynamics (MD) simulations of the nucleosome core particle to investigate the effects of lysine acetylation on H2B tail dynamics and nucleosome stability at physiological 0.15 and 2.4 M salt concentrations. The sets of simulations that we perform for the NCP include three sets each of 0.15 M unacetylated (WT_0.15M) H2B tails, 0.15 M lysine-acetylated (ACK_0.15M) K5, K12, K15, and K20 residues of H2B tails, and two sets of each 2.4 M unacetylated (WT_2.4M) and 2.4 M lysine-acetylated (ACK_2.4M) K5, K12, K15, and K20 residues of H2B tails. Upon acetylation of lysine residues on the H2B tails, the tail is released from the nucleosomal DNA as charge neutralization reduces the number of contacts between DNA and histone. In the WT, the tail collapses on the DNA surface, with an increased number of contacts. We also find that acetylation of the H2B tails leads to secondary structure rearrangements. Complemented with this secondary structure analysis, principal component analysis (PCA) unveils distinct conformational states of the H2B tails. We find that the acetylation of H2B tails causes a reduction in the number of contacts between the DNA and the H2B tail. Weakening these interactions destabilizes the NCP structure, making the chromatin structure more open, allowing access to DNA for regulatory proteins for transcription or other biological processes.^62,14^

## 2 Simulation

The nucleosome core particle (NCP) was simulated in a physiological salt concentration of 0.15 M NaCl and also a high salt concentration of 2.4 M NaCl. The initial configuration was obtained from the crystal structure,^51^ as reported in the Protein Data Bank (PDB ID: 1KX5). Both subunits of H2B histone N-terminal at K5, K12, K15, and K20 positions were acetylated by adding an acetyl group (−CH_3_–CO−) using PyMOL.^63^ The histone including acetylated H2B tails (ACK) and wild-type (WT) unacetylated H2B tails were simulated using AMBER force fields. The histone of the NCP was simulated with ff19SB,^64^ and the DNA using OL15.^65^ We note that further studies of tail dynamics with force fields such as those developed by Shaw and colleagues^66,67^ could help add further insight into histone tail dynamics. The OPC water model^68^ was used, with its Lennard-Jones interaction (Na^+^/OW) modification, using the Kulkarni et al. method that provides better estimates of the osmotic pressure.^69^ The OPC water model has been shown to improve atomistic simulations of intrinsically disordered peptides.^70^ For sodium (Na^+^) and chlorine (Cl^–^) ions, Joung and Cheetham^71^ parameters were used. Mg^2+^ modification was performed using the Li et al. parameter method.^72^ The ACK parameters were used for lysine acetylation, as published by Papageorgiou.^73^ All force fields were sourced using the tleap module of AmberTools21 to create the topology and coordinate files for the initial ACK and systems. The total number of molecules and water/ions are shown in Table S1. All systems were initially minimized and equilibrated for 100 ns, followed by production runs for 1 μs using Amber18.^74^ One of the production runs for a single simulation replica was performed on the Anton-2^75^ supercomputer. All systems were initially minimized to reduce unfavorable stress by using a conjugate gradient and steepest descent gradient for 40 ps. Following minimization, the heating was performed by increasing the temperature of the system to 310 K under *NVT* conditions. Afterward, the systems were equilibrated for 100 ns under the *NPT* conditions. The Langevin^76^ dynamics method with the collision frequency with a friction constant of 1 ps^–1^ was used to control the temperature of the system. The pressure of the system was controlled by the Berendsen^77^ barostat. The simulation was continued for 1 μs under *NPT* conditions with a 2 fs each time step. Trajectories were stored after every 200 ps for analysis. All simulations used the SHAKE^78^ algorithm to constrain the bonds involving hydrogen. The Lennard-Jones cutoff value for nonbonded interactions was 12 Å, and electrostatic interactions were treated with the Particle Mesh Ewald (PME)^79^ method with full periodic boundary conditions.

## 3 Methods

### 3.1 Root-Mean-Square Deviations (RMSDs)

The RMSDs of the WT and ACK systems are calculated with respect to the initial structure throughout 1 μs. The H2B tail region includes 4–30 amino acid residues. The RMSDs of H2B tail residues are calculated using MDAnalysis.^80,81^ RMSDs are computed using C_α_ atoms of each amino acid residue of the H2B tails.^82^

### 3.2 Radius of Gyration (*R*_g_)

The radius of gyration (*R*_g_) is calculated using C_α_ atoms of H2B tail residues for both the WT and ACK systems. The radius of gyration indicates the compactness of protein structure as it measures the average distance of the sum of the root-mean-square distance of each atom from its center of mass.^83^ The radius of gyration is calculated as  where *m_i_* is the mass of the *i*^th^ atom in the particle and *r_i_* is the distance from the center of the mass to the *i*^th^ particle using MDAnalysis^81^ for the entire 1 μs trajectory for both WT and ACK H2B tail residues. Simple scaling relations for globular and thermally denatured proteins are compared with the computationally measured *R*_g_.^13^ The predicted *R*_g_ for the globular state of proteins of the H2B tail residues (4–30) is 7.69 Å. This value is based on *R*_g,glob_ (N) = 2.2 N^0.38^, a relation based on a power law best fit of *R*_g_ as a function of sequence length for a set of globular proteins in the PDB database.^13,84^ Similarly, the predicted *R*_g_ for a thermally denatured random coil is 14.6 Å. This value is based on the denatured random coil, *R*_g,denat_ (N) = 2.02 N^0.60^.^13,85^ Similarly, the predicted *R*_g_ values for the globular state for H3 (residues 1–43), H4 (residues 1–23), and H2A (residues 1–15) N-terminal tails are 9.18 7.24, and 6.15 Å, respectively. The predicted *R*_g_ values for the denatured coil state for H3, H4, and H2A N-terminal tails are 19.29 13.25, and 10.25 Å, respectively.

### 3.3 Solvent Accessible Surface Area (SASA)

To analyze the accessibility of the tails in WT and ACK systems, SASA is calculated using VMD^86^ which uses the Lee and Richards^87^ algorithm. We calculate the SASA for the H2B tail residues of the WT and ACK systems. SASA characterizes protein stability and conformational changes.^87,88^ Here, we compute the time-dependent SASA of the H2B tail residues over 1 μs. SASA analysis provides insight into DNA–histone tail interactions, as the higher SASA value indicates the tail is dissociated from DNA and more solvent exposed.

### 3.4 Secondary Structures Analysis

The secondary structure of both the WT and ACK H2B tail residues is determined using the AmberTools21 secstruct tool, which uses the DSSP^89^ algorithm. This algorithm is based on hydrogen bonding patterns in the protein backbone amide (N–H) and carbonyl (C=O) positions. The algorithm provides secondary structure including α-helix, 3_10_-helices, turns, β-sheets, coils, or no structure. The secondary structure propensity for each residue in the H2B tail is averaged over the trajectory.

### 3.5 DNA–Histone Tail Contacts

We calculated the number of DNA-histone contacts over 1 μs. The number of contacts is calculated with a distance cutoff value of 4.5 Å between two heavy atoms of DNA and H2B tail residues. The contact maps are obtained based on the same distance cutoff using MDAnalysis.^81^

### 3.6 Principal Component Analysis (PCA)

PCA is a dimensionality reduction technique that can be used to identify global motions. PCA identifies the configurational space that contains only a few degrees of freedom in which anharmonic motion occurs. PCA maps the coordinates in each trajectory frame to a linear combination of orthogonal vectors. The configurational space can be built using a simple linear transformation in Cartesian coordinate space to generate a 3*N* × 3*N* (*N* = number of atoms) covariance matrix (*C*) and is diagonalized. The elements of *C* are shown as *C*_*ij*_ = ⟨(*x_i_* – ⟨*x*_*i*_⟩)(*x_j_* – ⟨*x*_*j*_⟩)⟩. where *x*_1_,...,*x*_3*N*_ are the Cartesian coordinates of an *N*-particle system and surrounding brackets correspond to the average over time. The diagonalization of matrix *C* generates eigenvectors that provide a vectorial description of each component by indicating the direction of motion. The matrix *C* is equivalent to solving the eigenvalues as *R*^T^*CR* = λ, where λ_1_ ≥ λ_2_ ≥···≥ λ_3*N*_ are eigenvalues and *R*^T^ is the transpose of *R*. The columns of *R*, the eigenvectors, provide the vectorial description of the motion by indicating the direction of the motion. The trajectory can be projected onto the eigenvectors to obtain the principal components (PC). Here, *q*_*i*_(*i*), *i* = 1, 2,...,3*N*, and related equation is *q* = **R**^T^(*x*(*t*) – ⟨*x*_*j*_⟩). The eigenvalues λ_*i*_ represent the fluctuation along the direction of the *i*th principal component. The largest eigenvalues correspond to the largest number of variations. Therefore, the first few principal components with the highest variations describe the collective global motions of the system.^90,91^ Here, WT and ACK systems’ H2B tail residues are used to calculate the principal components (PC). The covariance matrix of atomistic variations of C_α_ atoms of H2B tail residues is built and diagonalized to obtain the PCs. The first two PCs are used to calculate probability density to plot the two-dimensional free energy landscape. The free energy landscape is defined as using Δ*G*(*x*,*y*) = −*RT* ln[*P*(*x*,*y*)/*P*_max_], where *P*(*x*,*y*) is the probability density distribution, *R* is the gas constant, *T* is the temperature (310 K), and *P*_max_ is the maximum probability. The direction of the PCA modes of H2B WT and ACK tails are visualized through porcupine plots using NMWiz^92^ in VMD.^86^

## 4 Results

### 4.1 Nucleosome Structure and Flexibility of the H2B Tail upon Acetylation

**Figure 1:**
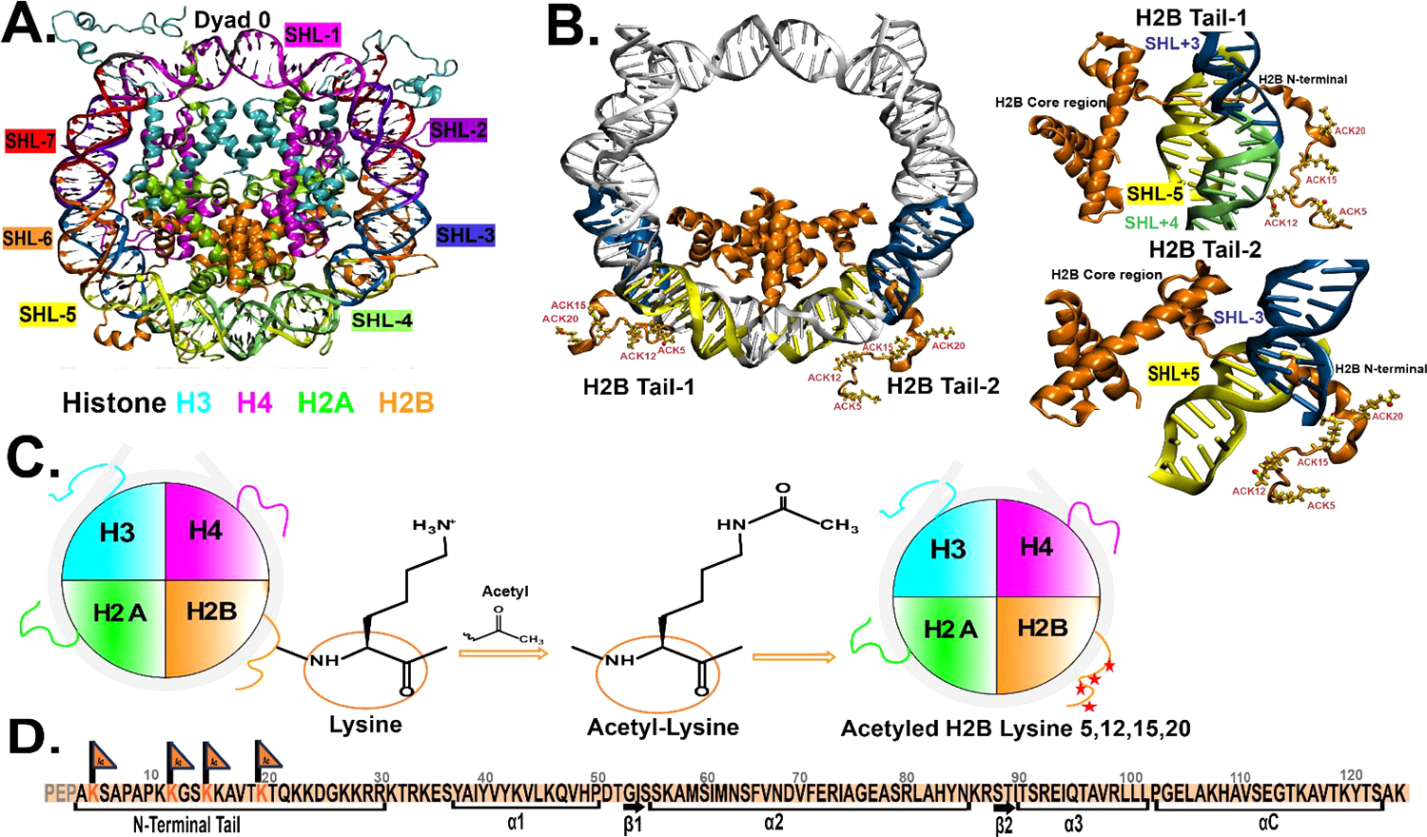
Overview of nucleosome core particle (NCP) structure and acetylation. (A) Crystal structure of NCP (PDB ID: 1KX5) consists of 147 DNA base pairs wrapped around two copies of the histone proteins H3 (cyan), H4 (magenta), H2A (green), and H2B (orange). The super helix locations (SHLs) of DNA are indicated with different colors; each color corresponds to each SHL region labeled on the outside. (B) NCP shows H2B tail-1 (left) and H2B tail-2 (right) protruding between two DNA gyres around SHL ± 5 (yellow) and SHL ± 3 (blue) regions. DNA gyres are shown in gray, and the H2B core and tail regions are shown in orange. The acetylated lysine (ACK) residues 5, 12, 15, and 20 are shown and labeled. The two figures on the right show the best representation of the H2B tail-1 and 2 with core regions (orange) and DNA SHL ± 5 (yellow) and SHL ± 3 (blue) regions. (C) General scheme of the acetylation process shows the disc structure of the NCP of the 1KX5 system with histones H3 (cyan), H4 (magenta), H2A (green), H2B (orange), and nucleosomal DNA (gray). The schematic diagram shows H2B tail lysine acetylation. The positive charge of the lysine is replaced by the acetyl (−CH_3_CO) group, making acetyl-lysine (ACK), and the four red stars at the end on the H2B tail (orange) show four lysine residues of the tail that are neutralized through acetylation. (D) Sequence of H2B with an N-terminal tail includes the residue numbers and sites for acetylated lysine (orange flags). The first three residues PEP of the H2B tail in 1KX5 PDB structure are absent; therefore, the N-terminal tail is considered from residue 4 to 30 amino acid residues.

Here, we perform multiple 1 μs molecular dynamics (MD) simulations of the nucleosome core particle (NCP) of the 1KX5 nucleosome system. We perform three simulation replicas of the following NCP systems for 1 μs time scales at 0.15 M salt concentration: 0.15 M unacetylated (WT_0.15M) H2B tails, 0.15 M lysine-acetylated (ACK_0.15M) K5, K12, K15, and K20 residues of H2B tails, and two replicas at 2.4 M salt concentration: 2.4 M unacetylated (WT_2.4M) and 2.4 M lysine-acetylated (ACK_2.4M) K5, K12, K15, and K20 residues of H2B tails. We compare the conformational dynamics of the unacetylated H2B N-terminal tail (WT) to the H2B N-terminal tail (ACK) acetylated at key lysine residues. The simulations are performed at a physiological 0.15 M NaCl concentration and at ∼15 times higher than the physiological concentration, at 2.4 M, to observe the effects of the ion concentration on regulating tail conformations and dynamics. Supplementary Table 1 summarizes the simulation setups. The structure of the NCP 1KX5 system consists of DNA and histone proteins, as shown in Figure 1A. The NCP (PDB ID: 1KX5(51)) consists of 147 DNA base pairs wrapped around two copies of the histone proteins H3, H4, H2A, and H2B. The crystal structure of the 1KX5 system was first characterized by Davey et al.^51^ at 1.9 Å resolution. The 1KX5 system has the N-terminal tails resolved in the crystal structure for histone proteins, including the H2B N-terminal tails. The orientation of the DNA base pairs of the NCP is usually represented relative to the central base pair, known as superhelical location (SHL) zero. Each SHL region consists of approximately ten base pairs (Figure 1A) The superhelical location is given where the major groove faces the histone octamer.^93^ The first is SHL 0 (at the NCP dyad), and the last is SHL ± 7. As there are two H2B N-terminal tails in the NCP, the left H2B tail is represented as H2B tail-1 (H2B1), and the right H2B tail is represented as H2B tail-2 (H2B2) (Figure 1B). The H2B tail residues (4–30) are primarily present around the two DNA gyres around SHL ± 5 and SHL ± 3 for both tails (Figure 1B, D). The lysine (Lys, K) residues K5, K12, K15, and K20 of both H2B tails are acetylated by adding the acetyl group, neutralizing the positive charge of these lysine residues (Figure 1C, D).

**Figure 2:**
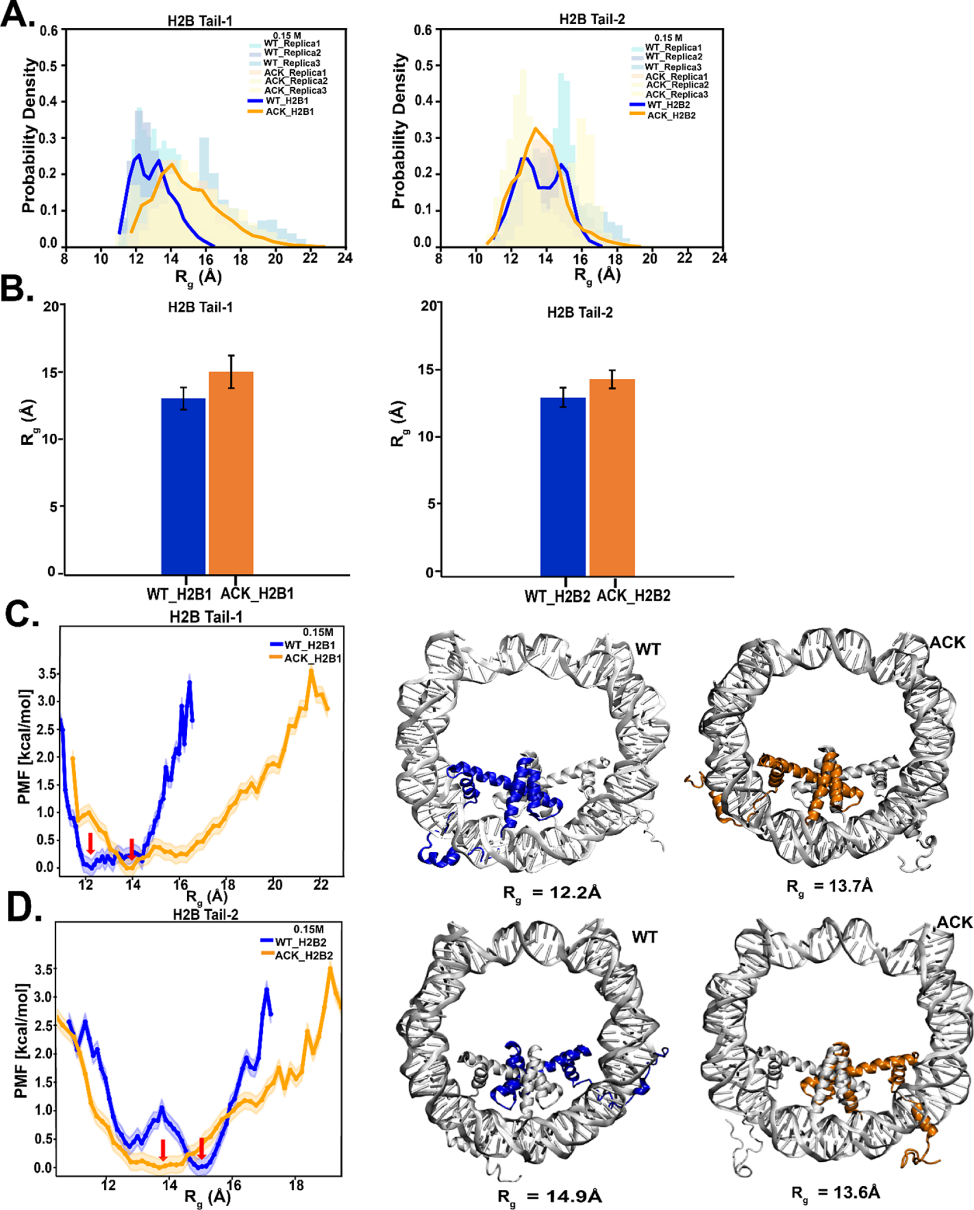
Radius of gyration (*R*_g_) of the H2B N-terminal tail upon acetylation. (A) Radius of gyration (*R*_g_) of H2B N-terminal tails at 0.15 M NaCl concentration are calculated based on C_α_ of the tail residues over 1 μs simulation. The H2B Tail-1 (H2B1) probability density distribution of the ACK tail (orange) is extended compared to WT (blue). The H2B Tail-2 (H2B2) probability density distribution shows a slightly extended tail upon acetylation (orange) compared to WT (blue). The histogram shows the distribution for three replicas, and the solid line represents the average of *R*_g_ for replicas. (B) Average *R*_g_ of three replicas for H2B tails for WT and ACK systems at 0.15 M salt concentrations is obtained by dividing the data into nonoverlapping blocks nonoverlapping blocks using 11 blocks approximately 91 ns per block for 1 μs simulation. (C and D) Potential of mean force (PMF) as a function of the radius of gyration (*R*_g_) for both H2B Tail-1 and Tail-2 for WT (blue) and ACK (orange) is calculated based on PMF = −*K*_b_*T* log(*P*/*P*_max_). The configurations of H2B tails for both the WT (blue) and ACK (orange) systems are shown with their corresponding *R*_g_ values.

Next, we characterize the structural conformations of the H2B tail for both the WT and ACK systems at both salt conformations using RMSD and *R*_g_. At 0.15 M salt concentration, the root-mean-square deviation (RMSD) of the H2B tail is calculated based on the C_α_ of all tail residues with respect to the crystal structure. The probability distribution and the block average of the simulation replicas of the H2B tail-1 show higher RMSD values upon acetylation than the WT. Similarly, H2B tail-2 shows slightly higher RMSD upon acetylation, with some overlap of the probability distribution compared to the WT (Figure S1A,B). Upon acetylation, the tail is released from the DNA, which makes it more solvent-exposed and dynamic (Movies S1 WT and S2 ACK). At 2.4 M salt concentration, the RMSD probability distribution and the averages of the simulation replicas of the acetylated H2B tail overlap with the WT for tail-1, with the lower average for the acetylated tail compared to WT. The acetylated H2B tail-2 is more compact than the WT, as RMSD values are lower than the WT (Figure S2A,B). Next, the radius of gyration (*R*_g_), which indicates the relative compactness of the tail^83^ is shown for both the WT and ACK systems at 0.15 M salt concentration (Figure 2A, B). We analyze *R*_g_ for simulation replicas of all systems. The acetylated H2B tails have slightly higher *R*_g_ compared to the WT. The potential of mean force (PMF) is obtained from the probability distribution for the WT and the ACK tails shown in Figure 2C, D. For both tails, the minima in the PMF plots are indicated by the red arrow. For tail-1, the *R*_g,min_ is 12.2 and 13.7 Å for WT and ACK, respectively. This indicates an increase in the *R*_g_ upon acetylation. The predicted *R*_g,glob_ for the globular state is 7.69 Å, and the *R*_g,denat_ denatured state is 14.6 Å. The acetylated tail-1 has an *R*_g,min_ of 13.7 Å, close to that of the denatured state. For tail-2, the *R*_g,min_ is 14.9 and 13.6 Å for WT and ACK. Both values for *R*_g_ are closer to the predicted *R*_g_ for the denatured state. For the 2.4 M salt concentration, the ACK tails show a decrease in their *R*_g_ values compared to those of the WT (Figure S3A,B). The PMF plot shows the respective minima (Figure S3C,D). For tail-1, the *R*_g_,_min_ is 12.4 and 10.5 Å for WT and ACK, respectively. For tail-2, the *R*_g,min_ is 15.6 and 12.1 Å for WT and ACK. The predicted *R*_g,glob_ for the globular state is 7.69 Å, and the *R*_g,denat_ denatured state is 14.6 Å. The acetylated tail-1 has a *R*_g,min_ of 10.5 Å, close to the globular state. The corresponding conformations for the tails are shown in Figure 2C, D for 0.15 M, and in Figure S3C,D for the 2.4 M salt concentration. Thus, upon acetylation, the H2B tails increase their radius of gyration. However, the trend at higher salt requires further investigation using a method such as a replica exchange molecular dynamics as reported by Potoyan and Papoian.^13^ Notably, at high salt concentrations, we have previously reported the collapse of the H2B tail.^34,52^

We also characterize the *R*_g_ for histone tails H3, H4, and H2A for the WT system at both salt concentrations including simulation replicas (Figures S4A–C and S5A–C). The predicted *R*_g_ values for globular and disordered states for H3 (residues 1–43) are 9.18 and 19.29 Å. For H3 tail-1 and tail-2, the *R*_g_ of 11 Å at 2.4 M salt concentration indicates compaction, being close to the predicted value of *R*_g,glob_ of 9.18 Å (Figure S5A). This is consistent with that which we reported for the collapse of the H3 tail at high salt concentration.^52^ At 0.15 M salt concentration (Figure S4A), for the H3 tail-1, the *R*_g_ exhibits an average of around 12 Å, close to a globular state. However, for tail-2 at 0.15 M salt concentration, the *R*_g_ of around 15 Å indicates that the tail is close to a disordered state. Thus, we note the trend of compaction at high salt. For the H4 tail (residues 1–23), the predicted *R*_g_ values for globular and disordered states are 7.24 and 13.25 Å. H4 tail-1 shows an equal *R*_g_ range at both salt concentrations, with an average of around 12 Å, close to a disordered state (Figures S4B and S5B). For tail-2, the average is similar for both salt concentrations at ∼12 Å, which is also close to the disordered state. At 0.15 M salt concentration, there is an additional peak in *R*_g_ at ∼8 Å, which is close to the globular state (tail-2, Figure S4B)_._ This is consistent with previously reported computational results by Potoyan et al.^13^ They suggested that H4 is a half-ordered and half-disordered tail. The predicted *R*_g_ values for globular and disordered states for H2A (residues 1–15) are 6.15 and 10.25 Å. For H2A tail-1, the average *R*_g_ for both salt concentrations is around 9 Å, indicating a disordered state (Figure S4C and S5C). The average *R*_g_ for H2A tail-2 at both salt concentrations is around 7 Å (Figures S4C and S5C), close to that of the globular state. For all histone tails, despite salt-dependent shifts in *R*_g_ values, the histone tails show both globular and disordered states.

### 4.2 Structural Rearrangement of the H2B N-Terminal Tail upon Acetylation

**Figure 3:**
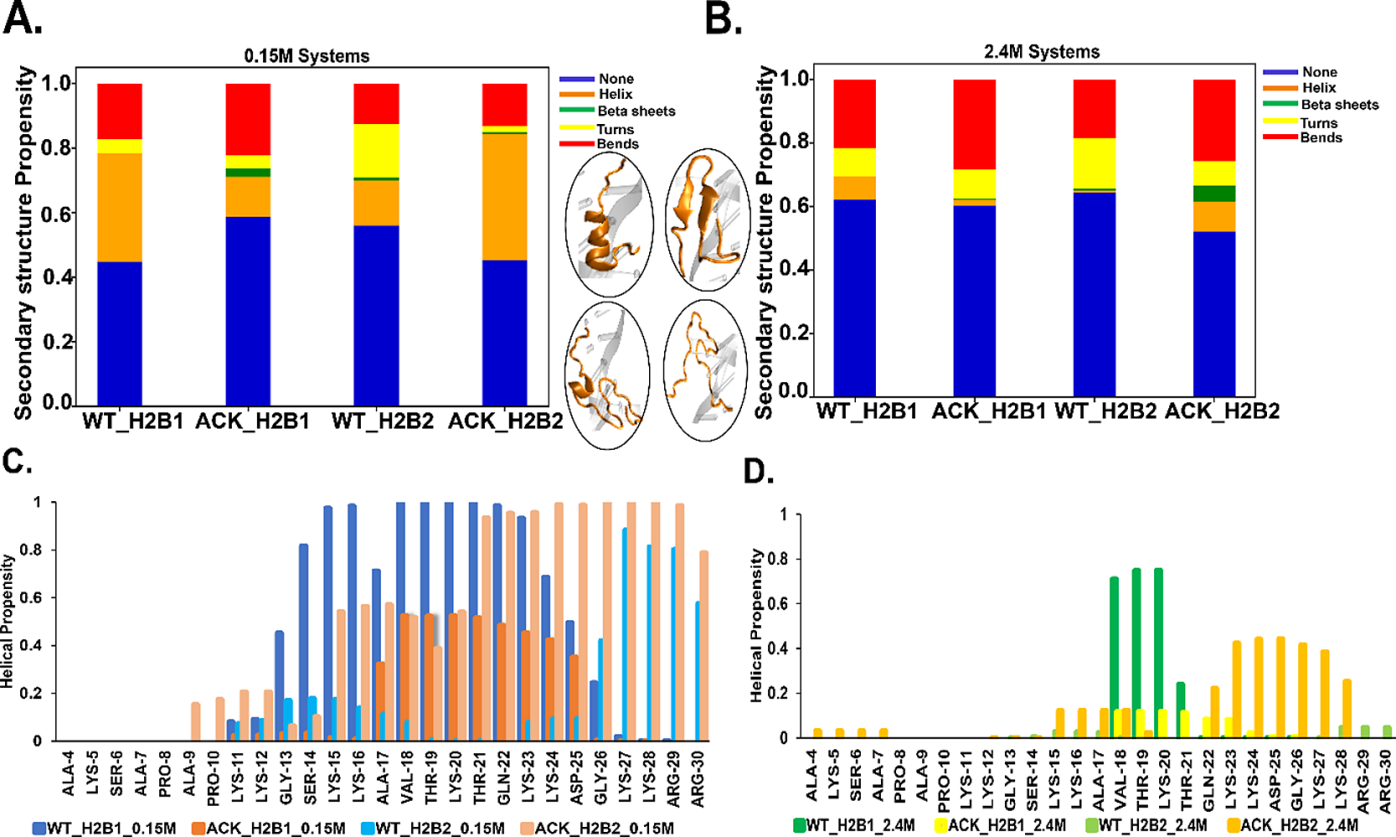
Secondary structure propensity of the H2B N-terminal tails. (A and B) Secondary structure formation of WT and ACK H2B N-terminal tails at 0.15 and 2.4 M NaCl concentrations are represented by different colors: none (blue), helix (orange), β sheets (green), turns (yellow), and bends (red). At 0.15 M NaCl concentration, H2B tail-1 (H2B1) for WT (WT_H2B1) shows more helices. ACK (ACK_H2B1) shows the formation of β-sheets. For H2B tail 2, the helical structures increase in ACK_H2B2 tail, which indicates the compactness of the tail compared to WT_H2B2. At 2.4 M NaCl concentration, the WT_H2B1 tail shows slightly more helices than ACK_H2B1. ACK _H2B2 shows increases in helices compared to WT_H2B2. The four inserts of acetylated H2B tails are shown as an example of distinct secondary structure formation. (C and D) Residue-wise helical propensity of the H2B tail residues of WT and ACK systems at 0.15 and 2.4 M NaCl concentration show the formation of helices (alpha, π, 3_10_ helices) during 1 μs simulation. The beginning residues 4–10 are mostly flexible and disordered, while the ending residues from 11 to 30 show some helical structure in WT_0.15 M. The end of the tail residues 11–30 mostly stays between two DNA gyres around SHL ± 5 and SHL ± 3 regions. The 2.4 M NaCl concentration tails show fewer helical structures, which also include the end of the tail residues.

The H2B N-terminal tails are analyzed for secondary structure for both the WT and ACK systems at both salt concentrations (Figure 3A, B). At 0.15 M salt concentration, H2B tail-1 for the WT shows consistent helix formation throughout the simulation between Gly13 and Lys24 residues (Figures 3C and S6A). Upon acetylation, the H2B tail-1 secondary structure is composed of β-sheets and helices. The β-sheet formation occurs during the first 0.2 μs between Ser14 and Lys24, which, after 0.2 μs, switches to helix formation (Figures 3A, C and S6B). The H2B tail-2 for WT shows helix formation that fluctuates throughout the simulation between Gly13 and Thr19 (Figures 3C and S6C). Conversely, when tail-2 is acetylated, the helix formation shifts between Thr21 and Arg29, with an increase in helical propensity compared to WT tail-2 (Figures 3C and S6D). The ACK tail-2 also forms helices between Ala9 and ACK20. The secondary structure fluctuates between helix, bends, and turns (Figure S6D). At 2.4 M salt concentration, H2B tail-1 for WT shows a helix between Ala17 and Gln22 that fluctuates throughout the simulation (Figures 3B, D, and S7A). This is consistent with our previous reported results^34^ at high salt concentrations, in which helix formation occurs coincident with the formation of a loop near the SHL-5 region of the DNA. In contrast, acetylated tail-1 increases helicity from tail residues Val18 to Lys24 for approximately 0.3–0.4 μs (Figures 3B, D, and S7B). For H2B tail-2, WT shows flickering helical structures between Lys24 and Arg30 residues (Figures 3B, D, and S7C), mostly showing turns and bends. In contrast, acetylation shows a consistent increase in helical structures between ACK15 and Lys28 residues with minor fluctuations (Figures 3B, D, and S6D). Previous experimental studies have predicted the α-helix structure of the H2B tail between residues 10 and 21.^9^ Here our helical propensity data show helix formation mostly between residues 13 and 24 of the H2B tail (Figures 3, S6, and S7). Fu et al.^94^ also indicated that acetylation increases the helical structure of the tail. This agrees with our findings, as our acetylated H2B tails show an increase in the helical structure. Also, at 2.4 M salt concentration, the WT tails show less ordered helical structures than the 0.15 M salt. Previous studies suggested that higher salt concentrations than physiological salt preclude the ability of the N-terminal tails to form more ordered structures.^9^

The secondary structure propensity is also analyzed for simulation replica for both WT and ACK H2B tails at both salt concentrations (Figure S8A,B). The secondary structure propensity is analyzed for all other histone tails at both salt concentrations (Figure S9A–D). Notably, the H3 tails have the highest helix formation, which is consistent with previous results by Potoyan and Papoian.^13^

Indeed, the H3 tails show consistent helix formation for both tails at 0.15 M salt concentration throughout the simulation between Arg2 and Gly12 (Figure S10A,B). The helix formation is also present between Ala21 and Ala29 for both tails, but it flickers for tail-2, indicating that tail-1 overall has more helical formation (Figure S9A). A previous study by Liu and Duan^95^ showed that the WT H3 tail forms α-helical structures based on replica exchange molecular dynamics simulations.^13,95^ This is also consistent with our recently reported long-time simulations at high salt concentration,^52^ which demonstrates a collapse of the full H3 tail. At 2.4 M salt concentration, the helical propensity decreases for both H3 tails with more formation of turns and bends (Figures S9A and S10C,D). We note that the H4 tails showed less helical formation than the other tails. Helix formation appears between Gly13 and Arg17, starting from 0.5 μs for tail-2 at 0.15 M salt concentration (Figure S11A,B). Yang and Arya^41^ showed helical regions between Ala15-Lys20 of the H4 tail, and this supports our findings of the helical region for the H4 tail-2 at 0.15 M salt concentration between Gly13-Arg17. At 2.4 M salt concentration, the H4 tail shows minor helices and mostly bends and turns throughout the simulation for both tails (Figures S9B and S11C,D). The H2A tails show slight helical formation for both tails, staying mostly disordered at 0.15 M salt concentration and almost none at 2.4 M salt concentration (Figures S9C and S12A–D). This type of disordered structure of the H2A tail was observed by Potoyan and Papoian,^13^ as well.

The histone H2B tails are composed of highly charged amino acid residues.^8,34^ Of 30 amino acid H2B tail residues, 12 are positively charged lysine and arginine residues. The highly charged tails with positively charged amino acid residues make minimal intratail interactions^8^ within the tail. Due to this electrostatic repulsion of the highly charged WT tails, the distance between charged residues of the tails increases (Figures S13A,C and S14A,C), which results in the elongation of the tails. Lysine acetylation of lysine 5, 12, 15, and 20 residues of the H2B histone tail removes the positive charge of the lysine and replaces that with an acetyl group (−CH_3_–CO) that neutralizes these four lysine residues. As charge neutralization occurs upon acetylation, the electrostatic repulsion between positively charged groups decreases in the tail. The average distance between the charged residues of the acetylated H2B tail decreases as it becomes shorter (Figures S13B,D and S14B,D). In addition, as acetylation adds a bulky acetyl group, the tail becomes more hydrophobic in nature. As a result, the acetylated tails in their secondary structure show an increase in helical structure as the distance between residues becomes shorter, with less charge repulsion than WT. Helical structure formation increases upon acetylation, especially in H2B tail-2 at 0.15 M salt concentration, compared to WT H2B tails and ACK H2B tail-1. When helix formation occurs, the backbone hydrogen bonds are favorable.^96^ Intratail hydrogen bond analysis (Figure S15) shows an increase in the number of intratail hydrogen bonds in acetylated H2B tail-2 at 0.15 M salt concentration as well as acetylated H2B tail-1 and tail-2 at 2.4 M salt concentration. Alanine (Ala) favors helix formation.^96^ During the trajectory analysis, Ala is part of the helix for tails (Figures 3C, D, S6A–D, and S7A–D). Wang et al.^60^ have described that lysine acetylation increases the α-helical content^50,60,97^ of the histone tails by performing circular dichroism (CD) analysis. This supports our findings of increased levels of helix formation upon acetylation in the H2B tails.

### 4.3 Nucleosomal DNA and Histone H2B N-Terminal Tail Interactions

**Figure 4:**
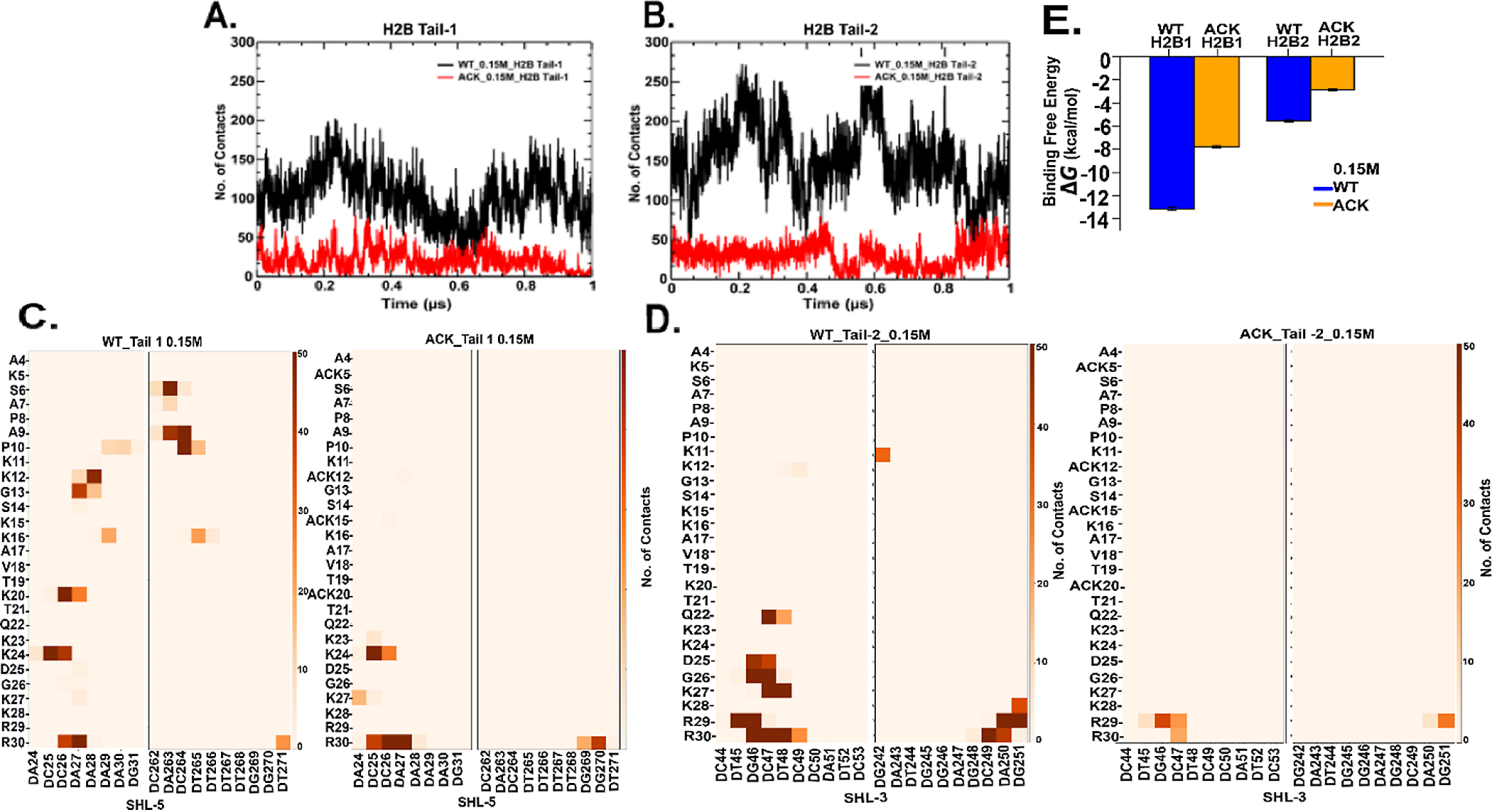
DNA-histone H2B N-terminal tails contacts analysis. (A and B) Number of contacts between H2B N-terminal tail and DNA as a function of time for 0.15 M NaCl concentration over 1 μs simulation with 4.5 Å cutoff distance shows decrease in ACK (red) tails upon acetylating four lysine residues of tails compared to WT (black). (C and D) Contact maps show total number of contacts of WT and ACK tails between specific tail residues and DNA base pair of SHL-5 and SHL-3 for H2B tail-1 and 2 respectively. Overall, contact maps also show a decrease in the number of contacts upon acetylation. (E) Binding free energy calculated for WT (blue) and acetylated (orange) systems for both H2B tails tail-1 (H2B1) and tail-2 (H2B2) with DNA SHL ± 5 regions indicating more binding free energy for the WT system (blue) compared to ACK (orange).

The histone H2B tail is composed of positively charged lysine and arginine residues, which can mainly interact with the negatively charged DNA phosphate backbone through salt bridge formation. Here, we show the total number of contacts between DNA and histone H2B N-terminal tail residues at 0.15 and 2.4 M salt concentrations for both WT and ACK systems (Figures 4, S17, and Table S2–S5). We calculate the number of contacts between the H2B tail residues and DNA with a cutoff distance of 4.5 Å. The number of contacts for the H2B tail decreases upon acetylation compared to the WT (Figures 4A,B and S17A,B). In addition, the number of contacts for WT H2B tail-1 is slightly lower than WT tail-2 for both salt concentrations. The H2B tail protrudes from the histone core between two DNA gyres around SHL ± 5 and SHL ± 3. Therefore, the number of contacts of specific H2B tail residues with specific DNA base pairs from SHL ± 5 and SHL ± 3 have been analyzed (Figures 4C,D and S17C,D). The WT tail-1 residues at both salt concentrations have similar specific DNA contacts, as shown via contact maps. The DNA-tail-1 contacts are distributed throughout the tail from the beginning to the end, mainly consisting of lysine and arginine with the SHL-5 region of the DNA. We note that the DNA sequence CCAAAAG in the SHL-5 region is more prone to interact with H2B tail-1 (Figures 4C, S17C, and Tables S2–S5). For the WT tail-2, most of the DNA-tail residue contacts occur at the end of the tail with residues K11, K12, and Q22-R3. These residues are located between the two gyres close to the SHL-3 region (Figures 4D, S17D, Table S2, and S4). The DNA sequence TGCTCC of the SHL-3 region is more prone to interact with the H2B tail-2. Furthermore, acetylation reduces the number of contacts as per the contact maps in both H2B tails for both 0.15 and 2.4 M salt concentrations (Figures 4C, D, S17C,D, Tables S3, and S5). As acetylation reduces the number of contacts, it also shifts the contacts between DNA and H2B tails-1 and 2 to tail residues from Lys23 to Arg30. Salt-bridge formation between the histone tails and DNA occurs between Lys and Arg residues of the tails and DNA minor and major grooves.^32,98−100^ We have analyzed the number of hydrogen bonds between the DNA phosphate backbone and side chain −NH_3_^+^ group of Lys and guanidium group for arginine (Tables S2–S5). We observe interactions between Lys/Arg side chain atoms and the phosphate groups of specific DNA base pairs. We observe that most of the contacts of H2B tail-1 occur with SHL-5, SHL+4, and SHL+3 regions. Similarly, for H2B tail-2, we observe contacts of H2B tail-2 around SHL+5, SHL-3, and the beginning of SHL+6 closer to SHL+5. When the H2B tails are acetylated, the hydrogen bond interaction shifts further back from the tail residues around Lys23 to Arg30. The back of the H2B tail where Lys and Arg are followed by each other, this part of the H2B tail is more prone to anchor interactions with DNA than other parts of the tail. A previous study by Peng et al.^100^ shows that acetylation reduced contacts between DNA and histone tail and contact regions around SHL regions, including SHL ± 5, SHL+4, and SHL ± 3, consistent with our findings.

In addition, we calculate the binding free energy between histone H2B N-terminal tails and DNA using a molecular mechanics generalized Born surface area (MM/GBSA)^101,102^ approach (Figures 4E and S17E). As the H2B tails mostly interact with the SHL ± 5 regions of the DNA, we calculate the binding free energy between H2B tails and the DNA SHL ± 5 regions for the WT and ACK systems, which show a more negative binding free energy for WT compared to the ACK tails. This is consistent with our results for the number of contacts as the number of contacts reduces upon acetylation in ACK systems compared to that in WT. The binding free energy calculation also provides binding free energy components. The electrostatic energy is the greatest contribution to the binding free energy, which confirms that the interaction between the DNA and histone H2B tails is driven by electrostatic interactions (Figure S18).

The salt bridge formation between DNA and H2B tail also depends on the location of the tail, whether the tail has collapsed onto DNA or is present between the two gyres at the time of contact. The tail becomes transiently exposed to solvent when it is released from DNA. Here, we calculate the solvent-accessible surface area (SASA) of the H2B tails for WT and ACK systems (Figure S16). Usually, if the tail is in contact with DNA, it will be less exposed to the solvent with a lower solvent surface area and the positively charged tail residues make more contact with the negatively charged phosphate groups of the DNA backbone.^34,103,104^ Upon acetylation, the tail is released from DNA and there is a reduction in the number of DNA contacts (Figures 4 and S17). The acetylated tail is exposed to solvent slightly more than the WT as other charged residues in the acetylated tail make it move back to DNA to interact with the negatively charged phosphate backbone. As a result, the SASA can vary throughout the simulation. For both H2B tails, the acetylated tail has slightly higher exposure to the solvent at 0.15 M physiological salt concentration than WT, based on the average values of SASA (Figure S16). At 2.4 M salt concentration, the WT and acetylated tails are exposed almost similarly for tail-1 and slightly higher for WT tail-2. In the compact state, the tail conformation bends away from the solvent but stays in the proximity of the DNA (Figure S16). Here, we show that acetylated lysine tails are exposed to the solvent more often. This may promote recognition of post-translational sites by regulatory proteins.^50^

The favorable interactions between positively charged lysine and arginine residues of the H2B tails interacting with the negatively charged DNA phosphate backbone may dismantle the helical secondary structure of the tails.^103^ The histone tails display secondary structure in both the isolated state and when in the nucleosome complex.^103,104^ We find a higher number of contacts between positively charged tail residues in the WT H2B tail-2 and DNA phosphate backbone (Figures 4B and S17B) at both salt concentrations compared to that of WT tail-1. Moreover, WT tail-2 shows an overall less helical structure compared to WT tail-1 (Figure 3A, B) at a physiological salt concentration of 0.15 M. For both WT H2B tails, 2.4 M salt concentration leads to less secondary structure and more contacts. Hence, this shows agreement with previous studies, as these favorable interactions can interfere with the helical propensity of the tail. Upon acetylation, the number of contacts with DNA decreases for H2B tails at both salt concentrations.

### 4.4 Principal Component Analysis (PCA) of Histone H2B N-Terminal Tails

**Figure 5:**
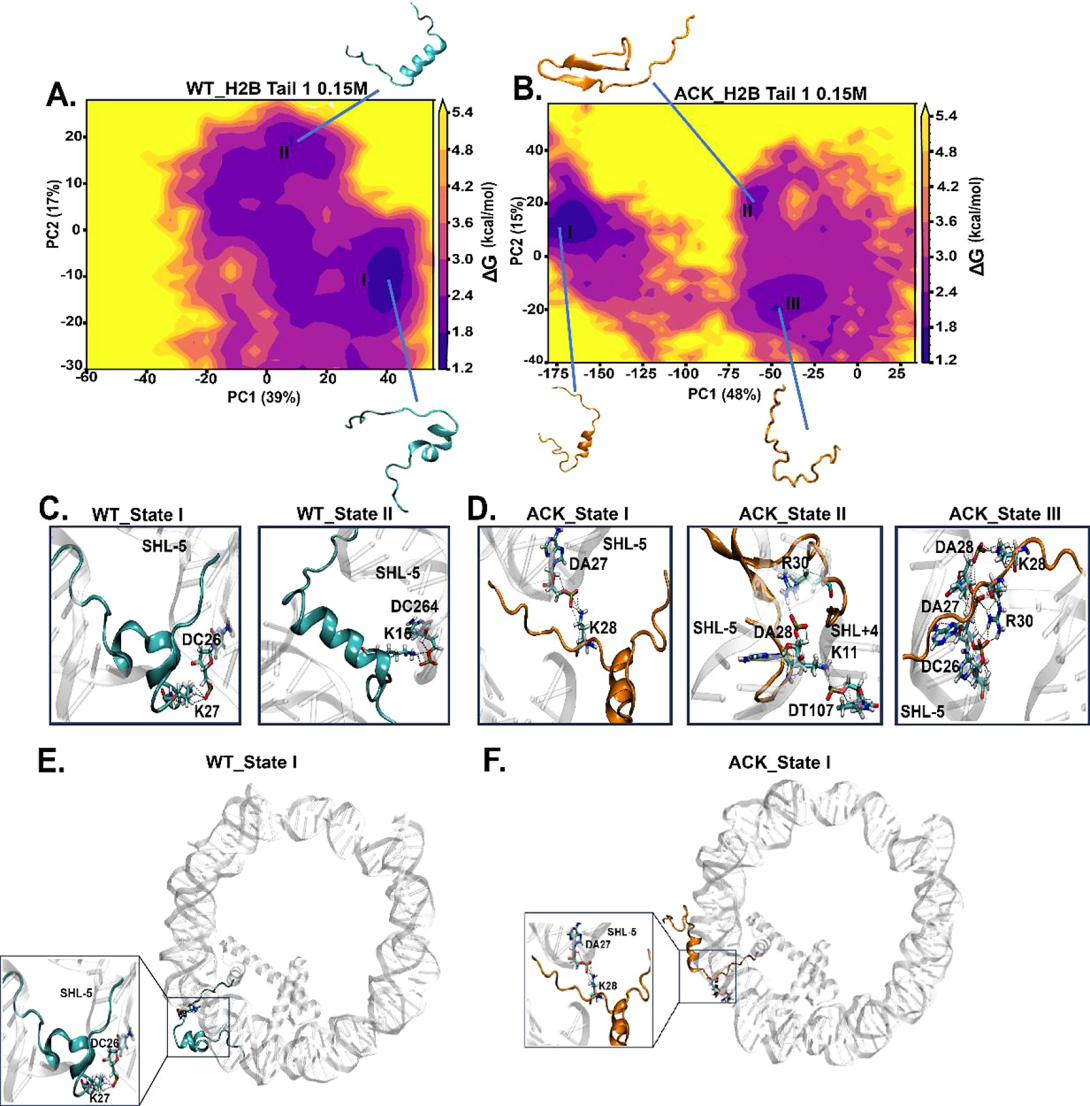
Identification of H2B N-terminal tail-1 conformations upon acetylation using principal component analysis (PCA). (A and B) PCA analysis is performed to study the conformations of the H2B N-terminal tail-1 of WT and ACK for 1 μs simulation. The energy landscape is constructed using the first two principal components (PC) for WT and ACK H2B tail-1 with their percentage variances. (C and D) H2B tail conformations obtained from the PCA free energy surface show tail-DNA interactions between DNA base pairs and positively charged residues of the H2B N-terminal tail. The WT H2B tail-1 states I and II exhibit hydrogen bonds between K27 with the phosphate backbone of DC26 and K15 with DC264 of the SHL-5 region. The ACK H2B tail-1 states I, II, and III exhibit hydrogen bonds among K28, K11, and K28 and DA27, DT107, and DA28, respectively. Also, ACK states II and III exhibit hydrogen bonds between R30 and DA28 and DA27 of the SHL-5 region, respectively. (E) and (F) H2B tail conformational state I of WT and ACK tail’s location with respect to DNA (see Figure S19 for other states).

**Figure 6:**
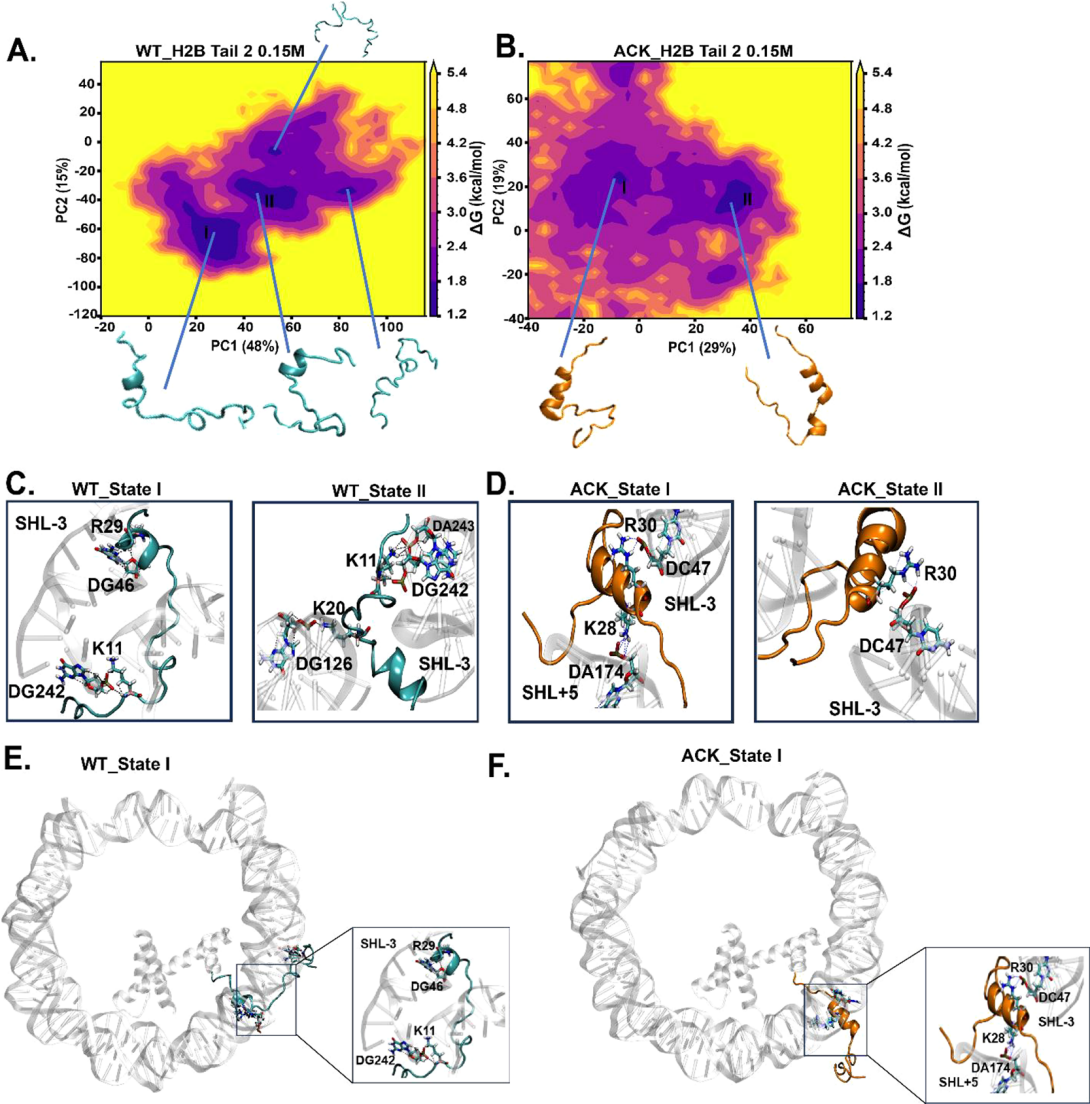
Identification of H2B N-terminal tail-2 dynamics upon acetylation using principal component analysis (PCA). (A and B) PCA analysis is performed to study the conformations of the H2B N-terminal tail-2 of WT and ACK for 1 μs simulation. The energy landscape is constructed using the first two principal components (PC) for the WT and ACK H2B tail-2. It generates more concentrated major minima, to which major tail conformation states belong to. (C and D) H2B tail conformations obtained from the PCA free energy surface show tail–DNA interactions between DNA base pairs and positively charged residues of the H2B N-terminal tail. The WT H2B tail-2 states I and II exhibit hydrogen bonds between K11 and the phosphate backbone of DG242 and between K20 and DG126, respectively. The WT H2B tail-2 state I exhibits a hydrogen bond between R29 and DG46. The ACK H2B tail-2 states I and II exhibit hydrogen bonds between K28 with DA147 and R30 with DC47, respectively. (E and F) H2B tail conformation state I of WT and ACK tail’s location with respect to DNA (see Figure S19 for other states).

**Figure 7:**
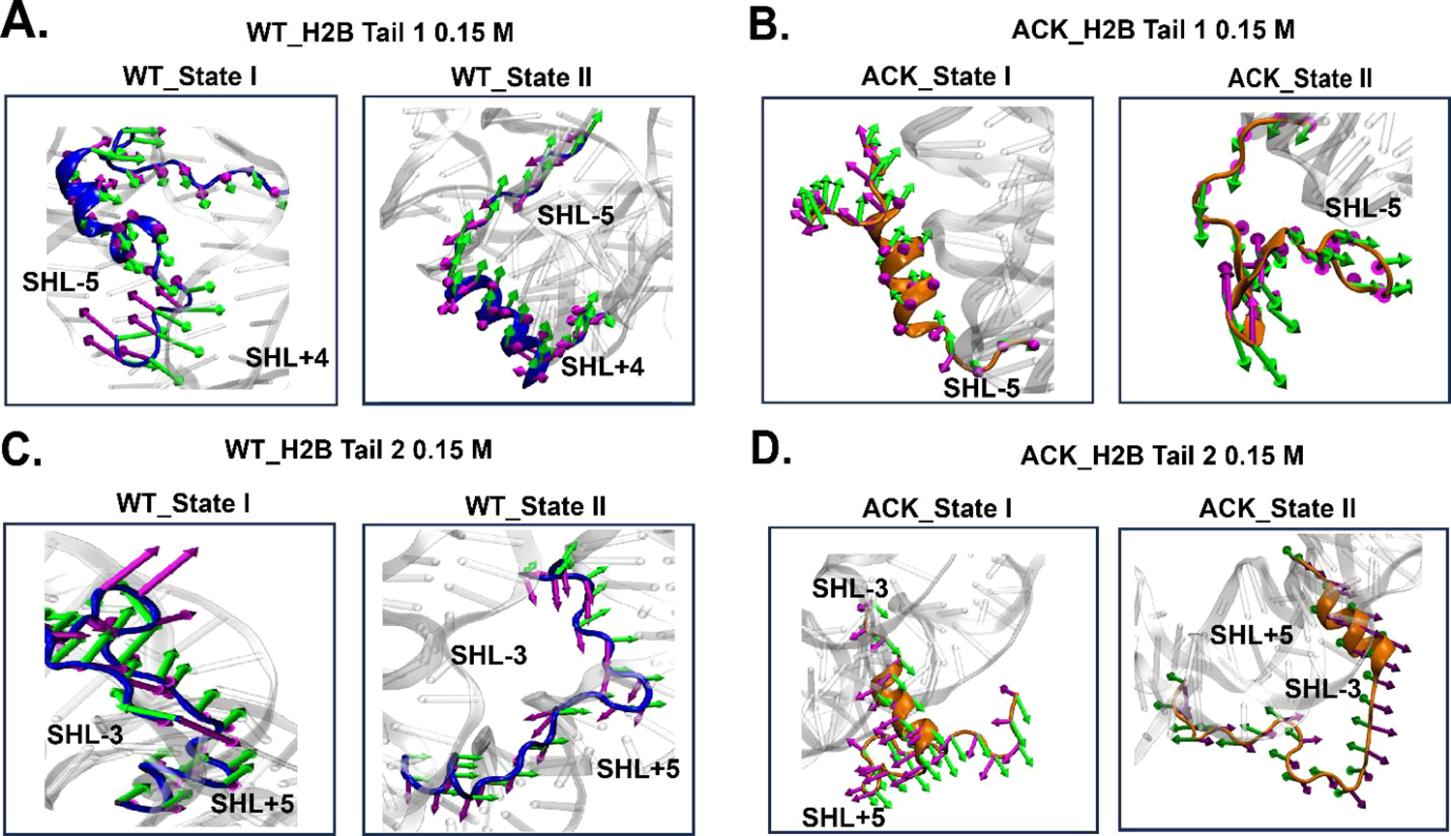
Porcupine plots of H2B tails. Porcupine plots are drawn to visualize the direction of PC1 and PC2 obtained from the PCA. The dominant motions of C_α_ atoms of tail residues in (A) WT H2B tail-1 (blue), (B) ACK H2B tail-1 (orange), (C) WT H2B tail-2 (blue), and (D) ACK H2B tail-2 (orange) are indicated with arrows for each conformation in green (PC1) and magenta (PC2) color. The arrows depict the direction of motion for each conformation. The magnitude of motion is indicated by the length of arrows.

The histone H2B tails are dynamic, with secondary structure rearrangements at microsecond time scales. Here, we analyze major H2B tail conformations using a dimensionality reduction technique known as a principal component analysis (PCA) with the coordinates of the tails. We characterize H2B tail dynamics based on the first two principal components (PCs). The two-dimensional free energy landscape for both H2B tails at both 0.15 M (Figures 5A, B and 6A, B) and 2.4 M (Figure S20A,B and S21A,B) salt concentrations demonstrate that the conformational space of tails is well-defined by distinct basins. Each basin possesses a specific conformation with a distinct secondary structure of the H2B tail. This varies among the different basins. All conformations with different secondary structures have varying helical propensity and number of contacts (Tables S6 and S7). Porcupine plots are constructed to visualize the motion of the H2B tails using the first two eigenvectors for each of the tail conformations of WT and ACK H2B tails at both 0.15 M (Figure 7A–D) and 2.4 M (Figure S23A–D).

For the WT H2B tails at a 0.15 M salt concentration, H2B tail-1 has two distinct conformations (Figure 5A). The helical propensity of state I is 0.26 and 0.23 for state II. On the other hand, the same tail upon acetylation shows a more dispersed free energy landscape with three conformational states. The helical propensity is 0.20 for state I, 0.13 for state II, and none for state III. All conformations agree with our secondary structure analysis for tail-1 (Figure 3A). The cumulative variance percentages of the first 10 eigenvectors are generated and inspected for their contributions to the total fluctuations of H2B tails for all of the systems. PCA for WT H2B tail-1 has indicated that the first five eigenvectors accounted for approximately 80% of the variance in the tail motion observed in the MD simulation. The variance percentages of WT H2B tail-1 of PC1 is 39% and PC2 is 17% as shown in Figure 5A. The acetylated ACK H2B tail-1 shows three distinct conformations including both helical and β-sheet states. The earlier secondary structure analysis confirms these tail conformations (Figures 3A and 5B). PCA for ACK H2B tail-1 has indicated that the first five eigenvectors accounted for approximately 80% of the variance in the tail motion observed in the acetylated MD simulation. The variance percentages of ACK H2B tail-1 of PC1 is 48% and PC2 is 15% shown in Figure 5B. Each tail-1 conformation for WT and ACK is analyzed further for DNA-histone contacts as salt bridge formation can stabilize these conformations (Figure 5C, D, Movies S3, and S4). The number of contacts of tail-1 with DNA for each conformation for 0.15 M salt concentration is shown in Table S6. The conformation of the histone tail for WT and ACK State I is shown in Figure 5E, F. H2B Tail-1 State II tail conformations are shown in Figure S19A,B. As shown earlier, the acetylated tail reduces the total number of contacts compared to that of WT and increases helix structure formation; the acetylated state I of the tail remains in the vicinity of the DNA (Figure 5F).

Similarly, PCA is performed on for H2B tail-2 coordinates at 0.15 M salt concentration, providing major basins for WT and ACK (Figure 6A, B). The corresponding tail conformations with DNA interactions are shown in Figure 6C, D. The helix propensity and number of contacts for these conformations are shown in Table S6. Earlier, we mentioned that favorable DNA-histone interactions disrupt helix formation. WT H2B tail-2, having a less helical structure, provides a higher number of contacts than WT tail-1 (Figures 3A and 4A, B). This pattern holds even when the tail conformation of WT tail-2 is assessed for helical propensity and number of contacts (Table S6); it provides a higher number of contacts with lower helical propensity compared to that of WT tail-1. The state I conformation for WT H2B tail-2 showed less helix propensity than H2B tail-1. Still, the number of contacts is higher due to the tail-2 conformation state I as it collapses upward onto DNA, making it more susceptible to DNA-tail interactions (Figure 6E). PCA for WT H2B tail-2 indicates the first five eigenvectors accounted for approximately 80% of the variance in the tail motion observed in the MD simulation. The variance percentages of WT H2B tail-2 of PC1 is 48% and PC2 is 15%, shown in Figure 6A. For ACK H2B tail-2, the state I conformation stays between two DNA gyres with helix propensity and fewer contacts. This holds a similar pattern as earlier we have observed that ACK H2B tail-2 shows an increase in helical structure and reduced number of contacts compared to WT (Figures 3A and 4A, B). PCA for ACK H2B tail-2 indicates that the first five eigenvectors accounted for approximately 80% of the variance in the tail motion observed in the MD simulation. The variance percentages of ACK H2B tail-2 of PC1 is 29% and PC2 is 19%, as shown in Figure 6B. The conformation state I of ACK H2B tail-2 shows the release of the front of the tail from DNA with the back of the tail between DNA gyres. It interacts with DNA as a helix (Figure 6F). All other H2B tail conformations and their positioning with respect to the DNA are shown in Figure S19A–D.

Porcupine plots are constructed to visualize the H2B tail motion of WT and ACK systems at both 0.15 and 2.4 M salt concentrations (Figures 7 and S23). The WT H2B tail-1 conformations at 0.15 M salt concentration (Figure 7A) show tail movements based on the first two eigenvectors. The first eigenvector (green) of WT H2B tail-1 shows the movement of the tail towards the SHL+4 region, and the second eigenvector (magenta) shows the movement of the tail towards the SHL-5 region, which indicates that the tail motion occurs between these two regions. Also, the length of the arrow, which represents the magnitude of the tail motion shows for WT conformation state I, the front end of the tail is floppy and has higher fluctuations. The ACK H2B tail-1 at conformations 0.15 M salt concentration also shows tail movements based on the first two eigenvectors (Figure 7B). The ACK H2B tail-1 conformations show movement away from DNA with some part of the tail bending toward SHL-5 region of the DNA. In addition, the middle of the ACK H2B tail-1 for conformation state II shows β-sheet formation mostly in the middle of the tail, representing the higher amplitude fluctuation. This shows that β-sheet formation in the middle brings the front end of the tail in the proximity of the SHL-5 region. Further, the WT H2B tail-2 conformations (Figure 7C) at a 0.15 M salt concentration show tail movements based on the first two eigenvectors. The first eigenvector (green) of WT H2B tail-2 shows movement of the tail toward the SHL-3 (Movie S5 WT) region with some end tail residues moving toward the SHL+5 region of the DNA, which indicates that the tail motion occurs between these two regions. The ACK H2B tail-2 (Figure 7D) conformations at 0.15 M salt concentration show movement away from the DNA, with most of the residues showing movement directions toward the SHL-3 region. The ACK H2B tail-2 fluctuations show the direction of the movement away from the DNA.

Similarly, we performed PCA for the H2B tails at 2.4 M salt concentration. Earlier, we showed that at the 2.4 M salt concentration, the tail has more contacts than at the 0.15 M salt concentration, with lower helical propensity. The WT H2B tails at 2.4 M salt concentration show conformations with less helical propensity than those at physiological 0.15 M salt concentration (Figures S20, S21, and Table S7). The WT H2B tail-1 conformation I, with a higher number of contacts, shows the collapse of the tail onto the DNA; whereas the ACK tail-1 conformation state I shows the slight helical structure of the tail, with the front residues (residues 4–15) of the tail collapsing onto DNA with a lower number of contacts (Figure S20A,B,E,F and Table S7). The tail-1 conformations interact with specific DNA base pairs in the SHL-5 region (Figure S20C,D). PCA for WT H2B tail-1 indicates the first five eigenvectors account for approximately 80% of the variance in the tail motion observed in the MD simulation. The variance percentages of WT H2B tail-1 of PC1 is 41% and PC2 is 17% (Figure S20A). Similarly, PCA for ACK H2B tail-1 indicates that the first five eigenvectors account for approximately 80% of the variance in the tail motion observed in the MD simulation. The variance percentages of ACK H2B tail-1 of PC1 is 48% and PC2 is 12% (Figure S20B). Furthermore, the PCA of H2B tail-2 at 2.4 M salt concentration also shows major basins to which the conformations belong (Figure S21A–D) for both the WT and ACK systems. PCA for WT H2B tail-2 indicates the first five eigenvectors account for approximately 80% of the variance in the tail motion observed in the MD simulation. The variance percentages of WT H2B tail-2 of PC1 is 35% and PC2 is 22% (Figure S21A). Similarly, PCA for ACK H2B tail-2 indicates that the first five eigenvectors account for approximately 80% of the variance in the tail motion observed in the MD simulation. The variance percentages of ACK H2B tail-2 of PC1 is 30% and PC2 is 26% (Figure S21). The tail conformations with respect to the DNA are also shown in Figure S22.

The WT H2B tail-1 conformations at 2.4 M salt concentration (Figure S23A) show direction of tail motion based on the first two eigenvectors. The first eigenvector (blue) of WT H2B tail-1 shows movement of the tail toward the SHL+4 region, and the second eigenvector (magenta) shows movement of the tail toward the SHL-5 region, which indicates that the tail motion occurs between these two regions. Also, the length of the arrow, which represents the magnitude of the tail motion, shows that for WT conformation state II, the front end of the tail is floppy and has more fluctuations. The ACK H2B tail-1 at conformations 2.4 M salt concentration also shows tail direction of motion based on the first two eigenvectors (Figure S23B). The ACK H2B tail-1 conformations show movement away from DNA, with the back end of the tail bending toward the SHL-5 region of the DNA upon slight helix formation. Further, the WT H2B tail-2 conformations (Figure S23C) at 2.4 M salt concentration show tail movements based on the first two eigenvectors. The first eigenvector (green) of WT H2B tail-2 shows movement of the tail toward the SHL-3 region with some end of tail residues moving toward the SHL+5 region of the DNA, which indicates that the tail motion occurs between these two regions. The ACK H2B tail-2 (Figure 23D) conformations at 2.4 M salt concentration show movements away from the DNA, with most residues moving toward the SHL-3 region. The ACK H2B tail-2 fluctuations mostly show the direction of the movement away from most of the tail residues for the second eigenvector for conformation state I.

Overall, the conformations from PCA support our secondary structure analysis and the number of DNA-H2B tail contacts. All PCA conformational states and their location with respect to the nucleosomal DNA point to partial helical or no helical structure, keeping the tail in the vicinity of the DNA or bringing the nonhelical part of the tail toward DNA for favorable DNA-histone interactions. A previous study by Wang et al.^60^ has addressed that increasing helical propensity is independent of DNA-histone tail interactions. Thus, it supports our findings when we observe an increase in helical propensity with fewer contacts for tail-1 compared to tail-2. This also supports the behavior of the acetylated tail, as most of the acetylated tails show the formation of helical structures but with a lower number of DNA-H2B tail contacts.

## 5 Discussion

Nucleosome core particles (NCPs) are fundamental units of chromatin and play a direct role in gene regulation. NCPs are composed of a histone octamer (H3, H4, H2A, H2B) with ∼147 bp of DNA wrapped around them. Each histone contains N-terminal tails that are major sites for the PTMs. In this study, we aim to elucidate the conformational dynamics of histone H2B tails upon acetylation that can provide insight into their contribution to biological functions. For this purpose, we have performed 1 μs long all-atomistic MD simulations of four NCP systems (PDB: 1KX5) including 0.15 M salt concentration of unacetylated (WT_0.15M) and lysine-acetylated (ACK_0.15M) H2B tails as well as 2.4 M salt concentration of unacetylated (WT_2.4M) and 2.4 M lysine-acetylated (ACK_2.4M) H2B tails. The lysine residues that are acetylated for both H2B tails are Lys 5, 12, 15, and 20 for both 0.15 and 2.4 M salt concentrations. We acetylated these particular lysine residues of the H2B tails, as they are associated with the p14ARF^105^ tumor repressor protein and ATF2^106^ coactivator. These proteins maintain transcription function through interaction with the H2B tails at these four Lys 5, 12, 15, and 20 residues through acetylation. The H2B tails are resolved in the 1KX5 NCP structure and have the first three residues missing; however, most of the tail is resolved. Previous studies^94,100^ have used the same 1KX5 structure for their molecular dynamics simulations. In addition, as our study mostly focuses on lysine acetylation, these residues are present and resolved in the 1KX5 structure. For each of the four systems, we analyzed the effects of acetylation on the RMSD, *R*_g_, secondary structure propensity, and DNA–tail contacts of both H2B tails.

Our MD simulations focus uniquely on both H2B tails and consider the acetylation of the same lysine residues in both H2B tails, as both tails are structurally identical. This provides insight into shifts in the structure and dynamics upon acetylation of both tails. We use the amber ff19SB^64^ for the histone and the OL15^65^ force field for the DNA. Previous studies^13,37,62,94,100,107^ of the NCP have used ff14SB^108^ and ff99SB^109^ force fields to simulate the NCP complex with the N-terminal tails. The ff19SB is an improved force field from the previous ff14SB; it is shown to be better with globular proteins. In future work, we can explore the force field for disordered proteins by Robustelli et al.,^67^ known as a99SB-disp which can better simulate both ordered and disordered protein states. An appropriate force field for disordered proteins can be challenging. Some previous studies^110−112^ have compared force fields for disordered proteins. The study by Potoyan and Papoian^13^ used the original and modified ff99SB for the isolated histone N-terminal tails. The ff19SB force field could be improved to better simulate disordered proteins; however, we expect the trends between acetylated and nonacetylated tails to hold if the force field is improved.

A summary of the analysis of both H2B tails is shown in Table 1 for acetylation at a 0.15 M salt concentration. The radius of gyration (*R*_g_) and RMSD for acetylated H2B tails are higher than those for WT for both tails at 0.15 M salt concentration. Our secondary structure analysis indicates that the H2B tails undergo structural rearrangements, increasing helix and β-sheet formation at both salt concentrations. The secondary structure analysis shows similar patterns in simulation replicas, with a slight increase of helices observed for acetylated H2B tail-2 in the replicas. For the other histone tails, H3, H4, and H2A, the radius of gyration of the replicas at both salt concentrations shows very slight differences in the probability distribution. Here, we report microsecond time scale simulations of the 1KX5 system at both physiological 0.15 and 2.4 M salt concentrations show that WT H2B tail-2 exhibits a higher number of contacts with DNA than tail-1. This is consistent with the findings we reported in Khatua et al.^52^ which were performed at 2.4 M salt concentration to sample metastable states along the unwrapping pathway. Our results indicate that lysine charge neutralization upon acetylation reduces the number of contacts between the DNA and H2B N-terminal tails as the tail is released from the DNA surface but remains in the vicinity of DNA. Also, acetylation reduces the charge repulsion within the tail and increases tail compaction due to the addition of the acetyl group on the lysine residues, increasing the hydrophobicity of the tail.

**Table 1:** Summary of the Effects of Lysine Acetylation in the H2B N-Terminal Tails That Influence Tail Secondary Structure, Contacts, and Binding Free Energy

analysis	effects on H2B N-terminal tails upon acetylation
radius of gyration (*R*_g_)	with acetylation, the *R*_g_ of the H2B tails compared to WT increases
secondary structure propensity	with acetylation, tail-1 helix and β-sheet propensity increases; the H2B tail-2 helix propensity also increases
DNA–tail contacts	number of contacts reduces upon acetylation compared to WT for both H2B tails
binding free energy	binding free energy weakens upon acetylation compared to WT for both H2B tails

Our results are consistent with previous computational studies of the effects of acetylation on tail structure and DNA–protein contacts. Previous studies of H4 tail acetylation by Shabane et al.^107^ show that mutation of lysine to glutamine (K → Q) and the progressive acetylation of the tail to explore PTM effects results in tail compaction and secondary structural rearrangements. They investigated structural ensembles of the H4 histone tail and its various states of lysine acetylation and acetylation mimics under physiological conditions. The charge neutralization of the H4 tail affected the binding of the tail to the neighboring nucleosome acidic patch. As the tail becomes significantly more compact, the progressive acetylation of the H4 tail increases DNA accessibility. They suggest that H4K16, known to interact with the acidic patch of the neighboring nucleosome, would be influenced upon acetylation. By neutralizing the charge of H4K16, it would disrupt the neighboring nucleosome interaction as the tail compacts; however, it might favor interacting with its own DNA. In addition, progressive charge neutralization of the H4 tail would lead to chromatin decondensation, increasing DNA accessibility to various transcription factors.^107^ Similarly, we observe a decrease in the number of contacts between the H2B tail and DNA upon charge neutralization; therefore, the DNA might be more accessible to other proteins to conduct biological processes.

Furthermore, we observe that the number of contacts between the DNA and H2B tail occurs mostly around the SHL+4, SHL-5 regions for H2B tail-1 and SHL-3, and SHL+5 for H2B tail-2 for both salt concentrations. Upon acetylation of these H2B tails, the tails are usually released from the DNA but remain in the vicinity of the DNA surface, reducing the number of contacts between the DNA and the H2B tail. As calculated with MM/GBSA, the binding free energy between the DNA and H2B tails for WT systems at both salt concentrations is more favorable for the WT than for the ACK systems. Previous studies by Peng et al.^100^ also show a reduced number and redistribution of contacts upon acetylation, which is consistent with our results. At both salt concentrations, we show specific contacts between DNA base pairs and H2B tail residues for the WT and ACK systems. We observe that as acetylation reduces the number of contacts, it shifts the contacts toward the back of the H2B tail sequence where the majority of lysines and arginines are present. The reduced number of contacts makes DNA accessible for other nucleosome-binding proteins, which specifically recognize certain histone tail sites and DNA.

While H2B has been less well characterized than the other histone tails, specific lysines in the H2B tails play key roles in the interaction with p14ARF tumor suppressor protein and also transcription factors, such as ATF2, activating transcription factor 2. The interaction of p14ARF with the H2B tails involves deacetylation via HDAC1, which leads to transcription repression, and upon the dissociation of p14ARF, HAT acetylates the H2B tails to active transcription. The specific H2B N-terminal lysine (Lys, K) residues involved in this process are K5, K12, K15, and K20.^105^ Further, the p14ARF tumor repressor protein controls apoptosis or cell death due to oncogenic stress and regulates gene transcription. It has been found that p14ARF is mutated in many types of human cancers. It has been shown that p14ARF maintains its repressive transcription function through interaction with the H2B tails. Also, ATF2 (activating transcription factor 2) has been associated with H2B and H4 acetylation. Studies have shown that ATF2 is a coactivator for p300 HAT, but ATF2 also has HAT activity. ATF2 acetylates lysine residues in the H2B tail at K5, K12, and K15 positions, which causes transcription activation.^106^ One of the previous MD simulation studies by Fu et al.^94^ characterizes the effect of lesions within the NCP for DNA repair. This study has shown that by acetylating all lysine residues of only one of the H2B tails, the lesion’s partial entrapment of the tail would disrupt the binding of other regulatory proteins, destroying tail-governed processes such as transcription and DNA repair. The comparable dynamics of both H2B tails under physiological conditions without lesions remains to be studied. Thus, the H2B N-terminal tail is a critical regulator in nucleosome stability and gene expression, yet it is poorly understood.

Previous studies have shown that the removal of the H2B tail can promote nucleosome sliding due to the disruption of DNA–histone tail interactions. Similarly, as the H2B tail contacts DNA around the SHL ± 5 region, acetylation of the tail reduces the number of contacts with the DNA. This may lead to nucleosome sliding, as it could act in a similar way as removing the histone tail. In addition, histone acetylation may aid in nucleosome displacement in the presence of Swi/Snf (SWItch/Sucrose Non-Fermentable) chromatin remodeling complexes.^113,114^ We observe compaction of the H2B tail upon acetylation. Compaction is also observed in previous studies. As mentioned earlier, the compact tail conformation could serve as a docking site for other proteins that recognize acetylated sites. Bromodomains (BRDs) are protein interaction modules that recognize multiple acetylated lysine sites for a single BRD for a tight interaction with histone tails.^45,115^ As shown in our study, multiple lysine-acetylated residues can shorten the distance within the intratail residues as acetylation reduces charge repulsion, which may aid in binding to the deep hydrophobic binding pockets of BRD. Our selected Lys 5, 12, 15, and 20 are residues that show relatively strong binding to BRD upon acetylation.^81^ BRDs are associated with epigenetic reader modules and have been implicated in drug development for the treatment of diseases such as cancer.^115−118^ Furthermore, experimental studies such as single-molecule FRET have characterized the conformations and dynamics of H3 at different salt concentrations.^119^ Here, we characterize the conformations of the H2B tails for both the WT and the ACK systems by principal component analysis (PCA). The charge neutralization of histone H2B tail residues suppresses contacts between the DNA and histone tail, enhancing the tail dynamics and DNA accessibility. The conformations of the histone H2B tail obtained from PCA provide insight into key tail residues stabilizing each conformational state of the histone H2B tails.

## 6 Conclusions

In conclusion, we perform several sets of 1 μs long all-atomistic molecular dynamics simulations of the NCP at 0.15 and 2.4 M NaCl concentrations for WT (unacetylated) and ACK (acetylated H2B N-terminal) systems. The four lysine residues Lys 5, Lys12, Lys15, and Lys20 of both H2B tails are acetylated. Our results indicate that lysine charge neutralization upon acetylation reduced the number of DNA-H2B N-terminal tail contacts, making DNA more accessible for other regulatory proteins to bind to carry out biological processes. The acetylated H2B tails can serve as docking sites for other regulatory proteins. The H2B tail is a critical regulator for gene expression and certain diseases; disrupting the deacetylation of these tails could provide therapeutic potential for various diseases. Our secondary structure analysis shows structural rearrangements of the tails upon acetylation. PCA also indicates a change in the conformational space of the tails upon acetylation. The transition between different conformations of tails can be investigated by using Markov State Models (MSMs). MSMs can provide information based on the kinetic exchange between states, which can partition conformational space into metastable regions.^120–123^ As the histone in the NCP contains both an ordered globular core and disordered N-terminal tails, future simulations can compare the effects of acetylation for ff99SB-disp. The f19SB force field could also be improved to better describe both ordered and disordered proteins. Taken together, H2B tail acetylation causes a decrease in the formation of salt bridges with DNA, which can increase the accessibility of DNA for regulatory proteins to bind for biological functions and impact nucleosome plasticity.

## Data Availability

We have used the Amber18 MD simulation package to perform all the simulations. The software can be found at https://ambermd.org/GetAmber.php. The data analysis has been carried out using MDAnalysis. Analysis codes are available at https://github.com/CUNY-CSI-Loverde-Laboratory/H2B_N_terminal_tails.

## Supporting Information Available

Additional analysis of WT and acetylated NCP at both 0.15 and 2.4 M salt concentrations (PDF)
