# Supplementary material for: Conformational Dynamics of the Nucleosomal Histone H2B Tails Revealed by Molecular Dynamics Simulations
Source: J Chem Inf Model. 2024 Jun 12;64(12):4709–26. doi: 10.1021/acs.jcim.4c00059 (PMC11200259; doi:10.1021/acs.jcim.4c00059)
Supplement: Supplementary file 1 — ci4c00059_si_001.pdf [file ci4c00059_si_001.pdf]

Supporting Information

Conformational dynamics of the nucleosomal histone H2B tails revealed by molecular dynamics simulations

Rutika Patel[1,2], Augustine Onyema[1,2], Phu K. Tang[1,2,5], Sharon M. Loverde[1,2,3,4]

[1]Ph.D. Program in Biochemistry, The Graduate Center of the City University of New York, New York, NY, 10016

[2]Department of Chemistry, College of Staten Island, The City University of New York, 2800 Victory Boulevard, Staten Island, New York, 10314, United States.

[3]Ph.D. Program in Chemistry, The Graduate Center of the City University of New York, New York, NY, 10016

[4]Ph.D. Program in Physics, The Graduate Center of the City University of New York, New York, NY, 10016

[5]Present Address: Flatiron Institute, New York, NY

E-mail: sharon.loverde@csi.cuny.edu, Phone: 718-982-4075, Fax: 718-982-3910

Methods

Intra-tail hydrogen bond Analysis

The number of intra-tail hydrogen bonds of the WT and ACK H2B tails residues was determined using AmberTools21 cpptraj[1]. The number of hydrogen bonds for each of the H2B tail residues averaged over the trajectory.

Distance Analysis of H2B Tail Residues

The residue-to-residue distance of the WT and ACK H2B tails was calculated using MDAnalysis[2]. The distance for each of the H2B tail residues was averaged over the trajectory and plotted.

Binding free energy (MM/GBSA)

The binding free energy between histone H2B N-terminal tails and DNA was calculated using molecular mechanics generalized Born surface area (MM/GBSA)[3, 4] method implemented in the Amber22 package. In the MM/GBSA approach, the free energy (ΔG_{bind}) for binding of the DNA as ligand (L) to the H2B tail as protein receptor (R) to form the complex (RL). The free energy equation can be expressed as below:

$$\Delta G_{bind} = G_{RL} - (G_R - G_L) \quad (1)$$

The binding free energy (ΔG_{bind}) can be decomposed into contributions of different interactions and can be written as below:

$$\Delta G_{bind} = \Delta H - \mathrm{T}\Delta \mathrm{S} = \Delta E_{MM} + \Delta G_{sol} - T\Delta S \quad (2)$$

Where,

$$\Delta E_{MM} = \Delta E_{int} + \Delta E_{ele} + \Delta E_{vdW} \quad (3)$$

$$\Delta G_{sol} = \Delta G_{PB/GB} + \Delta G_{SA} \quad (4)$$

ΔE_{MM} is the change in the gas-phase molecular mechanics (MM) energy and includes changes in the internal energies ΔE_{int} (bond, angle, and dihedral energies), electrostatic energies ΔE_{ele}, and the van der Waals energies ΔE_{vdW}. ΔG_{sol} is the solvation energy which includes the run of the electrostatic solvation energy $\Delta G_{PB/GB}$ (polar contribution) and nonpolar contribution ΔG_{SA} between the solute and the continuum solvent. $-T\Delta S$ is conformational entropy upon ligad binding[4]. Here, the binding free energy was calculated between the DNA SHL±5 region and H2B N-terminal tail residues as the H2B tail interacts with DNA around the SHL±5 region. The calculations were performed using the MM/GBSA tool in AMBER tools based which uses default buffer as 14 angstroms.

Radial Distribution Function

The radial distribution function, g(r), indicates the probability of finding an atom at a given distance from a reference atom. g(r) shows the time-averaged density of particles in (b) from the reference group (a) at a

distance (r), as shown in the equation $g_{ab}(r) = (N_aN_b)^{-1}\sum_{i=1}^{Na}\sum_{j=0}^{Nb}\langle\delta(\,|ri - rj| - r)\rangle$. The g(r) for ions ($Na^+$) around the DNA SHL±5 region and Cl^- ions around the H2B tails is calculated using MDAnalysis[2].

Figures

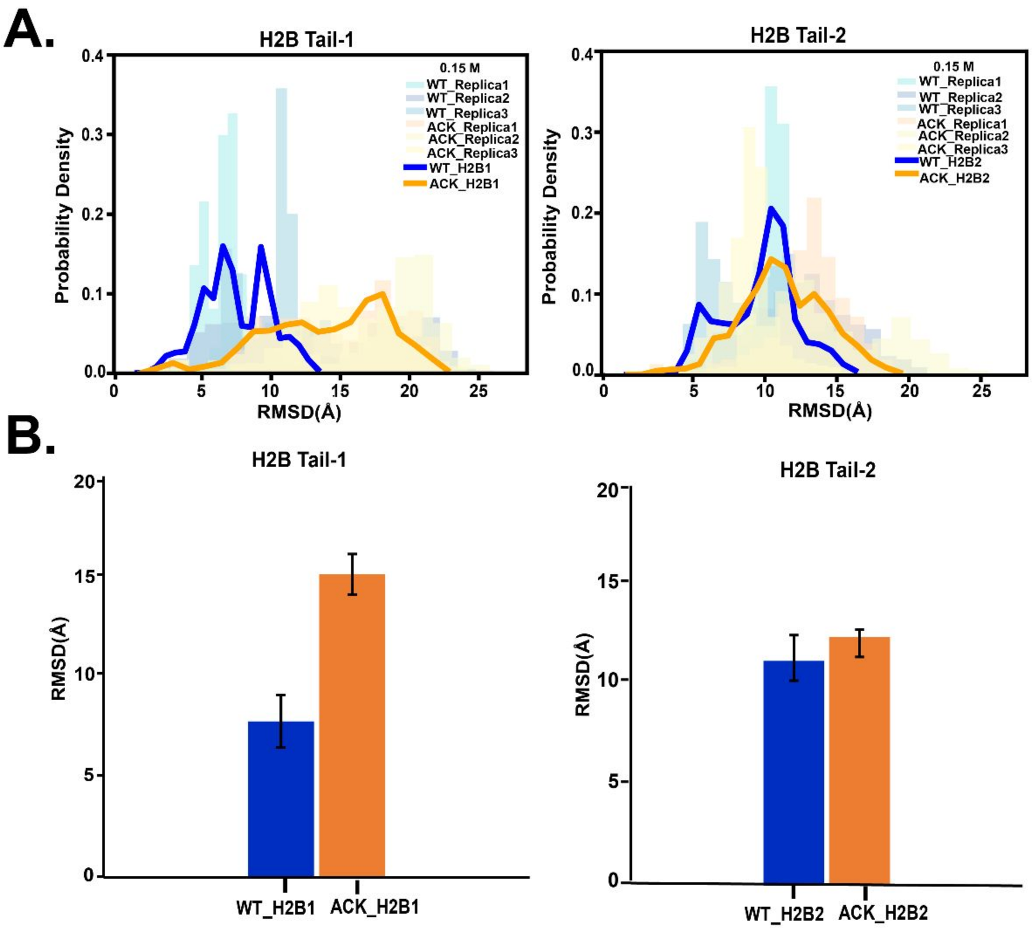

Figure S1. RMSD analysis of H2B N-terminal tail upon acetylation. (A) The Root-Mean-Square-Deviations (RMSDs) of H2B N-terminal tails at 0.15 M NaCl concentration are calculated based on C_α of the tail residues over 1 µs simulation with respect to initial structure. The H2B Tail-1 (H2B1) probability density distribution of the ACK tail (orange) is more extended compared to WT (blue). The H2B Tail-2 (H2B2) probability density distribution shows a slightly extended tail upon acetylation (orange) compared to WT (blue). The histogram shows the distribution for three replicas and the solid line represents the average of RMSD for replicas. **(B)** The block average RMSD of three replicas for H2B tails for WT and ACK systems at 0.15 M salt concentrations is obtained by dividing the data into non-overlapping blocks using 11 blocks approximately 91 ns per block for 1 µs simulation.

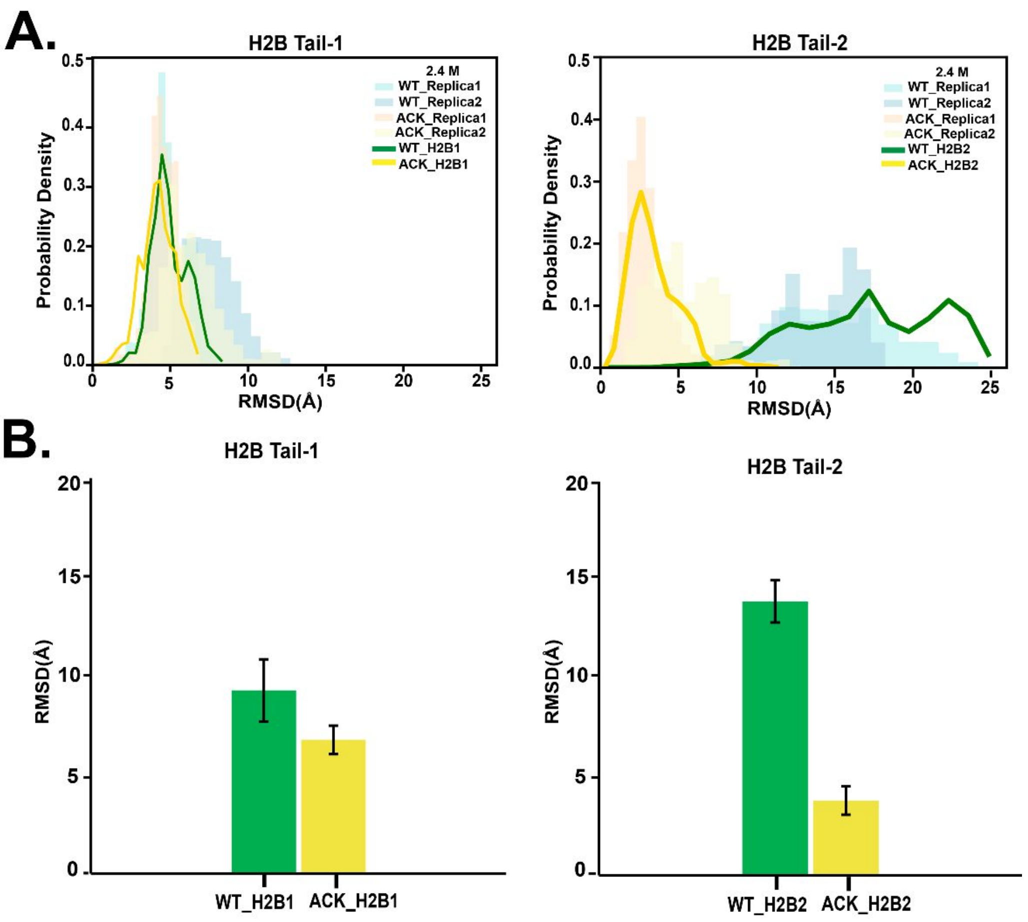

Figure S2. RMSD analysis of H2B N-terminal tail upon acetylation. (A) The Root-Mean-Square-Deviations (RMSDs) of H2B N-terminal tails at 2.4 M NaCl concentration are calculated based on C_{α} of the tail residues over 1 μs simulation with respect to initial structure. The H2B Tail-1 (H2B1) probability density distribution of the ACK tail (yellow) is more extended compared to WT (green). The H2B Tail-2 (H2B2) probability density distribution shows a slightly extended tail upon acetylation (yellow) compared to WT (green). The histogram shows the distribution for two replicas and the solid line represents the average of RMSD for both replicas. **(B)** The average RMSD of two replicas for H2B tails for WT and ACK systems at 2.4 M salt concentrations is obtained by dividing the data into non-overlapping blocks using 11 blocks approximately 91 ns per block for 1 μs simulation.

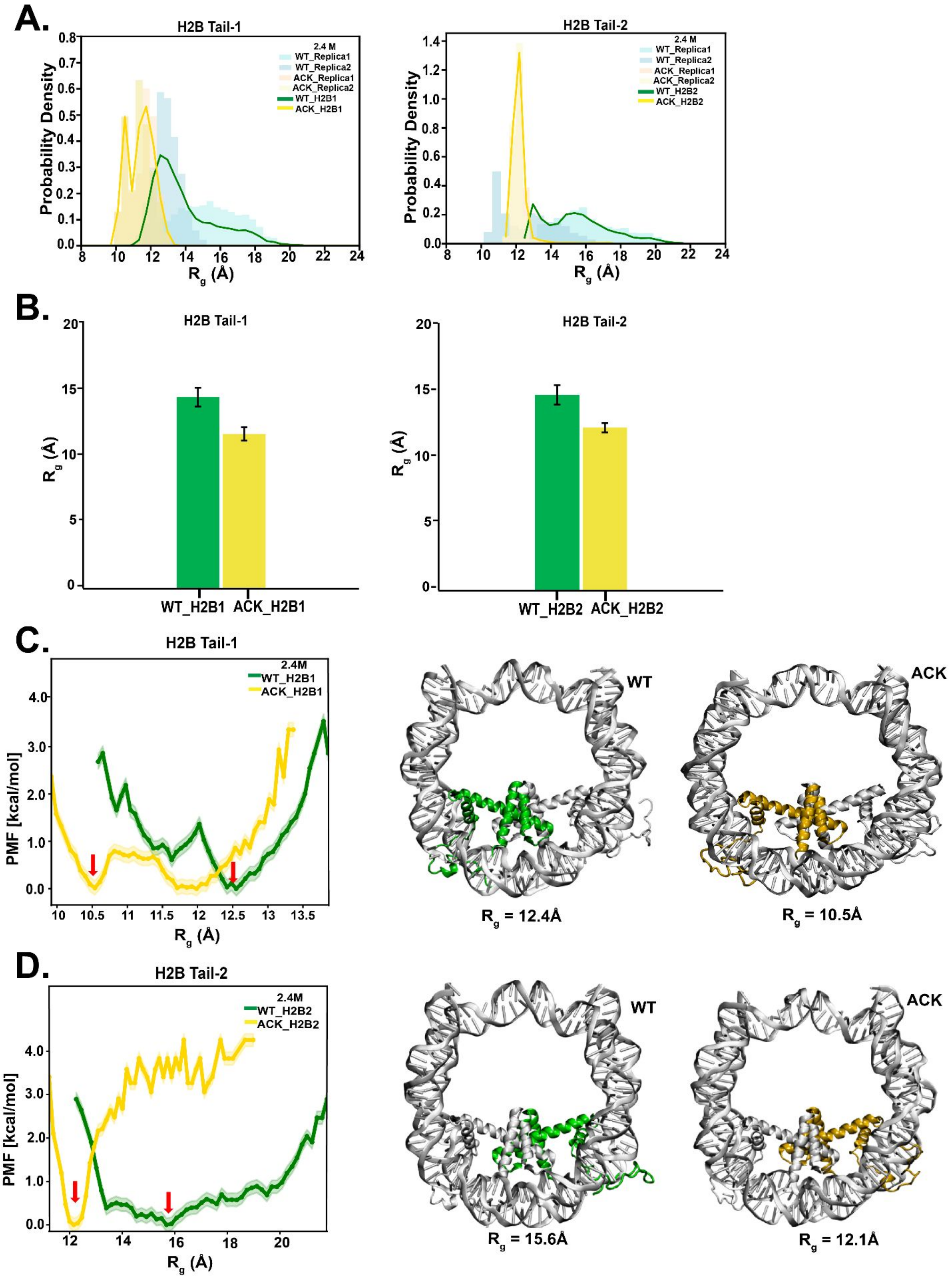
A.
H2B Tail-1
2.4 M
WT_Replica1
WT_Replica2
ACK_Replica1
ACK_Replica2
WT_H2B1
ACK_H2B1
Probability Density
R_g (Å)
H2B Tail-2
WT_H2B2
ACK_H2B2
B.
WT_H2B1
ACK_H2B1
WT_H2B2
ACK_H2B2
C.
2.4M
PMF [kcal/mol]
WT
ACK
R_g = 12.4Å
R_g = 10.5Å
D.
R_g = 15.6Å
R_g = 12.1Å

90

Figure S3. Radius of gyration (R_g) of H2B N-terminal tail upon acetylation. (A) The radius of gyration (R_g) of H2B N-terminal tails at 2.4 M NaCl concentration are calculated based on C_α of the tail residues over 1 µs simulation. The H2B Tail-1 (H2B1) probability density distribution of the ACK tail (yellow) is extended compared to WT (green). The H2B Tail-2 (H2B2) probability density distribution shows a slightly extended tail upon acetylation (yellow) compared to WT (green). The histogram shows the distribution for two replicas and the solid line represents the average of R_g for both replicas. **(B)** The average R_g of two replicas for H2B tails for WT and ACK systems at 2.4 M salt concentrations is obtained by dividing the data into non-overlapping blocks using 11 blocks approximately 91 ns per block for 1 µs simulation. **(C) and (D)** The potential of mean force (PMF) as a function of the radius of gyration (R_g) for both H2B Tail-1 and Tail-2 for WT (green) and ACK (yellow) is calculated based on PMF = $-K_bT \log (P/P_{max})$. The configurations of H2B tails for both WT (green) and ACK (yellow) systems are shown with their corresponding R_g values.

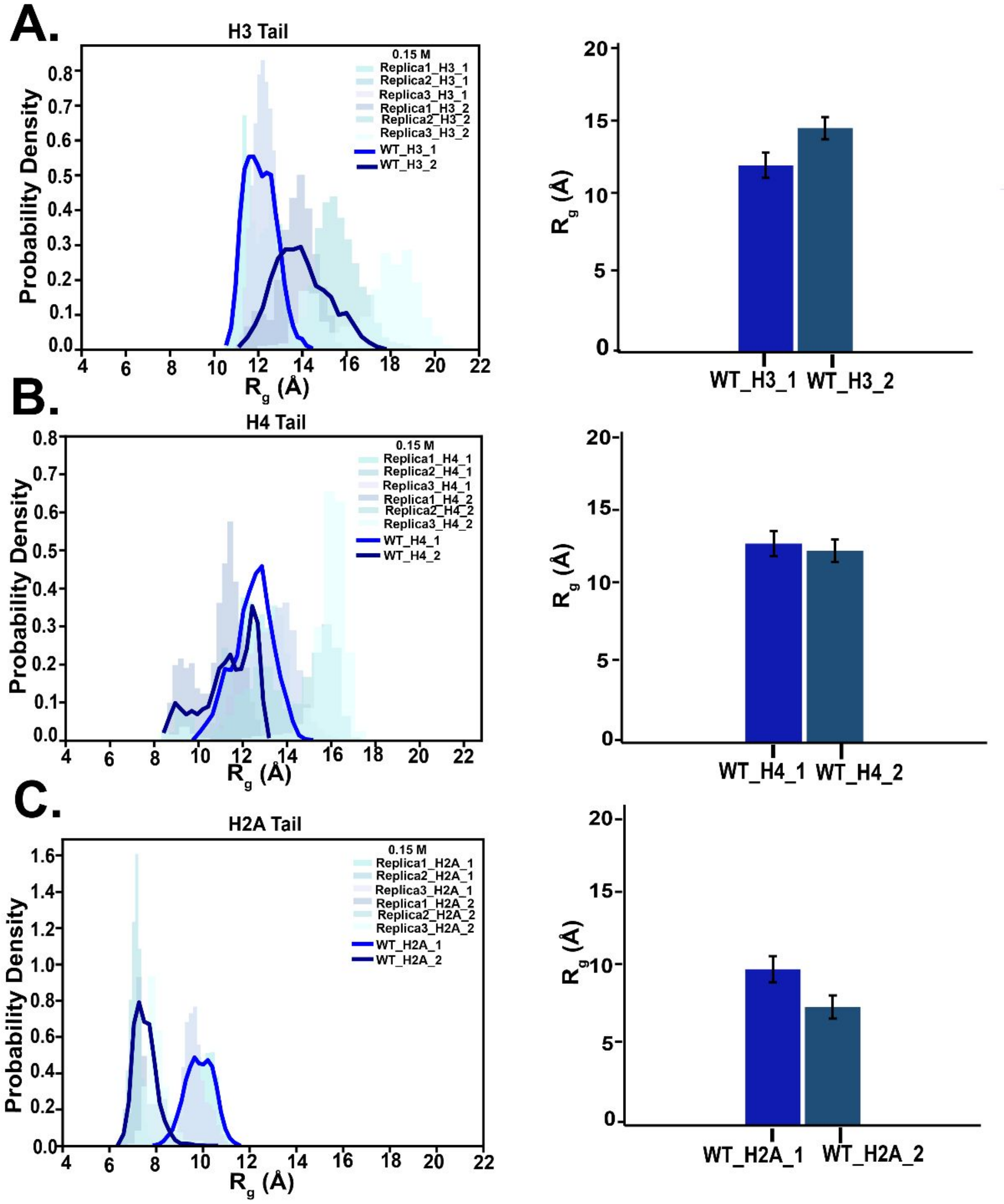

Figure S4. Radius of gyration (R_g) of histone N-terminal tails at 0.15 M salt concentration. (A) The radius of gyration (R_g) of H3 N-terminal tails (B) H4 N-terminal tails and (C) H2A N-terminal tails at 0.15 M NaCl concentration

are calculated based on C_α of the tail residues over 1 μs simulation. The histogram shows the distribution for three replicas and the solid line represents the average of R_g for replicas. For each tail, the tail-1 (blue) and tail-2 (dark blue) are shown for probability density and average bar plot next to it. The average R_g of three replicas for tails at 0.15 M salt concentrations is obtained by dividing the data into non-overlapping blocks using 11 blocks approximately 91 ns per block for 1 μs simulation.

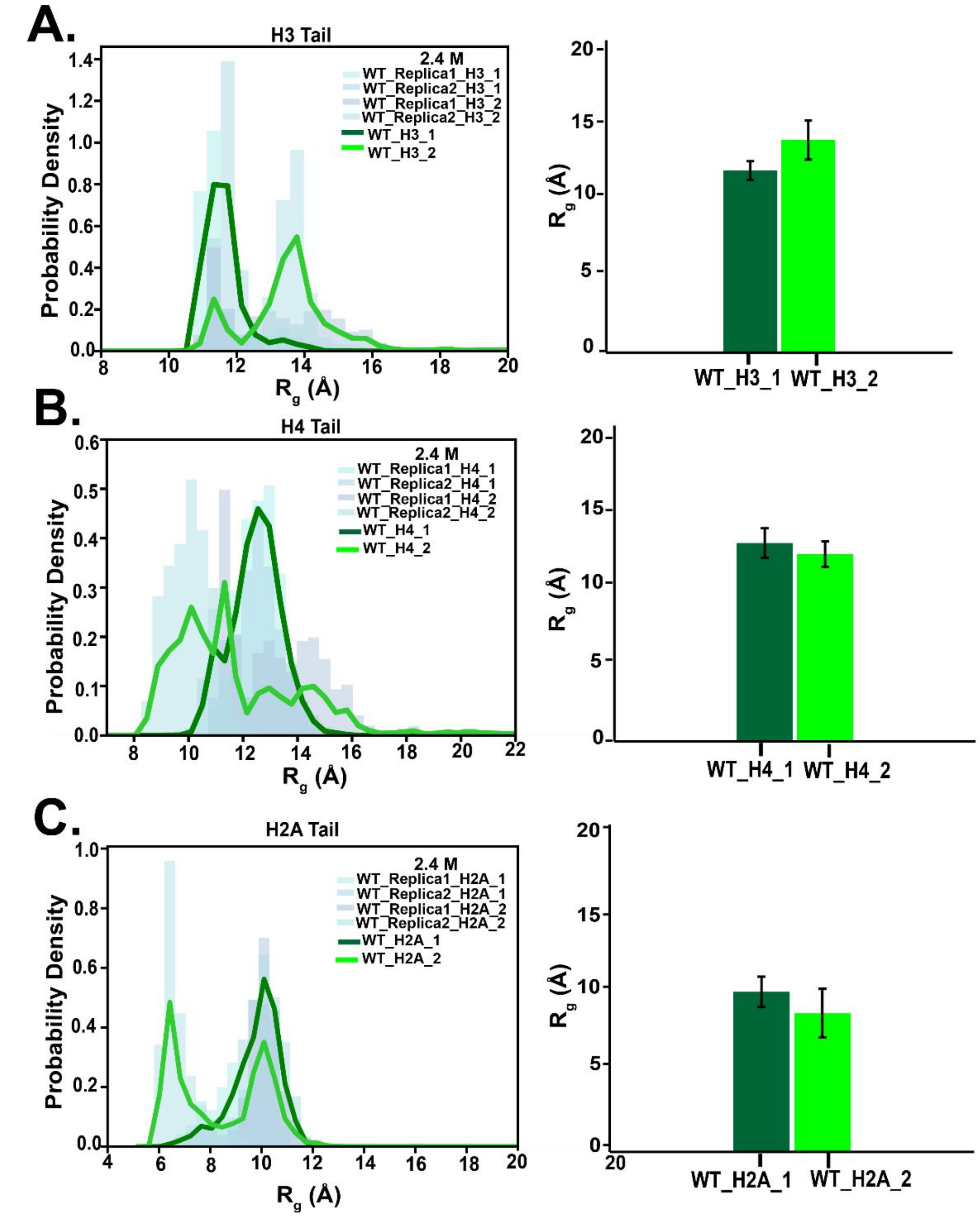

Figure S5. Radius of gyration (R_g) of histone N-terminal tails at 2.4 M salt concentration. (A) The radius of gyration (R_g) of H3 N-terminal tails (B) H4 N-terminal tails and (C) H2A N-terminal tails at 2.4 M NaCl concentration are calculated based on C_α of the tail residues over 1 μs simulation. The histogram shows the distribution for two replicas and the solid line represents the average of R_g for both replicas. For each tail, the tail-1 (green) and tail-2 (light green) are shown for probability density and average bar plot next to it for both tails 1 and 2 of histone tails. The average R_g

of two replicas for tails at 2.4 M salt concentrations is obtained by dividing the data into non-overlapping blocks using 11 blocks approximately 91 ns per block for 1 μs simulation.

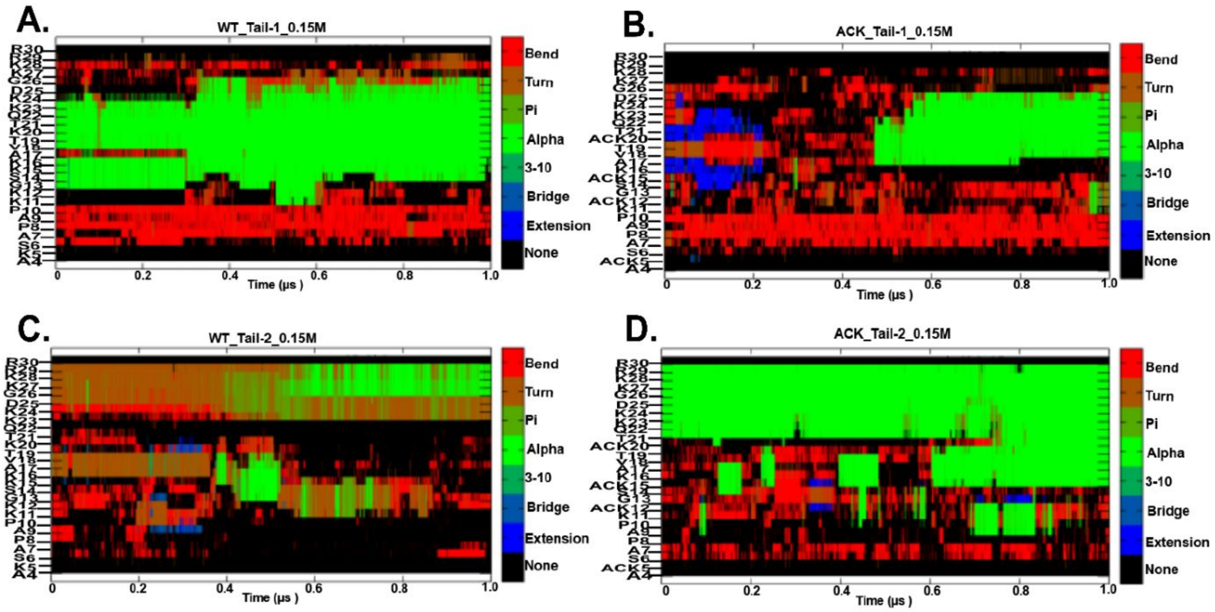

Figure S6. Secondary structure of H2B N-terminal tails in WT and ACK systems at 0.15 M salt concentration. **(A)** The WT H2B tail-1 shows mostly bends (red), none (black), and helix (green) structures throughout the simulation. The helical structure (green) is consistent between G13 to K24 residues and fluctuates slightly. **(B)** The ACK H2B tail-1 forms mainly bend (red), none (black), and some stable beta sheets (blue) between S14 and K24, which later shifted into the consistent helical (green) structure. **(C)** The WT H2B tail-2 shows more turns (brown), none (black), and helical (green) structures. The helical structures (green) shift between G13 to T19 and K24 to R29 residues. **(D)** Upon acetylation of H2B tail -2, it consistently forms a more helical structure between T21 and R29 with flickering helix formation between A9 and ACK20 residues.

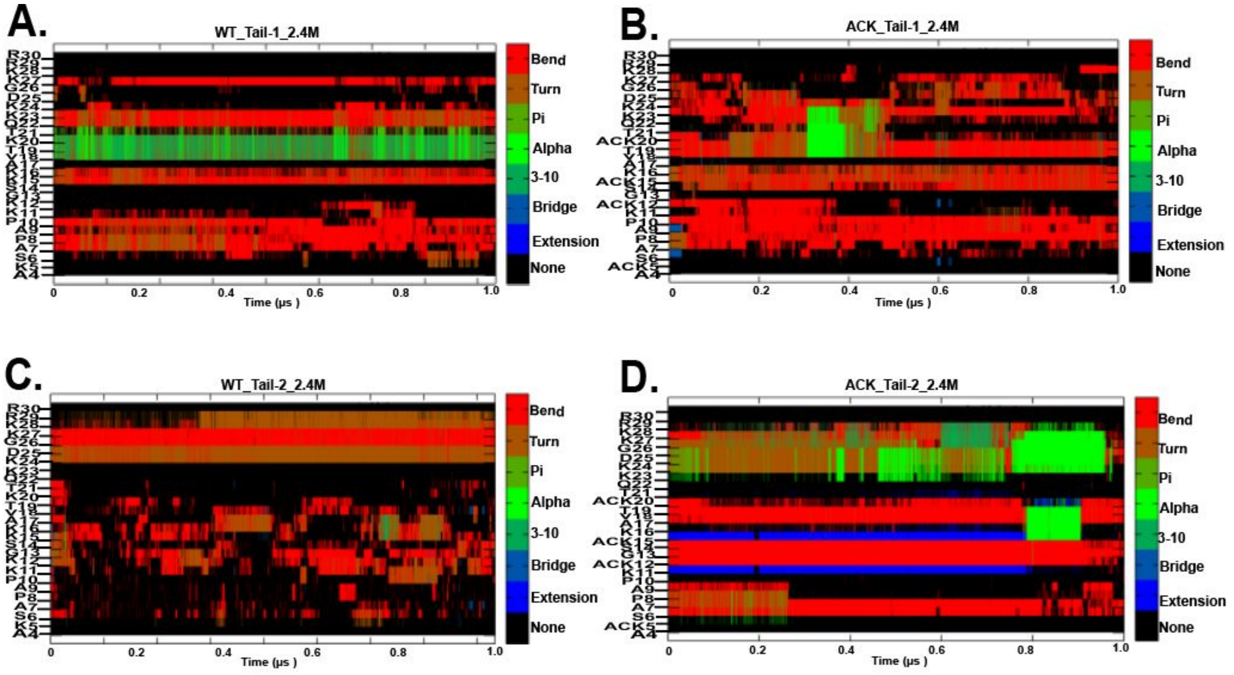

Figure S7. Secondary structure of H2B N-terminal tails in WT and ACK systems at 2.4 M salt concentration. (A) The WT H2B tail-1 shows mostly bends (red), none (black), turns(brown), and helix (green) structures throughout the simulation. The helical structure (green) is consistent between A17 to Q22 residues and flickers throughout the simulation. **(B)** The ACK H2B tail-1 forms mostly bends (red), none (black), and some helices (green) between A17 and K24 for a few nanoseconds. **(C)** The WT H2B tail-2 shows more turns (brown), none (black), and bent (red) structures. The helical structures (green) rarely occur between S14 to T19 and K24 to R29 residues. **(D)** Upon acetylation of H2B, tail-2 forms a more helical structure (green) between K23 and R29 and flickering helix formation, among other residues.

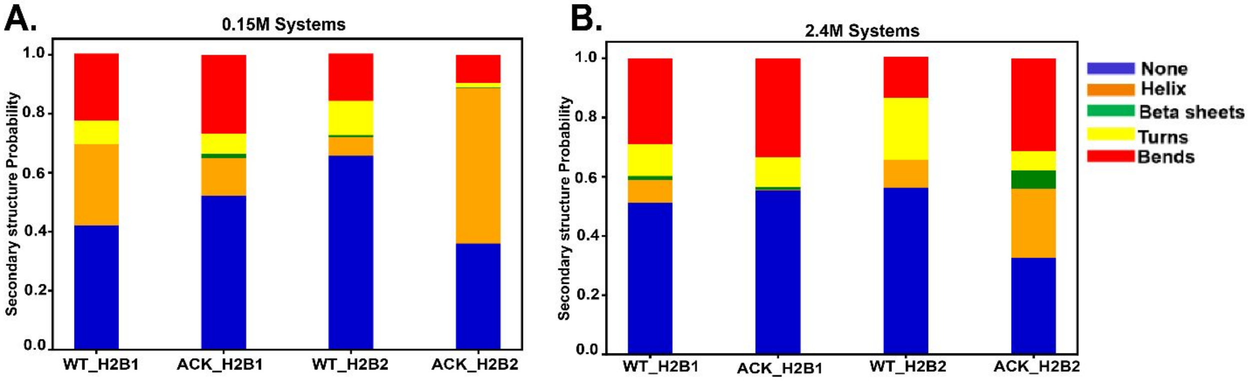

Figure S8. Secondary Structure Propensity of Histone H2B N-terminal tails for replica. (A) and **(B)** The secondary structure formation of WT and ACK H2B N-terminal tails at 0.15 M and 2.4 M NaCl concentrations for replica are represented by different colors: None (blue), Helix (orange), β sheets (green), Turns (yellow), and Bends (red).

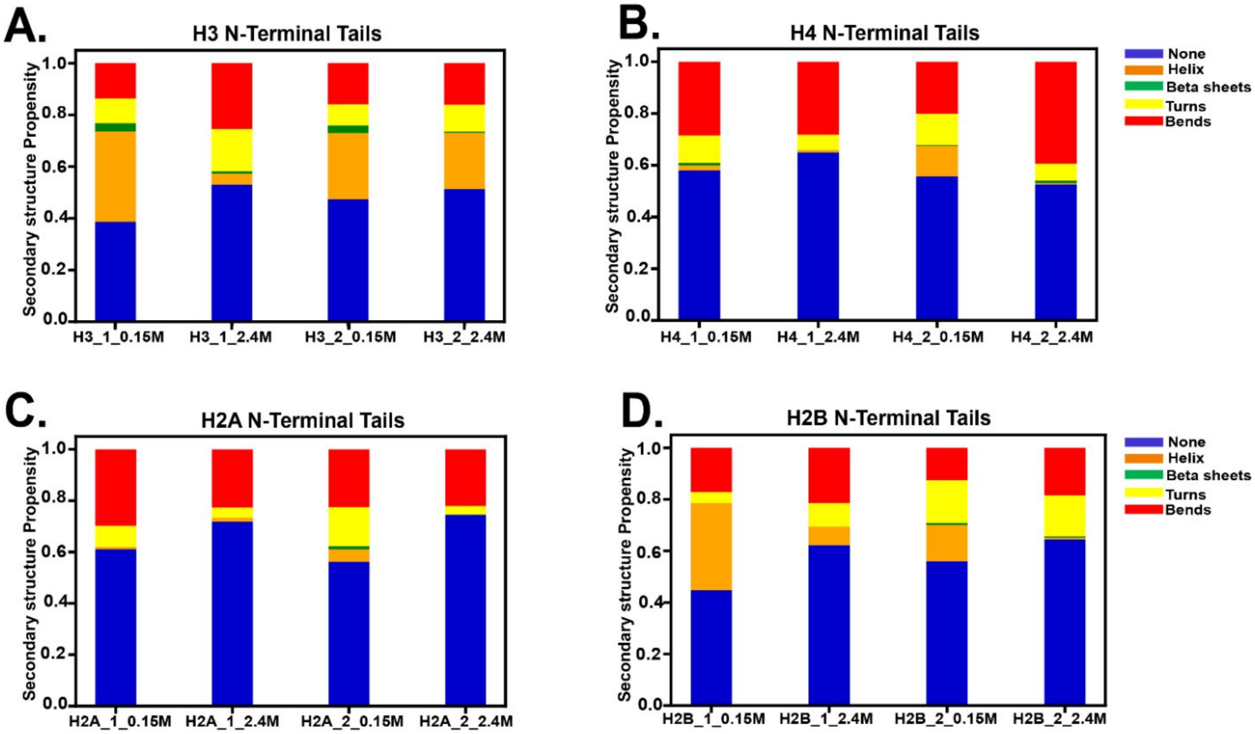

Figure S9. Secondary Structure Propensity of Histone N-terminal tails. The secondary structure formation of N-terminal histone tails at 0.15 M and 2.4 M NaCl concentrations are represented by different colors: None (blue), Helix (orange), β-sheets (green), Turns (yellow), and Bends (red). **(A)** H3 N-terminal tail both tail-1 and tail-2 at 0.15 M (H3_1_0.15M and H3_2_0.15M) show more helical ordered secondary structure compared to both tails (H3_1_2.4M and H3_2_2.4M) at 2.4 M salt concentration. **(B)** H4 N-terminal tail both tail-1 and tail-2 at 0.15 M (H4_1_0.15M and H4_2_0.15M) show more helical ordered secondary structure compared to both tails (H4_1_2.4M and H4_2_2.4M) at 2.4 M salt concentration. **(C)** H2A N-terminal tail has less ordered helical structure compared to other H3, H4, and H2B tails. H2A both tail-1 and tail-2 show slight to almost no helical structure at 2.4 M salt concentration. **(D)** H2B N-terminal tail both tail-1 and tail-2 at 0.15 M (H2B_1_0.15M and H2B_2_0.15M) show more helical ordered secondary structure compared to both tails (H2B_1_2.4M and H2B_2_2.4M) at 2.4 M salt concentration.

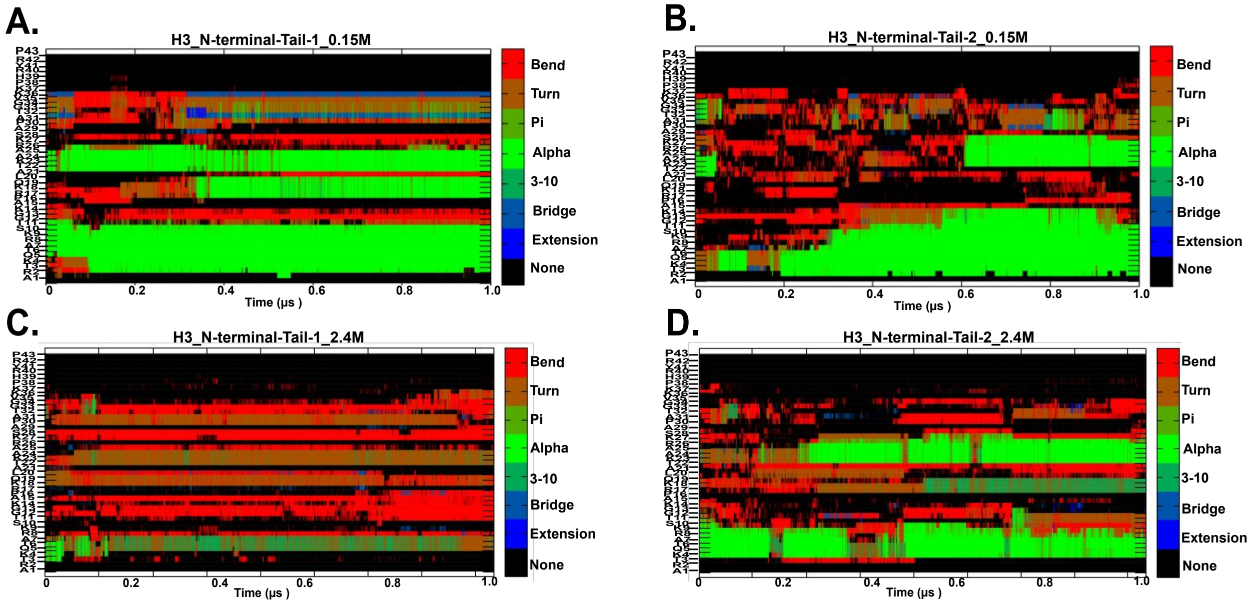

Figure S10. Secondary structure of H3 N-terminal tails at 0.15 M and 2.4 M salt concentrations. (A) The H3 tail-1 shows mostly bends (red), none (black), turns(brown), and helix (green) structures throughout the simulation. The helical structure (green) is consistent between T3 and G12 residues and A21 and A25 throughout the simulation at 0.15 M salt concentration. **(B)** The H3 tail-2 forms mostly bends (red), none (black), and some helices (green) between R2 and G13 at 0.15 M salt concentration. **(C)** The H3 tail-1 shows more turns (brown), none (black), and bend (red) structures at 2.4 M salt concentrations. The helical structures (green) rarely occur between T3 and A7 residues. **(D)** H3 tail -2 forms some helical structure (green) between K4 and K9 and flickering helix formation between T22 and R26 at 2.4 M salt concentrations.

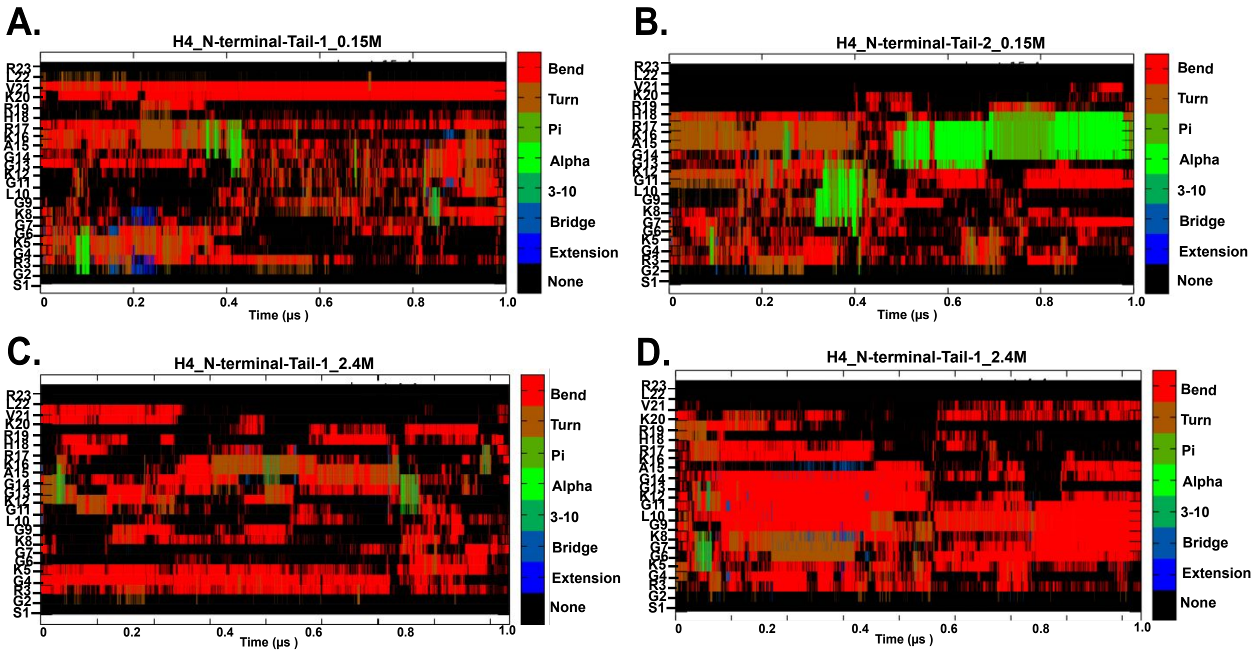

Figure S11. Secondary structure of H4 N-terminal tails at 0.15 M and 2.4 M salt concentrations. (A) The H4 tail-1 shows mostly bends (red), none (black), turns(brown), and helix (green) structures throughout the simulation. The helical structure (green) is flickering around 0.2 μs between G2 and G7 and 0.4 μs between K12 and R17 at 0.15 M salt concentration. **(B)** The H4 tail-2 forms mostly bends (red), none (black), and some helices (green) between G6 and K12 around 0.4 μs and between G13 and R17 from 0.5 μs till 1 μs at 0.15 M salt concentration. **(C)** The H4 tail-1 shows

more turns (brown), none (black), and bend (red) structures at 2.4 M salt concentrations. The helical structures (green)are very rarely occurring. **(D)** H4 tail -2 forms a rare to almost no helical structure (green) throughout the simulation at 2.4 M salt concentrations.

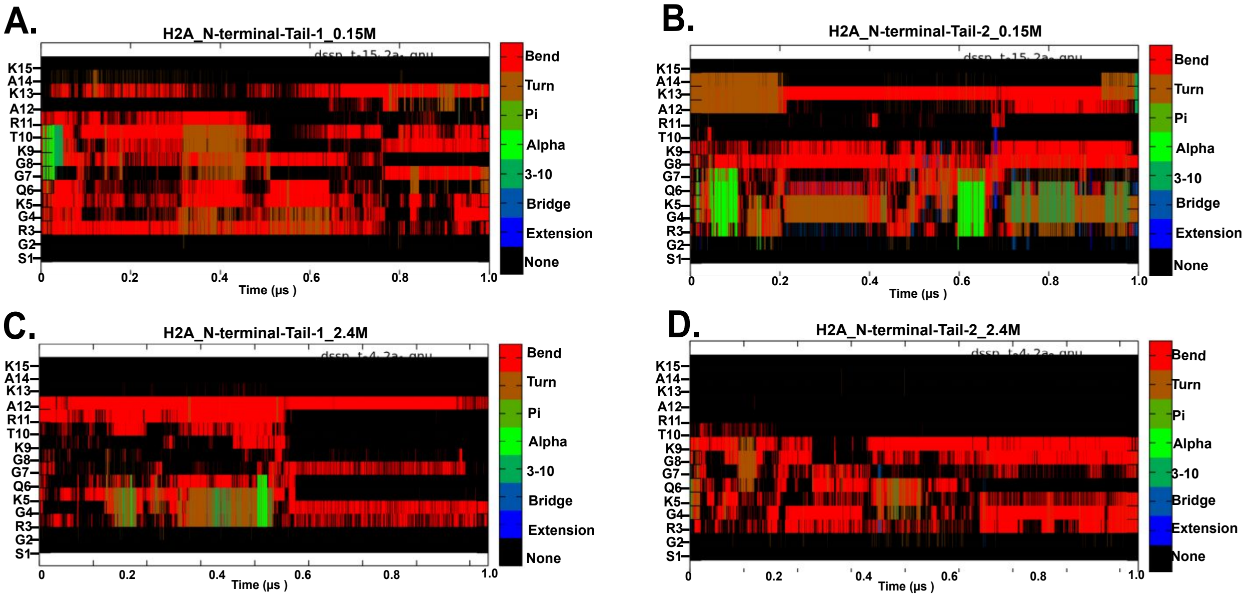

Figure S12. Secondary structure of H2A N-terminal tails at 0.15 M and 2.4 M salt concentrations. (A) The H2A tail-1 shows mostly bends (red), none (black), turns(brown), and helix (green) structures throughout the simulation. The helical structure (green) is very low to none at 0.15 M salt concentration. **(B)** The H2A tail-2 forms mostly bends (red), none (black), and some helices (green) between R3 and G7 around 0.1 μs and again around 0.6 μs at 0.15 M salt concentration. **(C)** The H2A tail-1 shows more turns (brown), none (black), and bend (red) structures at 2.4 M salt concentrations. The helical structures (green) are very rarely occurring. **(D)** H2A tail-2 forms a rare to almost no helical structure (green) throughout the simulation at 2.4 M salt concentrations.

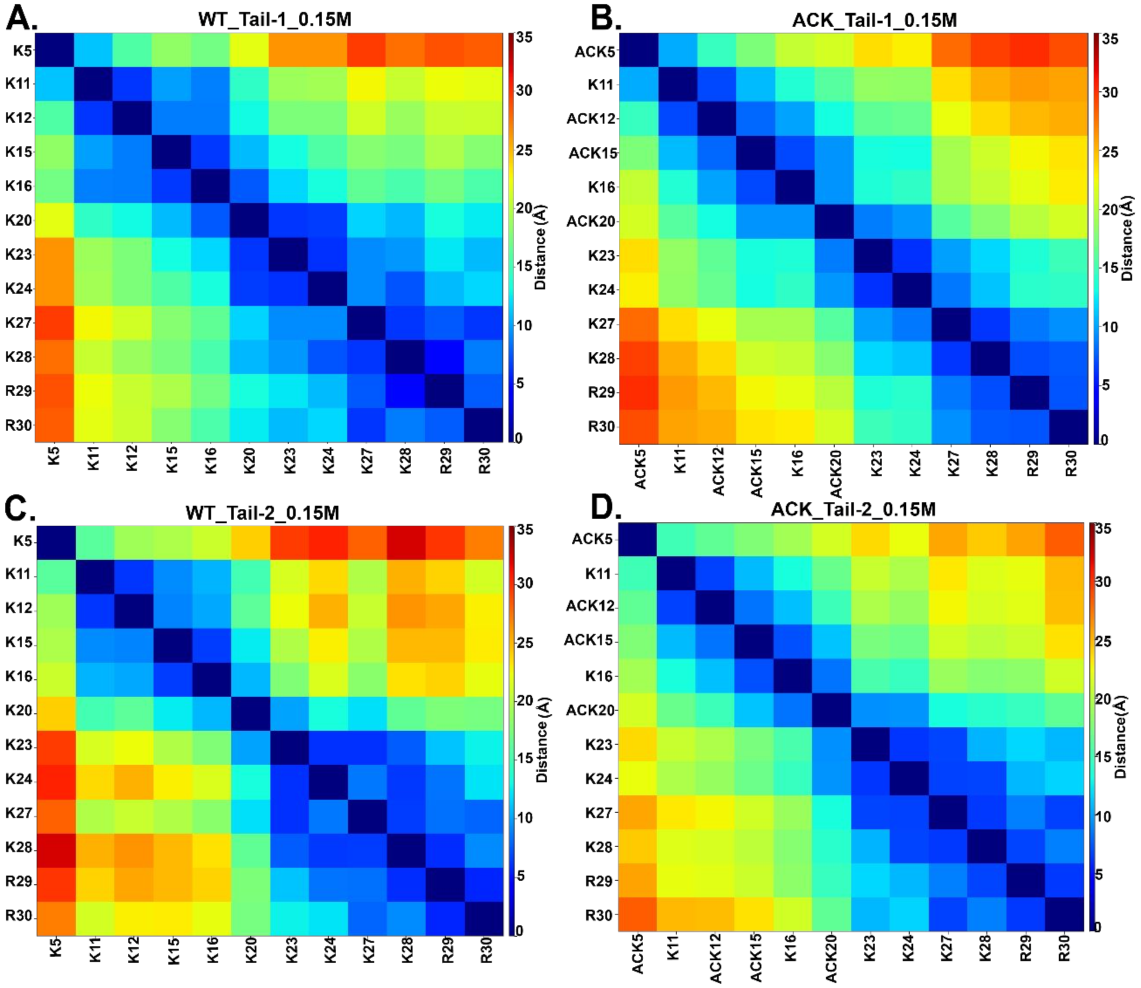

Figure S13. Average distances between positively charged lysine and arginine residues of H2B N-terminal tails at 0.15 M salt concentration. (A) The average distance of WT H2B tail-1 between lysine and arginine positively charged residues with higher distances as electrostatic repulsion occurs. **(B)** The average distance of ACK H2B tail-1 can shorten the distance between residues as it reduces the electrostatic repulsions with some similarity to WT tail-1 as not all the lysine residues are acetylated. **(C)** The average distance of WT H2B tail-2 between lysine and arginine positively charged residues with higher distances as electrostatic repulsion occurs. **(D)** The average distance of ACK H2B tail-2 can shorten the distance between residues as it reduces the electrostatic repulsions upon acetylation.

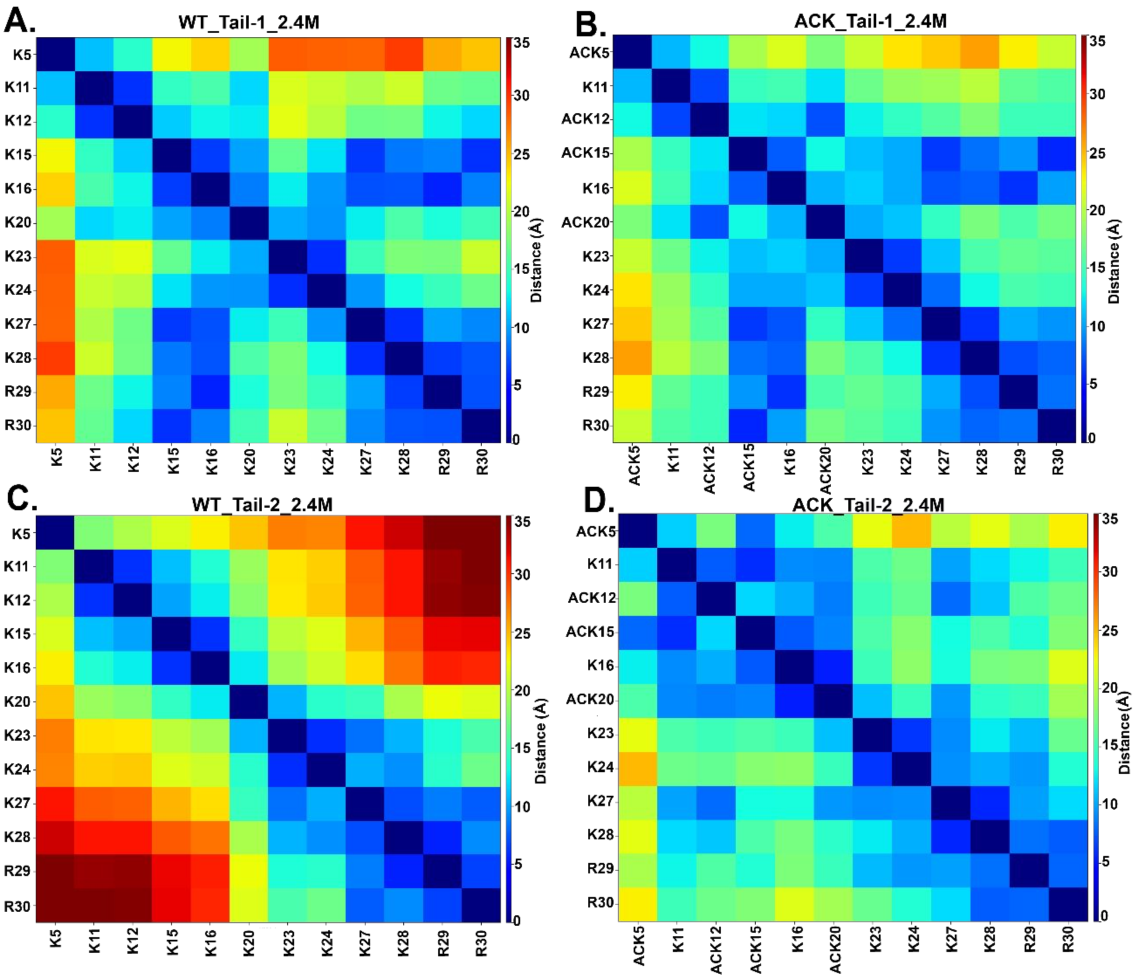

Figure S14. Average distances between positively charged lysine and arginine residues of H2B N-terminal tails at 2.4 M salt concentration. (A) The average distance of WT H2B tail-1 between lysine and arginine positively charged residues with higher distances as electrostatic repulsion occurs. **(B)** The average distance of ACK H2B tail-1 can shorten the distance between residues as it reduces the electrostatic repulsions upon acetylation. **(C)** The average distance of WT H2B tail-2 between lysine and arginine positively charged residues with higher distances as electrostatic repulsion occurs. **(D)** The average distance of ACK H2B tail-2 can shorten the distance between residues as it reduces the electrostatic repulsions upon acetylation.

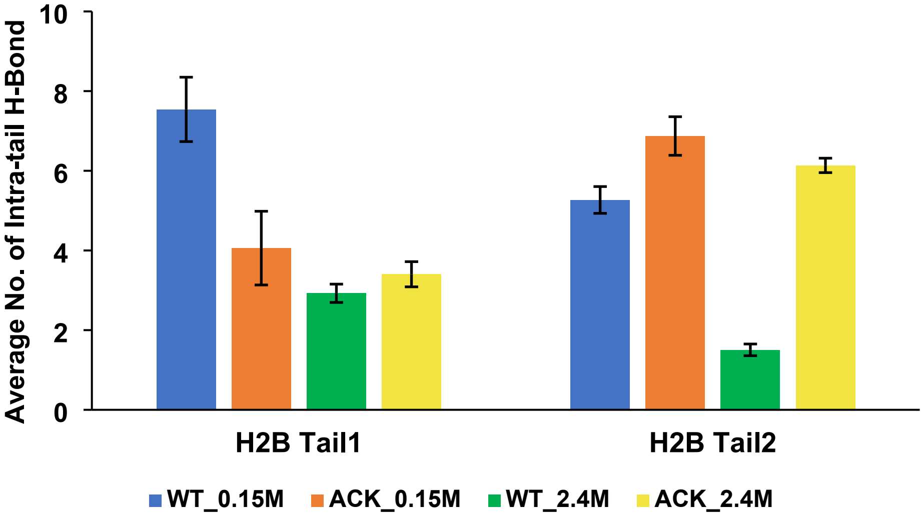

Figure S15. Average number of intra-tail hydrogen bonds within H2B tail residues. The H2B tail-1 (left) and H2B tail-2 (right) with both 0.15 M and 2.4 M salt concentrations for all WT and ACK tails show a number of hydrogen bonds within the tail, which can correlate with the formation of helices in secondary structure formation.

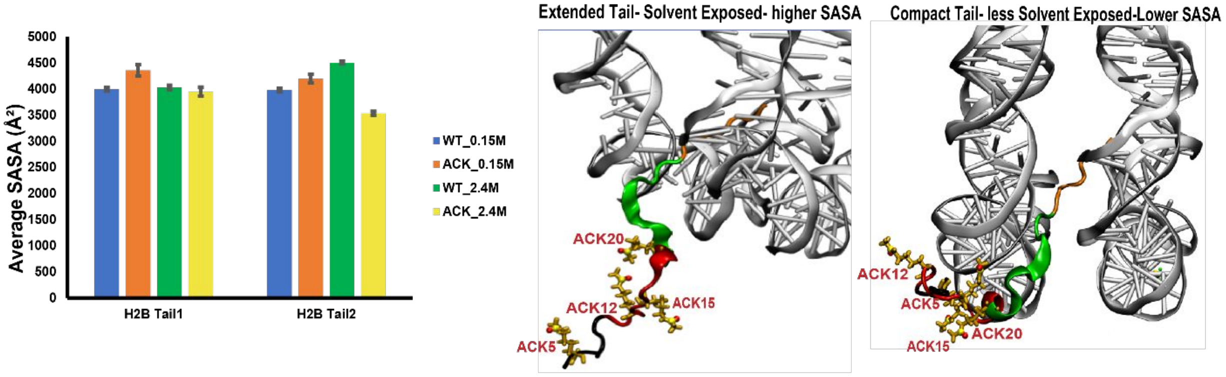

Figure S16. Average Solvent Accessible Surface Area (SASA) of H2B N-terminal tails. The average SASA for H2B tails in WT and ACK systems at 0.15 M and 2.4 M salt concentrations is obtained by dividing the data into ten non-overlapping blocks. The tail conformations when the tail is released from DNA and exposed to solvent (left) and the tail when it turns back towards the DNA (right).

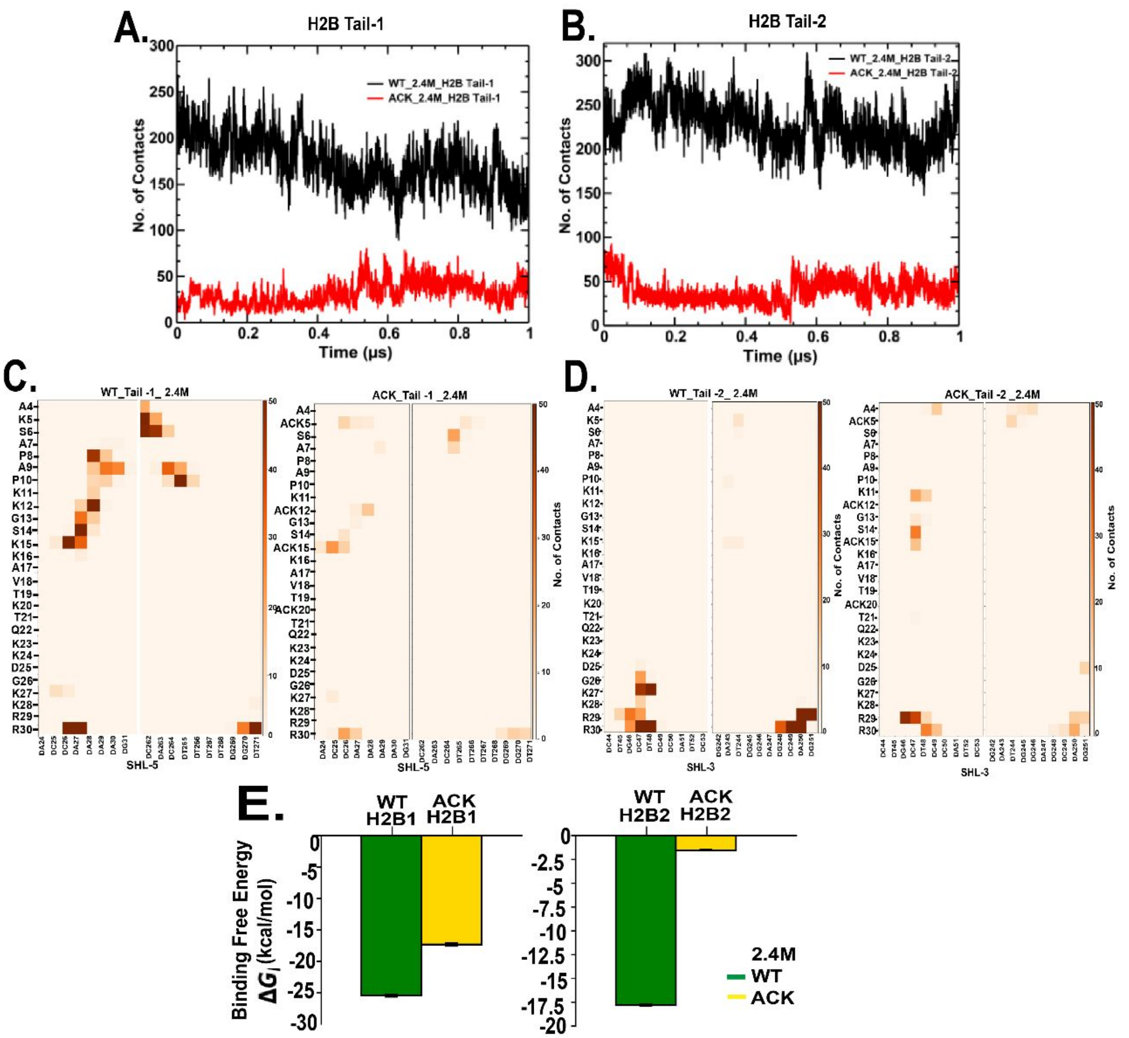

Figure S17. DNA-Histone H2B N-terminal tails Contacts Analysis and binding free energy at 2.4 M salt concentration. (A) and **(B)** The number of contacts between the H2B N-terminal tail and DNA as a function of time for 2.4 M NaCl concentration over 1 μs simulation with 4.5 Å cutoff distance show a decrease in ACK (red) tails upon acetylating four lysine residues of tails compared to WT (black). **(C)** and **(D)** The contact maps show the number of contacts of WT and ACK tails between specific tail residues and DNA base pair of SHL-5 and SHL-3 for H2B tail-1 and 2, respectively. Overall, contact maps also show a decrease in the number of contacts upon acetylation. **(E)** The binding free energy calculated for WT (green) and acetylated (yellow) systems for both H2B tails with DNA SHL±5 regions indicated higher binding free energy for the WT system than ACK.

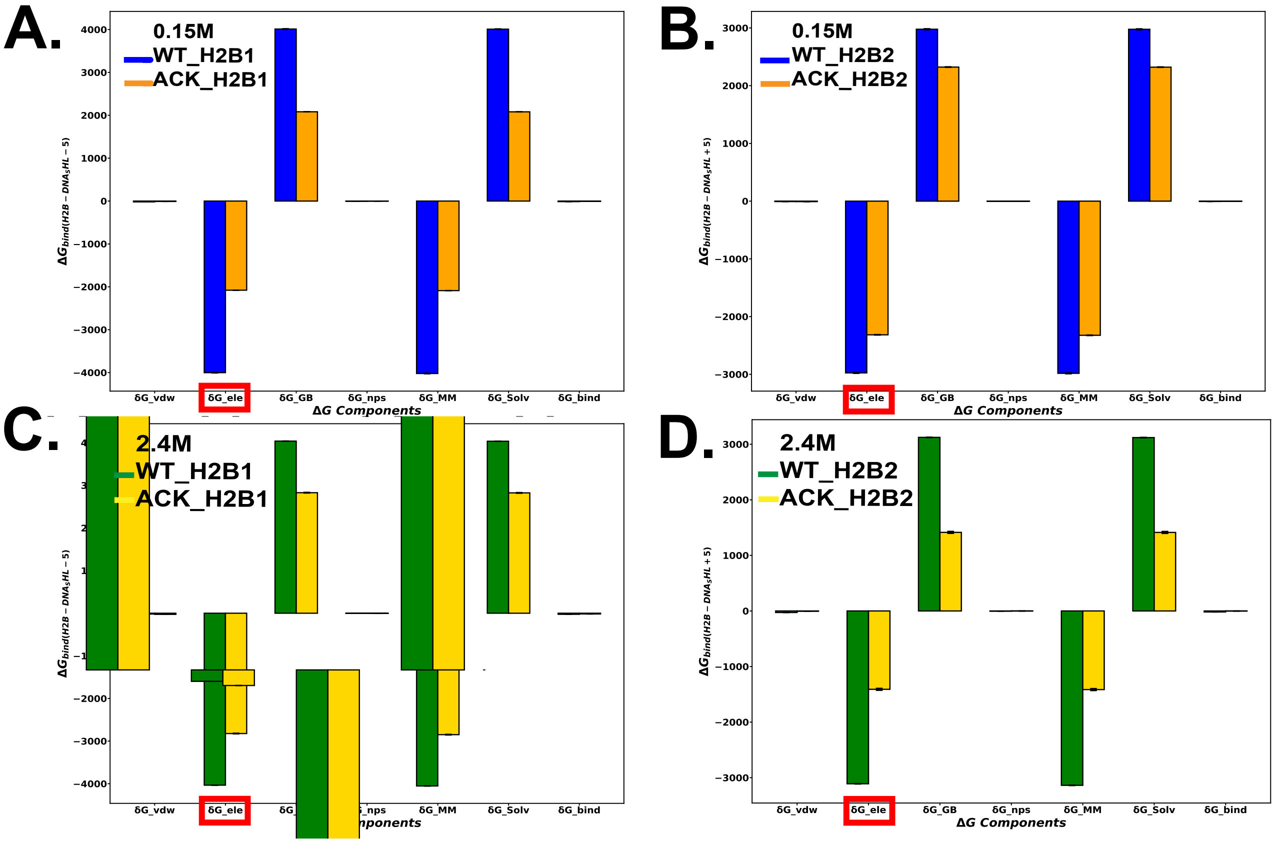

Figure S18. Binding free energy components. (A) and **(B)** show contributions of different components to the binding free energy at 0.15 M salt concentration for H2B tail-1 and tail-2 with DNA SHL±5 regions. The highlighted red is the electrostatic interaction, a significant contribution to binding free energy between the tail and DNA. **(C)** and **(D)** show contributions of different components to the binding free energy at 2.4 M salt concentration for H2B tail-1 and tail-2 with DNA SHL±5 regions. The highlighted red is the electrostatic interaction, a significant contribution to binding free energy between the tail and DNA. Other interactions between Van der Waals (vdw) and non-polar (NPS) are minimal between the H2B tail and DNA region.

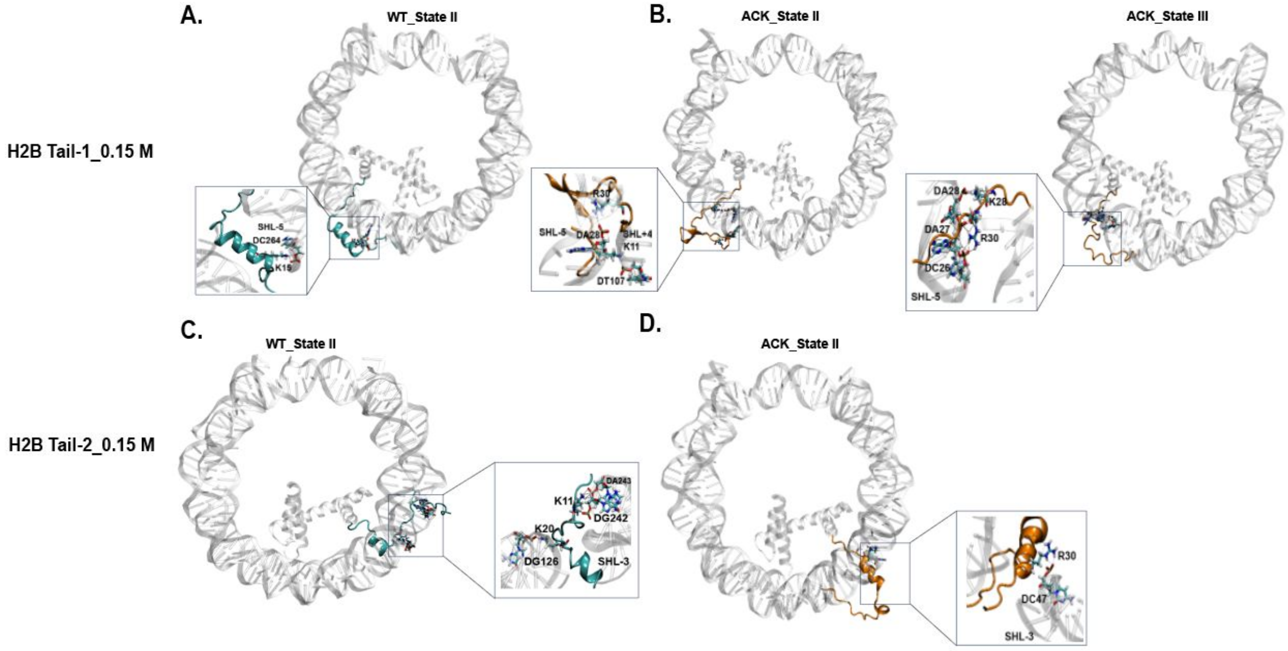

Figure S19. H2B Tail Conformations for 0.15 M salt concentration. The conformation states of the N-terminal H2B tail extracted from the PCA free energy surface are shown in Figure 5 and Figure 6 in the main text. This conformation is shown for the location of the tail concerning DNA.

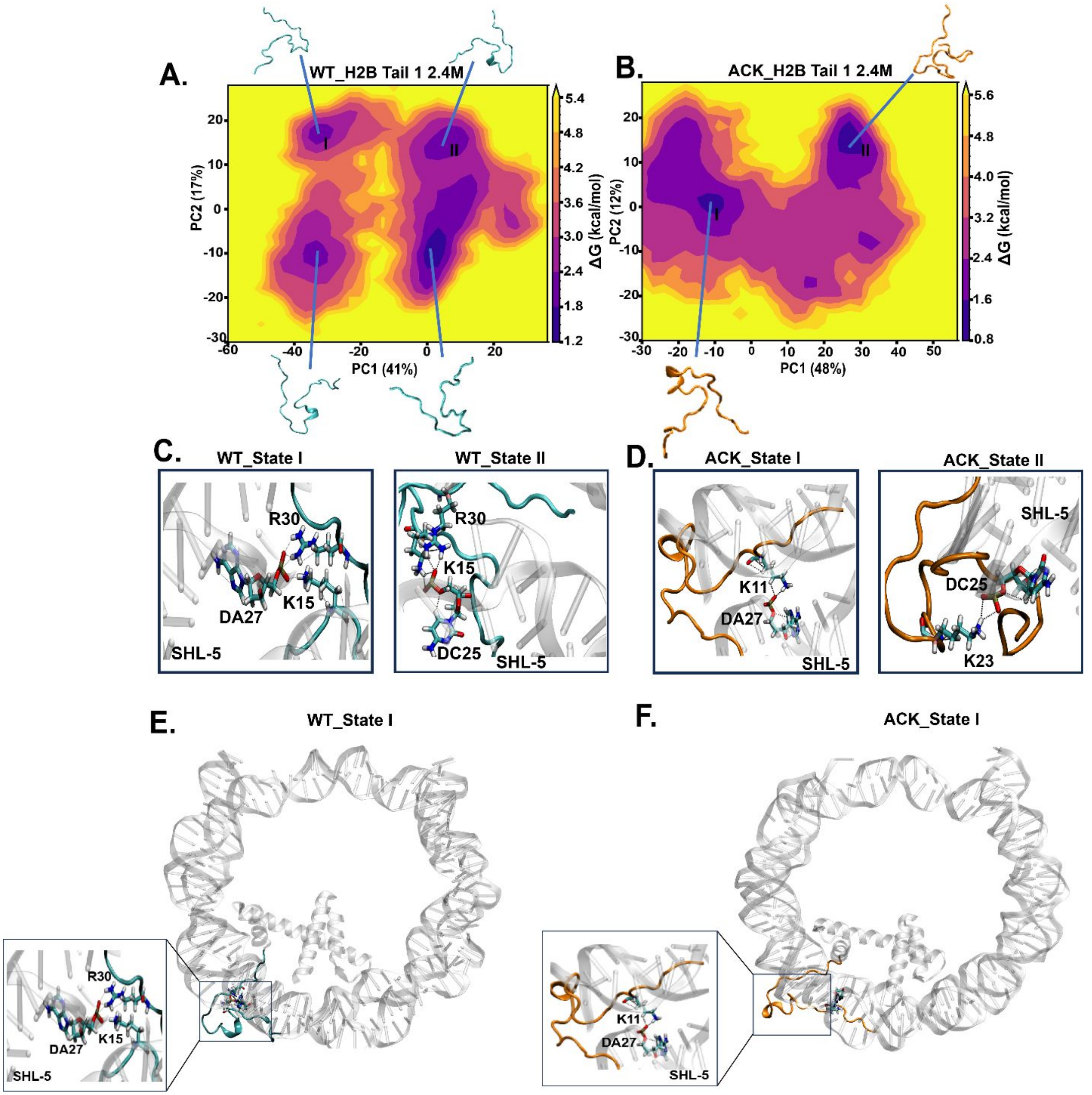

Figure S20. Identifying H2B N-terminal tail-1 dynamics upon acetylation using Principal Component Analysis (PCA). (A) and **(B)** The PCA analysis is performed to study the collective modes of the H2B N-terminal tail-1 of WT and ACK for 2.4 M for 1 μs simulation. The energy landscape is constructed using the first two principal components (PC) for WT and ACK H2B tail-1 and generates more concentrated central minima to which major tail conformations states belong. **(C) and (D)** The H2B tail conformations obtained from PCA free energy surface show tail-DNA interactions between DNA base pairs and positively charged residues of the H2B N-terminal tail. The WT H2B tail-1 states I and II exhibit hydrogen bonds between K15 with the phosphate backbone of DA27 and K15 with DC25 of the SHL-5 region. The ACK H2B tail-1 states I and II exhibit hydrogen bonds between K11 and K23 with DA27 and DC25, respectively. Also, WT states I and II exhibit hydrogen bonds between R30 and DA27 of the SHL-5 region. **(E) and (F)** The H2B tail conformation state I of WT and ACK tail's location concerning DNA. (see Figure S22 for other states)

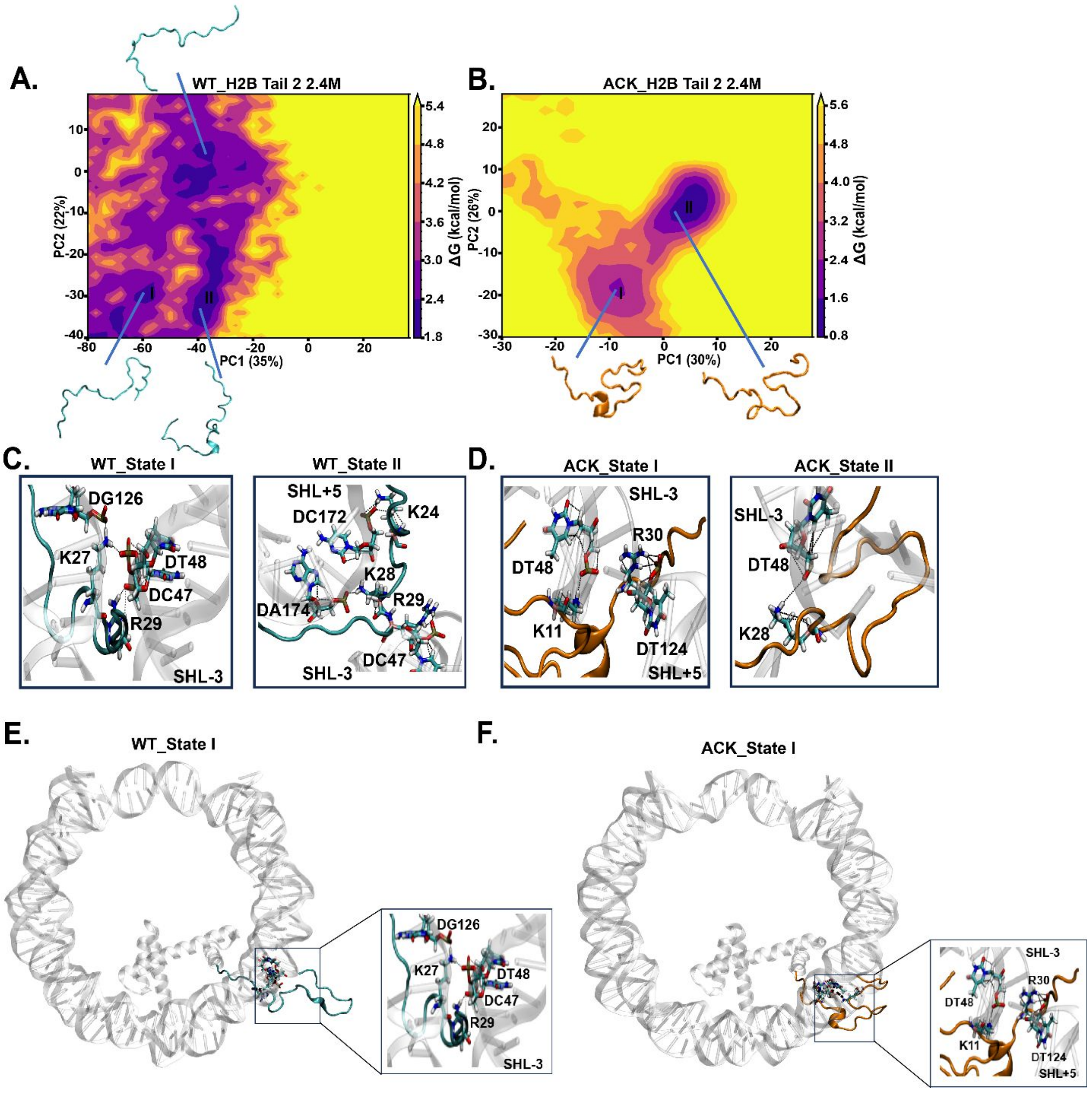

Figure S21. Identifying H2B N-terminal tail-2 dynamics upon acetylation using Principal Component Analysis (PCA). (A) and **(B)** The PCA analysis is performed to study the collective modes of the H2B N-terminal tail-2 of WT and ACK for 1 μS simulation. The energy landscape is constructed using the first two principal components (PC) for WT and ACK H2B tail-2 and generates more concentrated central minima to which major tail conformations states belong. **(C) and (D)** The H2B tail conformations obtained from PCA free energy surface show tail-DNA interactions between DNA base pairs and positively charged residues of the H2B N-terminal tail. The WT H2B tail-2 states I and II exhibit hydrogen bonds between K11 with the phosphate backbone of DG242 and K20 with DG126, respectively. The WT H2B tail-2 state I exhibit a hydrogen bond between R29 and DG46. The ACK H2B tail-2 states I and II exhibit hydrogen bonds between K28 with DA147 and R30 with DC47, respectively. **(E) and (F)** The H2B tail conformation state I of WT and ACK tail's location concerning DNA. (see Figure S22 for other states)

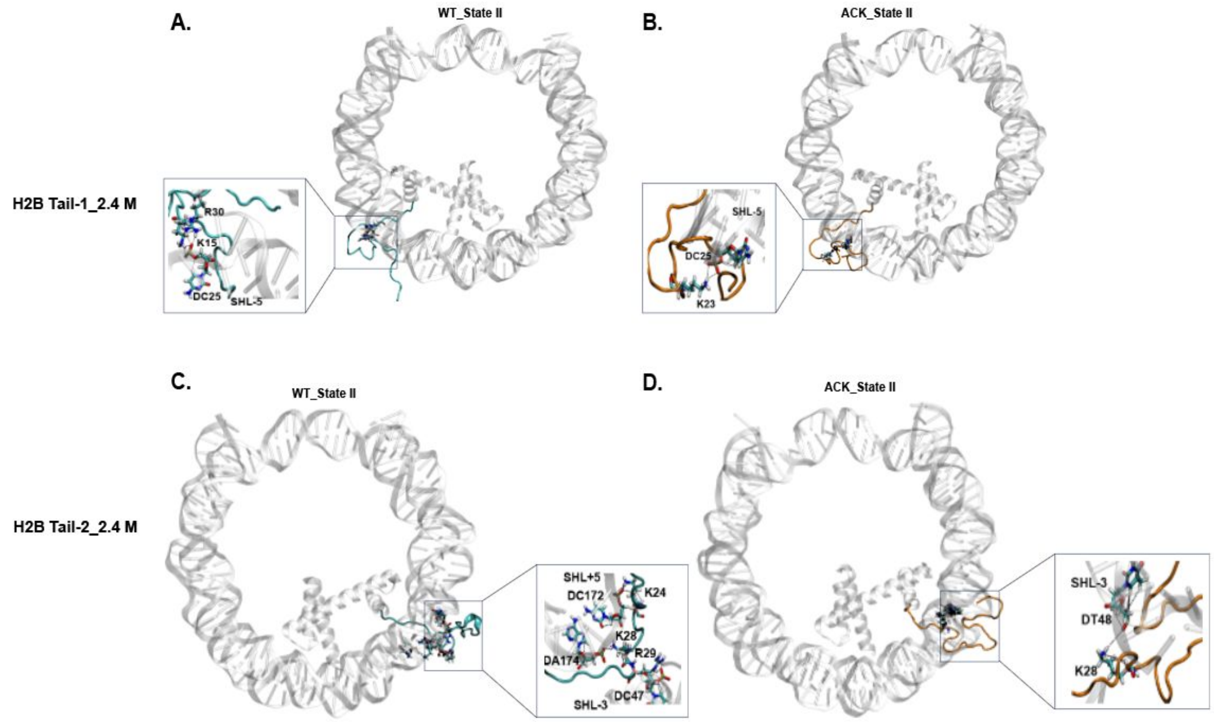

Figure S22. H2B Tail Conformations for 2.4 M salt concentration. The conformation states of the N-terminal H2B tail extracted from the PCA free energy surface are shown in Figures S20 and S21. These conformations are shown for the location of the tail concerning DNA.

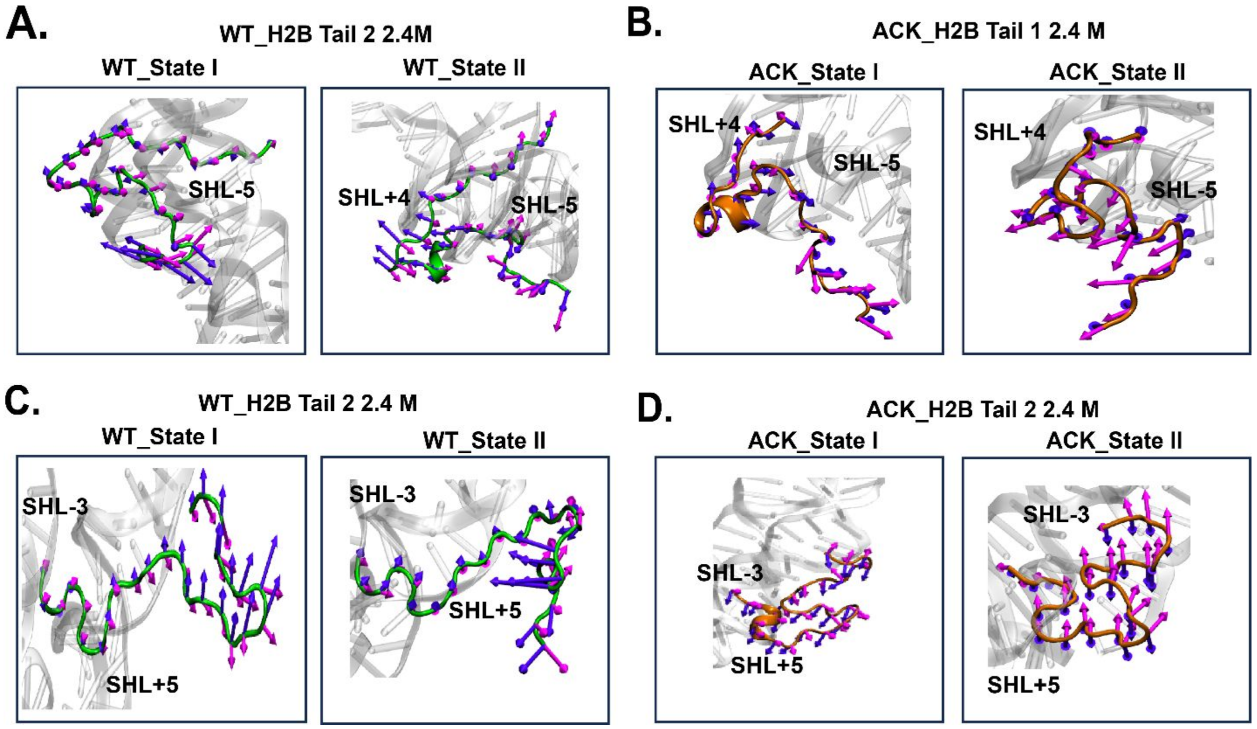

Figure S23. Porcupine plots of H2B tails. Porcupine plots are drawn to visualize the movements of the first eigenvector obtained from the PCA. The dominant motions of C_α atoms of tail residues in (A) WT H2B tail-1 (green)

(B) ACK H2B tail-1 (orange) (C) WT H2B tail-2 (green) and (D) ACK H2B tail-2 (orange) are indicated with arrows for each conformations in blue (PC1) and magenta (PC2) color. The arrows depict the direction of movements of each conformations The magnitude of motions is illustrated by the length of arrows.

Radial distribution of ions

The primary DNA-histone interactions between the negatively charged phosphate group of the DNA backbone and the positively charged side chains of lysine and arginine residues can be modulated by the ions present in the surroundings of the nucleosome. Therefore, it is essential to analyze the distribution of ions around the DNA of the nucleosome and the highly charged histone tails. The higher radial distribution of ions indicates a greater probability of ions binding to the DNA [5] . Since the H2B tails protrude from the histone core between the two DNA gyres around SHL±5, and the same DNA region has more contact with the H2B tails, we analyze the ion environment around this region. Therefore, the radial distribution function (g(r)) of the sodium ions (Na^+) is plotted around the SHL+5 and SHL-5 regions of the nucleosomal DNA for both the 0.15 M and 2.4 M salt concentrations (**Figure S24**). As there are two salt concentrations, we also calculate the Debye length[6] $\lambda_{D=\sqrt{\frac{\varepsilon_r \varepsilon_{0kT}}{\Sigma(ze)^2 c_i}}}$ at both salt concentrations. The Debye length for 0.15 M salt concentration is 0.8 nm and for 2.4 M salt concentrations is 0.2 nm at 310 K temperature. At 2.4 M salt concentration, the Debye length is shorter, indicating that electrostatic effects do not extend out very far. At 0.15 M salt concentration, the Debye length is longer, indicating that electrostatic effects extend out at greater distances. The radial distribution analysis for 0.15 M (**Figure S24A**) shows the peak between 4-6 Å radius, whereas, for 2.4 M (**Figure S24C**), the peak appears between 2-4 Å radius. As the Debye length at 2.4 M is 0.2 nm, which is shorter than 0.15 M, the Na^+ radial distribution, for WT at 2.4 M, the peak is shifted at a closer radial distance compared to 0.15 M WT. (**Figure 24A, 24C**).

As we show the number of contacts decreases upon acetylation as charge neutralization disrupts the DNA-H2B tail contacts, this could make more Na^+ ions come in where the histone positively charged residues bind to DNA to compensate the loss of the interaction with the acetylated tails. As a result, the probability of ion distribution would increase as ions move closer to DNA and interact with a negatively charged backbone of DNA (**Figure S24B**).

As mentioned earlier, histone tails can be considered polyelectrolytes. One of the features of polyelectrolytes is that they can undergo rapid contraction into a more compact form as the counterion concentration increases[7-10]. To understand the ion distribution around histone tails, we calculate the radial distribution function for Cl^- ions around the H2B tails with the positively charged Lys and Arg residues for both the 0.15 M and 2.4 M salt concentrations. Earlier, we have observed that at 2.4 M salt concentration, the acetylated tails shift the secondary structure to form more helices as the tail has less charge repulsion and a more hydrophobic nature compared to WT. Also, the accumulations of ions around the side chain of the positively charged residues of the tail would increase ionic screening and reduce the charge repulsion. Therefore, the acetylated tails that extend out more would contract. We have observed this contraction for the acetylated tails at 2.4 M salt concentration.

We calculate the radial distribution of Cl^- ions around the positively charged histone tail residues for both the WT and ACK systems at 0.15 M and 2.4 M salt concentrations (**Figure S25**). We observe that for WT tail-1 at 0.15 M, the peak in the radial distribution of Cl^- ions is slightly higher than tail-2 (**Figure S25A**). Similarly, we observe ACK tail-2 has a slightly higher Cl^- peak than tail-1 (**Figure S25B**). This tail-2 shows an increase in helical structure. Acetylation of the tail adds to the hydrophobic nature of the tail as neutralization of lysine reduces charge repulsion and tail contracts, as we have seen earlier. Similarly, for the 2.4 M salt concentration, the acetylated H2B tail shows even more contraction compared to the WT tail. The peak in the radial distribution of Cl^- ions for the acetylated tail-1 is slightly higher than tail-2 (**Figure S24D**). Counterions around the positively charged residues of the acetylated tail promote tail contraction.

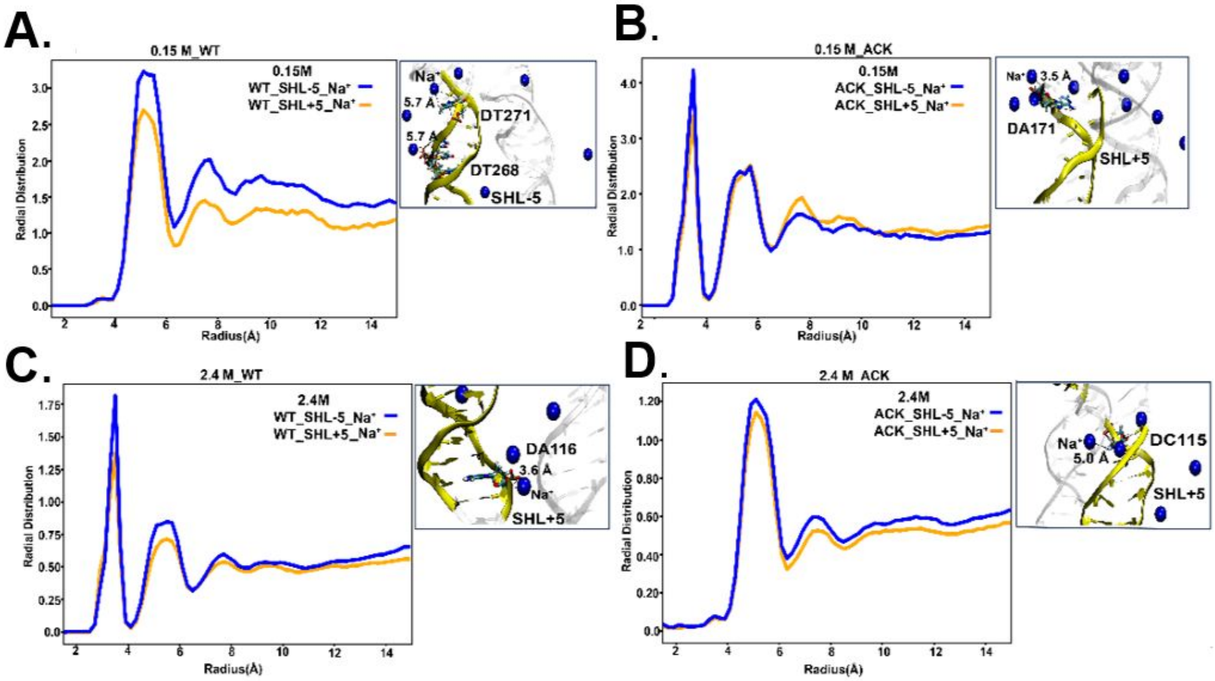

Figure S24. Radial Distribution of Na^+ ions around SHL±5 DNA regions. (A) The radial distribution function of SHL-5 (blue) and SHL+5 (orange) with Na^+ ions of WT at 0.15 M NaCl concentration show one major peak between 4 Å -6 Å. The insert example shows the DNA bases DT268 and DT271 of SHL-5 regions interacting with Na^+ at ~5 Å. **(B)** The radial distribution function of SHL-5 (blue) and SHL+5 (orange) with Na^+ ions of ACK at 0.15 M NaCl concentration show peaks between 2 Å -6 Å. The insert example shows the DNA base DA171 SHL+5 regions interacting with Na^+ at 3.5 Å. **(C)** The radial distribution function of SHL-5 (blue) and SHL+5 (orange) with Na^+ ions of WT at 2.4 M NaCl concentration show peaks between 2 Å -6 Å. The insert example shows the DNA base DA116 SHL+5 regions interacting with Na^+ at 3.6 Å. **(D)** The radial distribution function of SHL-5 (blue) and SHL+5 (orange) with Na^+ ions of ACK at 2.4 M NaCl concentration show peaks between 4 Å -6 Å. The insert shows the DNA base DC115 in the SHL+5 region interacting with Na^+ at ~5.0 Å.

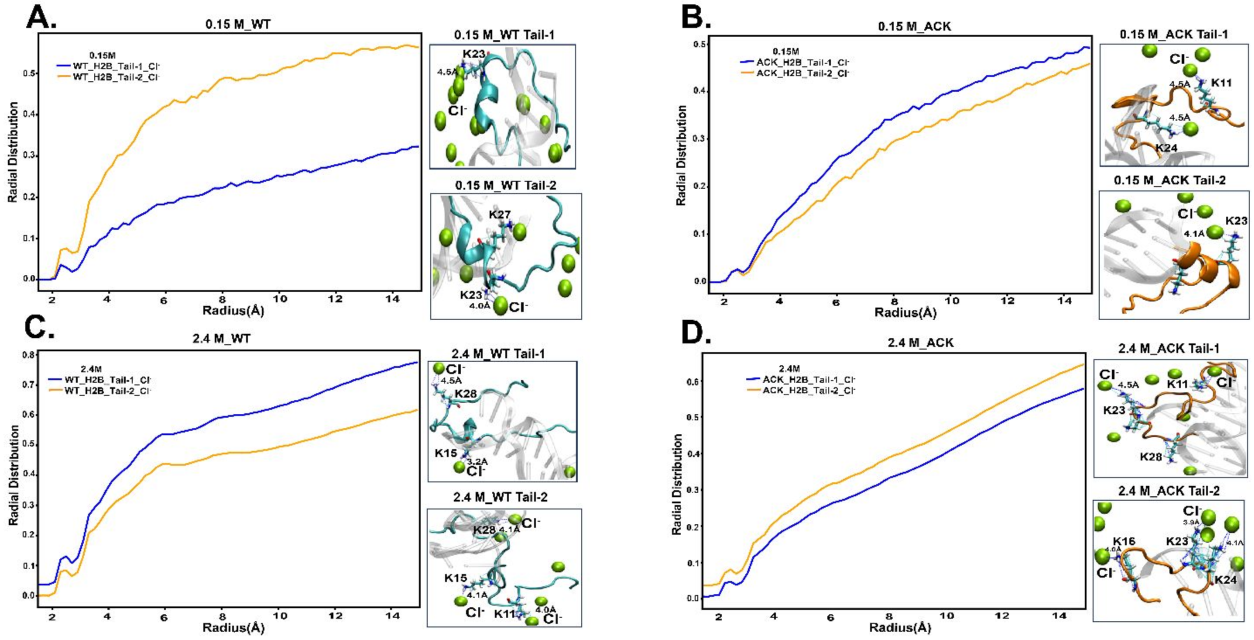

Figure S25. Radial Distribution of Cl^- ions around Histone H2B N-terminal Tails. (A) The radial distribution function of H2B tail-1 (blue) and H2B tail-2 (orange) with Cl^- ions of WT at 0.15 M NaCl concentration. The insert example shows the lysine residues Cl^- at ~4.5 Å. **(B)** The radial distribution function of H2B tail-1 (blue) and H2B tail-2 (orange) with Cl^- ions of ACK at 0.15 M NaCl concentration. The insert example shows the lysine residues Cl^- at ~4-4.5 Å. **(C)** The radial distribution function of H2B tail-1 (blue) and H2B tail-2 (orange) with Cl^- ions of WT at 2.4 M NaCl concentration. The insert example shows the lysine residues Cl^- at ~3.5 Å - 4.5 Å. **(D)** The radial distribution function of H2B tail-1 (blue) and H2B tail-2 (orange) with Cl^- ions of ACK at 2.4 M NaCl concentration. The insert example shows the lysine residues Cl^- at ~3.9 Å -4.5 Å.

Tables

Table S1. Summary of NCP systems MD simulation set up box sizes and atoms/ions for H2B tails.

NCP systems	WT_0.15M (unacetylated)	ACK_0.15M (acetylated)	WT_2.4M (unacetylated)	ACK_2.4M (acetylated)
**Box Size (Å^3)**	159 x 191 x 112	159 x 191 x 112	156 x 187 x 110	156 x 187 x 110
**No. of atoms**	444888	486492	396256	409382
**No. of Water molecules**	104740	115117	90508	92907
**No. of Na^+ ions**	472	508	4620	6373
**No. of Cl^- ions**	356	384	4504	6249
**No. of Mg^{2+} ions**	14	14	14	14
**NaCl salt Concentration (M)**	0.15	0.15	2.4	2.4

Table S2. Hydrogen bonds between DNA and H2B tail residues for WT 0.15 M salt concentration

WT_H2B Tails 0.15 M	SHL regions	Residues	Avg. distance (Å)	Frames	Fraction
**H2B Tail -1**	SHL-5	(R30) HE -OP1 (DA27)	3.24	1172	0.234
	SHL-5	(K23) HZ3 -OP1 (DA28)	3.10	362	0.072
	SHL-5	(R30) HH21 -OP1 (DA27)	3.23	957	0.191
	SHL-5	(K23) HZ1 -OP1 (DA28)	3.16	339	0.072
	SHL-5	(K27) HZ2 -OP1 (DA27)	3.18	327	0.065
	SHL-5	(K27) HZ1 -OP1 (DA27)	3.20	309	0.061
	SHL-5	(K20) HZ1 -OP2 (DA27)	3.42	125	0.025
	SHL-5	(K5) HZ1-OP1 (DC264)	3.26	95	0.019
	SHL-5	(K11) HZ1-OP1(DC264)	3.08	102	0.020
	SHL-5	(K5) HZ1-OP1 (DC264)	3.09	425	0.085
	SHL-5	(K5) HZ1-OP1 (DA263)	2.97	93	0.019
	SHL-5	(K11) HZ2-OP1(DC264)	3.16	91	0.018
	SHL-5	(K11) HZ3-OP1(DC262)	3.43	37	0.007
	SHL-5	(K12) HZ1-OP1(DC262)	3.40	40	0.008
	SHL-5	(K12) HZ3-OP2(DA263)	3.28	47	0.009
	SHL-5	(K15) HZ3-OP2 (DC264)	3.21	162	0.032
	SHL-5	(K16) HZ1-OP2 (DA28)	3.50	122	0.024
	SHL-5	(K20) HZ3-OP2 (DA27)	3.35	137	0.027
	SHL-5	(K23) HZ3-OP1 (DA28)	3.10	362	0.072
	SHL-5	(K27) HZ2-OP1 (DA27)	3.18	327	0.065
	SHL-5	(K20) HZ1-OP2 (DA27)	3.42	125	0.025
	SHL-5	(K20) HZ2-OP2 (DA27)	3.46	115	0.023
	SHL-5	(K20) HZ1-OP2 (DC26)	3.46	46	0.009
	SHL+4	(R30) HE -OP1 (DT105)	3.21	600	0.120
	SHL+4	(R30) HH21 -OP2(DT105)	3.41	421	0.084
	SHL+4	(K20) HZ1-OP2 (DT104)	3.33	280	0.056
	SHL+4	(K20) HZ2-OP2 (DT104)	3.22	273	0.054
	SHL+4	(K24) HZ1 -OP1(DT106)	3.18	372	0.074
	SHL+4	(K24) HZ2 -OP1(DT106)	3.21	362	0.072
	SHL-5	(K15) HZ1-OP2 (DC264)	3.26	133	0.026
	SHL-5	(K15) HZ2-OP2 (DC264)	3.43	130	0.026
	SHL+4	(K12) HZ1-OP2 (DT107)	3.38	236	0.047
	SHL-5	(K23) HZ1-OP1 (DA28)	3.16	339	0.067
	SHL+3	(R29)HH1 –OP1 (DC194)	2.89	340	0.068
	SHL+3	(K28) HZ1 -OP1(DG193)	3.33	218	0.043
	SHL+3	(K5) HZ3 -OP2(DT184)	3.35	131	0.026
	SHL-5	(K23) HZ2-OP1 (DA28)	3.04	322	0.064
	SHL+4	(K23) HZ3-OP1 (DT105)	3.28	291	0.058
	SHL+4	(K23) HZ2-OP1 (DT105)	3.28	252	0.050
	SHL+4	(K23) HZ1-OP1 (DT105)	3.35	234	0.046

	SHL+4	(K24) HZ2-OP1 (DT106)	3.21	363	0.072
	SHL+4	(K24) HZ3-OP1 (DT106)	3.20	326	0.065
	SHL+4	(K12) HZ3-OP1 (DT107)	3.21	241	0.048
	SHL+4	(R30) HH21-OP1 (DT105)	3.25	239	0.047
	SHL+4	(R29) HZ3-OP1 (DT106)	3.18	139	0.027
	SHL-5	(K27) HZ1-OP1 (DA27)	3.20	309	0.061
	SHL-5	(K27) HZ3-OP1 (DA27)	3.19	305	0.061
**H2B Tail-2**	SHL-3	(R29) HH12 -OP1 (DG46)	2.95	1573	0.314
	SHL-3	(K27) HZ1- OP1 (DT48)	2.87	312	0.062
	SHL-3	(K11) HZ2- OP2 (DA243)	3.45	176	0.035
	SHL-3	(K5) HZ1- OP1 (DT244)	3.03	81	0.016
	SHL+5.5	(K23) HZ3- OP1 (DA125)	3.16	236	0.047
	SHL+5.5	(K20) HZ3- OP1 (DG126)	3.31	226	0.045
	SHL-3.5	(K24) HZ2- OP1 (DT252)	3.35	152	0.030
	SHL+5	(K27) HZ3- OP1 (DT124)	3.07	505	0.101
	SHL+5	(K28) HZ2-OP1 (DA175)	3.12	618	0.123
	SHL+5	(K28) HZ1-OP1 (DA175)	3.12	596	0.119
	SHL+5	(K28) HZ3-OP1 (DA175)	3.18	569	0.113
	SHL-3.5	(K28) HZ3- OP1 (DT252)	3.00	984	0.196
	SHL-3	(R29) HH12- OP1(DG46)	2.98	1592	0.318
	SHL-3	(K11) HZ1- OP2 (DG242)	3.22	93	0.018
	SHL-3	(K11) HZ3- OP2 (DA243)	3.45	172	0.034
	SHL-3	(K11) HZ1- OP2 (DA243)	3.36	169	0.033
	SHL-3	(K11) HZ1- OP1 (DA243)	3.47	108	0.021
	SHL+5.5	(K16) HZ3- OP1 (DT127)	3.34	281	0.056
	SHL+5.5	(K20) HZ1- OP1 (DT126)	3.30	221	0.044
	SHL+5.5	(K20) HZ2- OP1 (DT126)	3.19	202	0.040
	SHL+5	(K23) HZ1- O2 (DC172)	3.19	314	0.062
	SHL+5	(K23) HZ2- O2 (DC172)	3.24	279	0.055
	SHL+5	(K23) HZ2- OP1 (DA174)	3.10	214	0.042
	SHL+5	(K23) HZ1- O4 (DA174)	3.40	188	0.037
	SHL+5	(K24) HZ3- OP2 (DA175)	3.47	92	0.018
	SHL-3.5	(K24) HZ3- OP1 (DT252)	3.28	145	0.029
	SHL-3.5	(K24) HZ1- OP1 (DT252)	3.19	124	0.024
	SHL+5	(K27) HZ1- OP1 (DT124)	3.07	496	0.099
	SHL+5	(K27) HZ2- OP1 (DT124)	3.08	453	0.090
	SHL+5	(K27) HZ1- OP1 (DA125)	3.23	370	0.074
	SHL+5	(K27) HZ3- OP1 (DA125)	3.21	356	0.071
	SHL+5	(K27) HZ2- OP1 (DA125)	3.35	335	0.067
	SHL-3.5	(K28) HZ2- OP1 (DT252)	3.10	977	0.195
	SHL-3.5	(K28) HZ2- OP1 (DT252)	3.07	964	0.192
	SHL-3	(K27) HZ2- OP1 (DT48)	2.98	282	0.056
	SHL-3	(K27) HZ1- OP1 (DT48)	2.87	297	0.059
	SHL-3	(K27) HZ3- OP1 (DT48)	2.91	312	0.062

Table S3. Hydrogen bonds between DNA and H2B tail residues for ACK 0.15 M salt concentration

ACK_H2B Tails 0.15M	SHL regions	Residues	Avg. distance (Å)	Frames	Fraction
H2B Tail-1	SHL-5	(K24) HZ3- OP1 (DC26)	3.36	109	0.021
	SHL-5	(K27) HZ3- OP1 (DC26)	3.35	73	0.014
	SHL+4	(K11) HZ3- OP1 (DT106)	3.36	175	0.035
	SHL+4	(K28) HZ1- OP1 (DT106)	3.04	282	0.056
	SHL-5	(R30) HH12- OP1 (DC26)	2.87	733	0.146
	SHL-5	(K23) HZ3- OP1 (DC25)	3.24	8	0.001
	SHL-5	(K24) HZ3- OP1 (DC25)	3.16	27	0.005
	SHL-5	(K24) HZ1- OP1 (DC25)	3.27	26	0.005
	SHL-5	(K24) HZ2- OP1 (DC25)	3.48	38	0.007
	SHL-5	(K24) HZ2- OP1 (DA27)	3.36	91	0.002
	SHL-5	(K24) HZ1- OP1 (DA27)	3.45	77	0.015
	SHL-5	(K24) HZ2- OP2 (DA27)	3.19	8	0.001
	SHL+4	(K24) HZ2- OP1 (DT106)	3.40	29	0.005
	SHL+4	(K24) HZ3- OP1 (DT106)	3.12	31	0.006
	SHL+4	(K24) HZ3- OP1 (DT106)	3.05	17	0.003
	SHL-5	(K27) HZ1- OP1 (DC26)	3.19	35	0.007
	SHL-5	(K27) HZ2- OP1 (DC26)	3.22	62	0.012
	SHL-5	(K27) HZ3- OP1 (DC26)	3.20	61	0.012
	SHL-5	(K27) HZ1- OP1 (DA27)	3.21	49	0.009
	SHL-5	(K27) HZ3- OP1 (DA27)	3.12	43	0.008
	SHL+4	(K27) HZ1- OP1 (DT106)	3.27	82	0.016
	SHL-5	(K28) HZ1- OP1 (DC26)	3.49	165	0.033
	SHL-5	(K28) HZ3- OP2 (DC26)	3.39	161	0.032
	SHL-5	(K28) HZ1- OP2 (DC26)	3.39	140	0.028
	SHL-5	(K28) HZ2- OP2 (DC26)	3.33	166	0.033
	SHL+4	(K28) HZ3- OP1 (DT106)	2.97	274	0.054
	SHL+4	(K28) HZ2- OP1 (DT106)	2.97	257	0.051
	SHL+3	(K27) HZ1- OP1(DT195)	3.36	72	0.014
	SHL+3	(K28) HZ1- OP1(DT195)	3.10	68	0.013
	SHL+3	(K24) HZ2- OP1(DG193)	3.27	53	0.016
	SHL+3	(K23) HZ3- OP1(DG193)	3.18	34	0.006
	SHL+3	(K16) HZ2- OP1(DC194)	2.87	33	0.006
	SHL+3	(K24) HZ1- OP1(DG193)	3.23	33	0.006
	SHL+3	(R29) HH22-OP2 (DA103)	2.87	1454	0.290
H2B Tail-2	SHL-3	(K23) HZ2- OP2 (DC47)	3.36	81	0.016
	SHL-3	(R30)HH21-OP2(DC47)	3.08	797	0.159
	SHL-3	(R29)HH22-OP1(DC47)	2.97	266	0.053
	SHL-3	(R29)HH21-OP1(DC47)	3.13	246	0.049
	SHL-3	(R30)HH21-OP2(DT48)	3.20	266	0.053
	SHL+5	(K24) HZ1- OP2 (DA174)	3.38	370	0.074
	SHL+5	(K27) HZ1 – OP1(DC173)	3.39	327	0.065
	SHL+5	(K27) HZ2 – OP1(DC173)	3.42	324	0.064
	SHL+5	(K27) HZ3 – OP1(DC173)	3.27	316	0.063
	SHL+5	(K24) HZ2 – OP2(DA174)	3.46	279	0.055
	SHL+5	(K24) HZ3 – OP1(DC173)	3.44	78	0.015
	SHL+5	(K24) HZ2 – OP1(DC173)	3.49	60	0.012
	SHL+5	(ACK5)HZ1–OP2(DC172)	3.32	34	0.006
	SHL-3	(K27) HZ1- OP1 (DT48)	3.46	105	0.021
	SHL-3.5	(K28) HZ1- OP1 (DT252)	3.47	397	0.079
	SHL-3	(R29) HH22- OP1 (DC47)	3.12	284	0.056
	SHL-3	(R30) HH21- OP1 (DC47)	3.15	825	0.165
	SHL-3	(K27) HZ2- OP2 (DT48)	3.24	99	0.019
	SHL-3	(K27) HZ3- OP1 (DT48)	3.34	95	0.019
	SHL-3	(K27) HZ3- OP2 (DT48)	3.12	74	0.0148
	SHL-3	(K27) HZ1- OP2 (DT48)	3.19	91	0.0182

	SHL-3	(ACK12)HZ1-OP2(DC47)	3.24	6	0.001
	SHL-3.5	(K28) HZ1- OP2 (DT252)	3.49	381	0.0762
	SHL+5	(K28) HZ2- OP1 (DA252)	3.47	30	0.006
	SHL+5	(K28) HZ1- OP1 (DA252)	3.28	14	0.002
	SHL-3	(R29) HH12- OP1 (DC47)	3.30	264	0.052
	SHL-3	(R29) HH12-OP1(DC47)	3.02	233	0.046
	SHL-3	(R30) HH22-OP2(DC47)	3.35	105	0.021
	SHL-3	(K27) HZ1- OP1 (DT48)	3.38	100	0.020
	SHL-3	(K27) HZ1- OP2 (DT48)	3.16	90	0.018
	SHL-3	(K16) HZ2- OP1 (DT45)	3.45	79	0.015
	SHL-3	(K23) HZ3- OP2 (DC47)	3.19	73	0.014
	SHL-3	(K27) HZ3- OP2 (DT48)	2.98	70	0.014
	SHL-3	(K16) HZ1- OP1(DT45)	3.32	66	0.013
	SHL-3	(K23) HZ1- OP2 (DC47)	3.37	48	0.009
	SHL-3	(K23) HZ1- OP1 (DC47)	3.28	37	0.007
	SHL-3	(K11) HZ1- OP1 (DT45)	3.32	36	0.007
	SHL-3	(R29) HH21- OP1 (DC47)	3.28	263	0.052

Table S4. Hydrogen bonds between DNA and H2B tail residues for WT 2.4 M salt concentration

WT_H2B Tails 2.4 M	SHL regions	Residues	Avg. distance (Å)	Frames	Fraction
H2B Tail-1	SHL-5	(K5) HZ1-OP1 (DC264)	3.25	62	0.012
	SHL-5	(K5) HZ2-OP2 (DC264)	3.40	55	0.011
	SHL-5	(K5) HZ2-OP1 (DC264)	3.36	54	0.010
	SHL-5	(K5) HZ1-OP2 (DC264)	3.47	53	0.010
	SHL-5	(K5) HZ3-OP2 (DC264)	3.24	66	0.013
	SHL+4	(K11) HZ1-OP2 (DT106)	3.34	348	0.069
	SHL+4	(K12) HZ3-OP2 (DT106)	3.43	499	0.099
	SHL-5	(K15) HZ1-OP1 (DC26)	3.25	549	0.109
	SHL+3	(K16) HZ3-OP1 (DC194)	2.82	1162	0.232
	SHL+3	(K28) HZ3-OP2 (DT195)	2.89	407	0.081
	SHL+3	(R29) HH22-OP1 (DG104)	2.96	106	0.021
	SHL-5	(R30) HH12-OP1 (DA27)	2.81	1717	0.343
	SHL+4	(K11) HZ2-OP1 (DT106)	3.10	330	0.066
	SHL+4	(K11) HZ2-OP2 (DT106)	3.12	304	0.060
	SHL-5	(K12) HZ1-OP1 (DA28)	3.24	339	0.067
	SHL-5	(K12) HZ2-OP1 (DA28)	3.28	337	0.067
	SHL-5	(K12) HZ3-OP1 (DA28)	3.27	346	0.069
	SHL+4	(K12) HZ1-OP2 (DT105)	3.46	443	0.088
	SHL+4	(K12) HZ2-OP2 (DT105)	3.35	441	0.088
	SHL-5	(K15) HZ3-OP1 (DC26)	3.37	526	0.105
	SHL-5	(K15) HZ2-OP1 (DC26)	3.34	508	0.101
	SHL-5	(K15) HZ2-OP2 (DA27)	3.27	327	0.065
	SHL-5	(K15) HZ1-OP2 (DA27)	3.22	300	0.060
	SHL-5	(K15) HZ3-OP1 (DA27)	3.20	289	0.057
H2B Tail-2	SHL+5	(K16) HZ2- OP1 (DA171)	3.30	63	0.012
	SHL+5	(K15) HZ3- OP2 (DA171)	2.87	8	0.001
	SHL+5.5	(K20) HZ2- OP1 (DT127)	3.19	111	0.022
	SHL+5.5	(K24) HZ2- OP1 (DC175)	2.90	203	0.040
	SHL+5.5	(K27) HZ1- OP1 (DG126)	2.84	1090	0.218
	SHL+5.5	(K28) HZ1- OP1 (DA174)	2.81	380	0.076
	SHL+5.5	(R29) HH11- OP1 (DA174)	2.83	355	0.071
	SHL+5.5	(K5) HZ2- OP1 (DC130)	2.84	13	0.002
	SHL+5.5	(K5) HZ1- OP1 (DC130)	2.82	11	0.002
	SHL+5	(K16) HZ3- OP1 (DA171)	3.32	62	0.012
	SHL+5	(K16) HZ1- OP1 (DA171)	3.34	53	0.010
	SHL+5	(K16) HZ2- OP2 (DA171)	3.15	39	0.007

	SHL+5	(K16) HZ3- OP2 (DA171)	3.24	35	0.007
	SHL+5.5	(K20) HZ3- OP1 (DT127)	3.30	107	0.021
	SHL+5.5	(K20) HZ3- OP1 (DA128)	3.13	104	0.020
	SHL+5.5	(K20) HZ2- OP1 (DA128)	3.22	100	0.020
	SHL-3	(R30) HH12- OP1 (DA250)	2.87	407	0.081

Table S5. Hydrogen bonds between DNA and H2B tail residues for ACK 2.4 M salt concentration

ACK_H2B Tails 2.4 M	SHL regions	Residues	Avg. distance (Å)	Frames	Fraction
H2B Tail-1	SHL-5	(K23) HZ2- OP1 (DC26)	3.10	389	0.077
	SHL-5	(K23) HZ3- OP1 (DC26)	3.09	332	0.066
	SHL-5	(K23) HZ1- OP1 (DC26)	3.10	318	0.063
	SHL-5	(K23) HZ3- OP2 (DC26)	3.49	245	0.049
	SHL-5	(K11) HZ3- OP2 (DA28)	3.16	237	0.047
	SHL-5	(K11) HZ1- OP2 (DA28)	3.21	228	0.045
	SHL-5	(K11) HZ2- OP2 (DA28)	3.23	220	0.044
	SHL-5	(K11) HZ1- OP2 (DA27)	3.04	218	0.043
	SHL-5	(K11) HZ1- OP2 (DA27)	3.10	204	0.040
	SHL-5	(K11) HZ2- OP2 (DA27)	3.09	202	0.040
	SHL-5	(K24) HZ3- OP2 (DC25)	3.26	138	0.027
	SHL-5	(K24) HZ1- OP1 (DC25)	3.17	136	0.027
	SHL-5	(ACK12) HZ1- OP2 (DA27)	3.06	115	0.023
	SHL-5	(K27) HZ1- OP1 (DC26)	3.45	58	0.011
	SHL-5	(ACK5) HZ1- OP2 (DA263)	3.25	98	0.019
	SHL-5	(ACK5) HZ1- OP2 (DT266)	3.37	64	0.012
	SHL+3	(K28) HZ3- OP2 (DT195)	3.38	839	0.167
	SHL+3	(K28) HZ1- OP2 (DT195)	3.38	814	0.162
	SHL+3	(K28) HZ2- OP2 (DT195)	3.39	765	0.153
	SHL+3	(K16) HZ2- OP1 (DG193)	2.95	160	0.032
	SHL+3	(K16) HZ3- OP1 (DG193)	2.95	148	0.029
	SHL+3	(K28) HZ3- OP2 (DC194)	3.25	84	0.016
	SHL+3	(K28) HZ1- OP2 (DC194)	3.20	72	0.014
	SHL+3	(R29) HH12- OP1(DC194)	3.03	4934	0.986
H2B Tail-2	SHL+5.5	(K28) HZ1- OP1 (DA125)	3.00	499	0.099
	SHL-3	(K11) HZ3- OP2 (DT48)	2.88	1352	0.270
	SHL-3	(K11) HZ1- OP2 (DT48)	2.86	1252	0.250
	SHL-3	(K11) HZ2- OP2 (DT48)	2.89	1236	0.247
	SHL-3	(R30) HH12- OP1 (DC49)	3.10	691	0.138
	SHL+5	(R30) HH11- OP1 (DT124)	3.05	553	0.110
	SHL+5.5	(K28) HZ2- OP1 (DA125)	3.02	469	0.093
	SHL-3	(R30) HH22- OP2 (DC49)	3.13	655	0.130
	SHL+5	(R30) HH12- OP1 (DT124)	3.22	22	0.004
	SHL-3	(K23) HZ2- OP1 (DG46)	3.09	357	0.071
	SHL+5	(K27) HZ1- OP1 (DC173)	3.30	595	0.119
	SHL+5	(K27) HZ2- OP1 (DC173)	3.27	576	0.115
	SHL-3	(K23) HZ1- OP1 (DG46)	3.08	338	0.067
	SHL+5	(K27) HZ3- OP1 (DC173)	3.25	576	0.115
	SHL+5	(K24) HZ3- OP2 (DA175)	3.12	301	0.060
	SHL-3	(K23) HZ3- OP1 (DG46)	3.06	337	0.0674
	SHL+5	(K24) HZ1- OP2 (DA175)	3.16	299	0.059
	SHL-3	(R30) HH12- OP1 (DC49)	3.06	30	0.006
	SHL-3	(R30) HH11- OP1 (DC49)	3.24	200	0.040
	SHL+5	(K23) HZ1- OP2 (DA174)	3.25	115	0.023
	SHL+5	(K23) HZ2- OP2 (DA174)	3.23	114	0.022
	SHL+5	(K23) HZ3- OP2 (DA174)	3.37	94	0.018
	SHL+5	(K28) HZ3- OP1 (DC173)	3.04	46	0.009
	SHL+5	(K28) HZ1- OP1 (DC173)	3.06	46	0.009

	SHL-3.5	(K24) HZ2- OP1 (DT253)	3.11	506	0.101
	SHL-3.5	(K24) HZ3- OP1 (DT253)	3.07	500	0.100
	SHL-3.5	(K24) HZ3- OP1 (DT252)	2.93	374	0.074
	SHL-3	(R30) HH11- OP1 (DG251)	3.34	265	0.053

Table S6. Helix propensity of the H2B N-terminal tail conformations extracted from PCA free-energy basins for 0.15 M salt concentration.

Systems	Conformations	Helix Propensity	No. of Contacts
**WT_H2B Tail-1**	I	0.26	104
	II	0.23	96
**ACK_H2B Tail-1**	I	0.20	20
	II	0.13	28
	III	0.00	33
**WT_H2B Tail-2**	I	0.023	132
	II	0.088	183
**ACK_H2B Tail-2**	I	0.25	43
	II	0.27	37

Table S7. Helix propensity of the H2B N-terminal tail conformations extracted from PCA free-energy basins for 2.4 M salt concentration.

Systems	Conformations	Helix Propensity	No. of Contacts
**WT_H2B Tail-1**	I	0.003	163
	II	0.00	162
**ACK_H2B Tail-1**	I	0.002	29
	II	0.00	36
**WT_H2B Tail-2**	I	0.00	192
	II	0.002	214
**ACK_H2B Tail-2**	I	0.02	50
	II	0.00	34

Supporting Movies

Movie S1: Wild Type (WT) unacetylated H2B tails with nucleosomal DNA. Both H2B tails (cyan) with DNA (green) throughout the simulation in proximity of DNA.

Movie S2: Acetylated (ACK) H2B tails with nucleosomal DNA. Acetylated H2B tails (orange) with DNA (green) throughout the simulation. Tails are released from DNA more often.

Movie S3: Wild Type (WT) unacetylated H2B tails with nucleosomal DNA salt bridge formation. The example of favorable DNA phosphate and H2B positively charged residues interaction via hydrogen bond formation. The WT H2B tail-2 exhibit hydrogen bonds between R29 with phosphate backbone of DG46 and between K11 with the phosphate backbone of DG242.

Movie S4: Acetylated (ACK) H2B tails with nucleosomal DNA salt bridge formation. The example of favorable DNA phosphate and H2B positively charged residues interaction via hydrogen bond. The ACK H2B tail-1 exhibit hydrogen bonds between K28 with phosphate backbone of DA27.

Movie S5: Wild Type (WT) unacetylated H2B tails with nucleosomal DNA. H2B tail-2 has shown when collapses on DNA, it is mostly to the SHL-3 region of DNA.

References

(1) Roe, D. R.; Cheatham, T. E., III. Ptraj and Cpptraj: Software for Processing and Analysis of Molecular Dynamics Trajectory Data. *Journal of Chemical Theory and Computation* **2013**, *9*, 3084-3095. DOI: 10.1021/ct400341p.

(2) Michaud-Agrawal, N.; Denning, E. J.; Woolf, T. B.; Beckstein, O. Software News and Updates Mdanalysis: A Toolkit for the Analysis of Molecular Dynamics Simulations. *Journal of Computational Chemistry* **2011**, *32*, 2319-2327. DOI: 10.1002/jcc.21787.

(3) Kollman, P. A.; Massova, I.; Reyes, C.; Kuhn, B.; Huo, S.; Chong, L.; Lee, M.; Lee, T.; Duan, Y.; Wang, W.; et al. Calculating Structures and Free Energies of Complex Molecules: Combining Molecular Mechanics and Continuum Models. *Acc Chem Res* **2000**, *33*, 889-897. DOI: 10.1021/ar000033j From NLM.

(4) Wang, E.; Sun, H.; Wang, J.; Wang, Z.; Liu, H.; Zhang, J. Z. H.; Hou, T. End-Point Binding Free Energy Calculation with Mm/Pbsa and Mm/Gbsa: Strategies and Applications in Drug Design. *Chemical Reviews* **2019**, *119*, 9478-9508. DOI: 10.1021/acs.chemrev.9b00055.

(5) Kosarim, N. A.; Armeev, G. A.; Kirpichnikov, M. P.; Shaytan, A. K. Analysis of Ion Atmosphere around Nucleosomes Using Supercomputer Md Simulations. *Supercomputing Frontiers and Innovations* **2022**, *9*, 56-67. DOI: 10.14529/jsfi220205 (acccessed 2023/10/11).

(6) Smith, A. M.; Lee, A. A.; Perkin, S. The Electrostatic Screening Length in Concentrated Electrolytes Increases with Concentration. *J Phys Chem Lett* **2016**, *7*, 2157-2163. DOI: 10.1021/acs.jpclett.6b00867 From NLM.

(7) Lee, N.; Thirumalai, D. Dynamics of Collapse of Flexible Polyelectrolytes in Poor Solvents. *Macromolecules* **2001**, *34*, 3446-3457. DOI: 10.1021/ma001604q.

(8) Schiessel, H.; Pincus, P. Counterion-Condensation-Induced Collapse of Highly Charged Polyelectrolytes. *Macromolecules* **1998**, *31*, 7953-7959. DOI: 10.1021/ma980823x.

(9) Ha, B.-Y.; Thirumalai, D. Conformations of a Polyelectrolyte Chain. *Physical Review A* **1992**, *46*, R3012-R3015. DOI: 10.1103/PhysRevA.46.R3012.

(10) Potoyan, D. A.; Papoian, G. A. Energy Landscape Analyses of Disordered Histone Tails Reveal Special Organization of Their Conformational Dynamics. *Journal of the American Chemical Society* **2011**, *133*, 7405-7415. DOI: 10.1021/ja1111964.
